# Competitive inhibition and mutualistic growth in co-infections: deciphering *Staphylococcus aureus–Acinetobacter baumannii* interaction dynamics

**DOI:** 10.1093/ismeco/ycae077

**Published:** 2024-06-10

**Authors:** Sandra Timme, Sindy Wendler, Tilman E Klassert, Joao Pedro Saraiva, Ulisses Nunes da Rocha, Manuel Wittchen, Sareda Schramm, Ralf Ehricht, Stefan Monecke, Birgit Edel, Jürgen Rödel, Bettina Löffler, Maria Soledad Ramirez, Hortense Slevogt, Marc Thilo Figge, Lorena Tuchscherr

**Affiliations:** Applied Systems Biology, Leibniz Institute for Natural Product Research and Infection Biology, Hans Knöll Institute, Friedrich Schiller University Jena, Leibniz Centre for Photonics in Infection Research (LPI), D-07743 Jena, Germany; Institute of Medical Microbiology, Jena University Hospital, D-07740 Jena, Germany; Respiratory Infection Dynamics, Helmholtz Centre for Infection Research – HZI, D-38124 Braunschweig, Germany; Department of Respiratory Medicine and Infectious Diseases, Hannover Medical School, German Center for Lung Research (DZL), BREATH, D-30625 Hannover, Germany; Department of Environmental Microbiology, Helmholtz Centre for Environmental Research-UFZ, D-04318 Leipzig, Germany; Department of Environmental Microbiology, Helmholtz Centre for Environmental Research-UFZ, D-04318 Leipzig, Germany; Center for Biotechnology, Bielefeld University, D-33501 Bielefeld, Germany; Department of Biological Science, Center for Applied Biotechnology Studies, California State University, 800 N State College Blvd, Fullerton, CA 92831, United States; Leibniz Institute of Photonic Technology, Leibniz Centre for Photonics in Infection Research (LPI), D-07745 Jena, Germany; Institute of Physical Chemistry, Friedrich Schiller University Jena, Leibniz Centre for Photonics in Infection Research (LPI) , D-07743 Jena, Germany; Leibniz Institute of Photonic Technology, Leibniz Centre for Photonics in Infection Research (LPI), D-07745 Jena, Germany; Institute for Medical Microbiology and Virology, Dresden University Hospital, Dresden, Germany; Institute of Medical Microbiology, Jena University Hospital, D-07740 Jena, Germany; Institute of Medical Microbiology, Jena University Hospital, D-07740 Jena, Germany; Institute of Medical Microbiology, Jena University Hospital, D-07740 Jena, Germany; Cluster of Excellence Balance of the Microverse, Friedrich Schiller University Jena, D-07743 Jena, Germany; Department of Biological Science, Center for Applied Biotechnology Studies, California State University, 800 N State College Blvd, Fullerton, CA 92831, United States; Respiratory Infection Dynamics, Helmholtz Centre for Infection Research – HZI, D-38124 Braunschweig, Germany; Department of Respiratory Medicine and Infectious Diseases, Hannover Medical School, German Center for Lung Research (DZL), BREATH, D-30625 Hannover, Germany; Applied Systems Biology, Leibniz Institute for Natural Product Research and Infection Biology, Hans Knöll Institute, Friedrich Schiller University Jena, Leibniz Centre for Photonics in Infection Research (LPI), D-07743 Jena, Germany; Cluster of Excellence Balance of the Microverse, Friedrich Schiller University Jena, D-07743 Jena, Germany; Institute of Microbiology, Faculty of Biological Sciences, Friedrich Schiller University, D-07743 Jena, Germany; Institute of Medical Microbiology, Jena University Hospital, D-07740 Jena, Germany

**Keywords:** bacterial co-infections, Staphylococcus aureus, Acinetobacter baumannii, mathematical modeling, extended logistic growth model, cooperation, competition

## Abstract

*Staphylococcus aureus (Sa)* and *Acinetobacter baumannii (Ab)* are frequently co-isolated from polymicrobial infections that are severe and refractory to therapy. Here, we apply a combination of wet-lab experiments and *in silico* modeling to unveil the intricate nature of the *Ab*/*Sa* interaction using both, representative laboratory strains and strains co-isolated from clinical samples. This comprehensive methodology allowed uncovering *Sa's* capability to exert a partial interference on *Ab* by the expression of phenol-soluble modulins. In addition, we observed a cross-feeding mechanism by which *Sa* supports the growth of *Ab* by providing acetoin as an alternative carbon source. This study is the first to dissect the *Ab*/*Sa* interaction dynamics wherein competitive and cooperative strategies can intertwine. Through our findings, we illuminate the ecological mechanisms supporting their coexistence in the context of polymicrobial infections. Our research not only enriches our understanding but also opens doors to potential therapeutic avenues in managing these challenging infections.

## Graphical Abstract

**Graphical Abstract:**
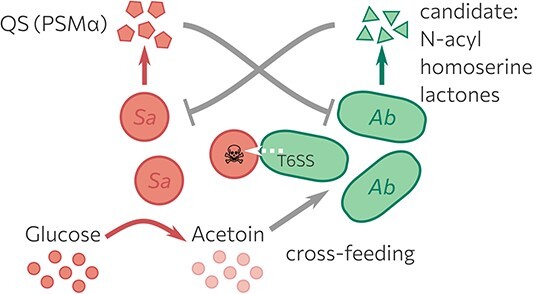

Summary of the *Ab*/*Sa* interaction during co-infections. Contact-independent mechanisms such as the secretion of quorum sensing (QS) molecules like phenol-soluble molecule α (PSMα) by *Sa* or N-homoserine lactones by *Ab* drive competitive interactions. In addition, *Ab *can compete with *Sa* via the expression of the type VI secretion system (T6SS) and can affect *Sa* in a contact-dependent manner. In contrast, both species cooperate by the cross-feeding of the alternative carbon source acetoin, which is produced by *Sa* and consumed by *Ab*.

## Introduction

Polymicrobial communities occur in wound and systemic infections, and are associated with a poor prognosis for the patient and an increased mortality rate. Interactions between different microbial species can influence pathogenic behavior such as their ability to cause disease, form biofilm, resist antibiotics, and elicit responses from the host immune system [1, 2]. Two common modes of interspecies interactions are co-existence and competition. Co-existence or mutualistic interactions facilitate cohabitation between two or more microbes, where at least one of them benefits from the interaction [1, 2]. For example, a cooperative interaction between *Staphylococcus aureus* (*Sa*) and *Pseudomonas aeruginosa* through the cross-feeding of acetoin in cystic fibrosis in the lung has been described recently [3], as well as a cooperative interaction between *Escherichia coli* and *Acinetobacter baumannii* (*Ab*) and *P. aeruginosa* was found [4]. Here, they showed that in dual biofilms a plasmid harboring a carbapenemase resistance gene can be transferred from *E. coli* to *Ab or P. aeruginosa*. Thus, microorganisms receiving these genes are more resistant to β-lactam antibiotics. Another cooperative interaction was reported for *Ab* and *Klebsiella pneumonia* co-infections through cross-protection and cross-feeding [5]. These examples show that coexisting infections are more difficult to eradicate than infections caused by either species alone. In polymicrobial infections, competitive interactions can occur in exploitative or interference modes[6] as well as by colonization resistance [4]. Exploitative interactions are taking place when one microbe depletes its surroundings of nutrients, thereby depriving potential competitors. Interference competition refers to specific metabolites produced by one of the microbes, such as antimicrobial toxins that inhibit or reduce the growth of one or more microbes [7, 8, 9]. Colonization resistance—a term originating from microbiome studies—describes how the host microbiota prevents invading pathogens from establishing [10]. These interactions involve both contact-independent as well as contact-dependent mechanisms that are either mediated by the secretion of specific compounds or physical cell contact, respectively. During physical cell contact, specific membrane proteins act as delivery systems for effector molecules such as specific toxins [11, 12]. Such contact-dependent interaction mechanisms in bacteria have been extensively reviewed [13, 14].

*Sa* and *A. baumannii* (*Ab*) are frequently isolated from respiratory, blood, urinary, skin and soft tissue, and diabetic foot infections [11, 12, 15, 16, 17], suggesting that both pathogens are present in the same niche and can interact *in vivo*. *Sa* co-infections with other bacterial and fungal pathogens are reviewed by Mariani and Galvan. Furthermore, Li et al. showed that the majority of polymicrobial infections are co-infections by *Sa* and *Ab* [18]. Both pathogens are part of the ESKAPE group and are an important representation of the escalating challenges of antimicrobial resistance [19]. However, the intricacies characterizing their mutual dynamic interactions remain elusive. Moreover, both pathogens are at the top of the World Health Organization priority list of critical bacterial species according to their mortality implications, healthcare burden, community pervasiveness, resistance prevalence, longitudinal resistance trends, transmissibility, preemptive potential within the community and healthcare settings, as well as therapeutic feasibility [20].

A recent study by Hardy et al. showed that several strains of *Ab* exert contact-dependent bacteriostatic or bactericidal effects on *Sa* growth [21]. On the contrary, a potential protective effect of a carbapenemase *Ab* producing strain on *Sa* was observed in the presence of β-lactam antibiotics, particularly under low doses of meropenem [22]. Moreover, cooperative interactions were observed in biofilm and in the expression of virulence factors [23, 24]. However, the intrinsic interaction between both pathogens was not yet examined in detail [21].

Systems biology —the dynamic interplay between wet-lab and *in silico* studies—has been applied in the past in the context of infection research [25, 26, 27]. Various approaches for modeling and simulation of cell population dynamics have been extensively reviewed by Charlebois and Balázsi [28]. In this study, we pursued a multidisciplinary approach to identify general interaction principles of *Sa* and *Ab* during co-infections with the focus on (i) their interaction via secreted compounds that drives interaction in a contact-independent manner and (ii) their physical interaction, i.e. contact-dependent mechanisms. Thus, to investigate general interaction principles of *Sa* and *Ab*, we conducted various wet-lab experiments using selected laboratory strains. Based on these results, we developed an extended logistic growth model based on ordinary differential equations (ODE). This enabled us to gain a quantitative characterization of the time-resolved growth curves and to compare model parameters for various conditions. Furthermore, changes in transcription and metabolic pathways triggered for each pathogen in the presence of the other pathogen were investigated. To exploit the biological variation observed in the clinical setting, we also performed various wet-lab experiments using pairs of clinical strains isolated from co-infection sites and also quantitatively compared their growth parameters with the aid of mathematical modeling.

Here we studied intrinsic pathogen–pathogen interactions of two commonly co-isolated microorganisms in polymicrobial clinical samples that contribute to the outcome of the infection. Our results reveal several molecules that drive the *Ab*/*Sa* interaction and are promising candidates for future targets in the treatment of such co-infections.

## Materials and methods

### Collection of *A. baumannii* and *S. aureus* isolates

The bacterial isolates were collected during routine diagnostic work from the Jena University Hospital. Identification procedures were performed in our lab by Vitek (Biomerieux, Germany). The corresponding patients resided in Thuringia and the adjacent federal states (Saxony-Anhalt, Bavaria, and Hessen). The isolates were divided into *S. aureus* (*Sa*) and *A. baumannii* (*Ab*) (Supplementary Table 1).

### Bacterial cultivation

#### Cultivation in CFCM

Strains were cultivated overnight in TSB (Tryptone Soy Broth from Oxoid) at 37°C with agitation. The supernatant from the overnight culture (ONC) was collected by centrifugation (5000 g, 4°C, 10 min for *Sa* and 30 min for *Ab*) and filtered through a sterile 0.2-μm filter (Millex, Merck, Darmstadt, Germany).

#### Strains

ONCs were diluted in TSB to an optical density (OD) at 578 nm (OD578nm) of 1 and incubated for 3 h at 37°C with agitation. After incubation, the bacterial culture was washed with PBS (DPBS, no calcium, no magnesium, from Life Technologies, Darmstadt, Germany) and then diluted to an OD578nm of 0.05 in either TSB or 100% Cell-Free Conditioned Medium (CFCM).

#### Co-cultivation

For co-cultivation, overnight bacterial cultures from each strain in TSB were diluted to a concentration of 5 × 10^7^ CFU/ml in TSB (with each culture contributing 50%). The monoculture of each strain, with a concentration of 10 × 10^7^ CFU/ml, was used as a control.

### Growth curves

#### Preparing growth curves in CFCM

The growth curves in CFCM were prepared following the “cultivation in CFCM” protocol. After adjusting the OD at 578 nm (OD578nm) to 0.05 for each bacterial culture in either TSB or 100% CFCM, the samples were distributed into a 96-well plate (200 μl per well in triplicate) and incubated at 37°C with orbital agitation (3 mm). Turbidity (OD578nm) was measured every 30 min for 24 h using a plate reader (Tecan Infinite M200 Pro, Crailsheim, Germany). After 24 h of incubation, all samples were serially diluted and plated on blood agar plates. Colonies were counted after incubating for 24 h at 37°C.

#### For co-cultivated growth curves

In the case of co-cultivated growth curves, ONCs from each strain in TSB were diluted to a concentration of 5 × 10^7^ CFU/ml in TSB, with each culture contributing 50%. The monoculture of each strain was used as a control, with a concentration of 10 × 10^7^ CFU/ml. These cultures were then distributed into a 96-well plate (200 μl per well in triplicate) and incubated at 37°C with orbital agitation (3 mm). Turbidity (OD578nm) was measured every 30 min for 24 h. After 24 h of incubation, all samples were diluted and plated on both blood agar and Drigalski plates (a selected medium for Gram-negative bacteria). Colonies were counted after incubating for 24 h at 37°C.

#### For growth curves with treated CFCM

To investigate growth curves with treated CFCM, CFCM from *Sa* or *Ab* strains were prepared as described earlier. For heat treatment, the untreated CFCM was incubated at 95°C for 1 h. For proteinase K treatment, 10 μl of a 20 mg/ml proteinase K solution (Promega) was added to 1 ml of untreated CFCM and incubated at 37°C for 1 h. Proteinase K was subsequently inactivated at 95°C for 30 min. For trypsin treatment, 20 μl of a 20 mg/ml trypsin solution (Sigma) was added to 1 ml of untreated CFCM and incubated at 37°C for 1 h. Trypsin was inactivated at 95°C for 30 min. To measure growth curves, a 3 h bacterial culture with an initial OD578nm of 0.05 was added to 1 ml of TSB, untreated CFCM, or treated CFCM. These samples were then distributed into a 96-well plate (200 μl per well) in triplicate and incubated at 37°C with orbital agitation (3 mm). Turbidity (OD578nm) was measured every 30 min for 24 h using a plate reader (Tecan Infinite M200 Pro). After 24 h of incubation, all the samples were plated on blood agar plates to enumerate CFU/ml.

#### RNA sequencing

For RNA seq, the strains *Sa* USA300 and *Ab* A118 were cultivated, as described in “cultivation in supernatant”. After 3 h, 1 ml of bacterial culture from monoculture (TSB) or in CFCM, was mixed with 1 ml RNA-Protect (QIAGEN) and incubated for 5 min at RT. Samples were centrifuged at 5000 g for 10 min and RNA was extracted. For lysis, lysing beads (ZR BashingBead Lysis Tubes (0.1 and 0.5 mm; Zymo Research, Freiburg, Germany) and RNA-Pro Solution (RNAPro Solution, MP Biomedicals, Eschwege, Germany) were used. For the extraction of RNA, the Kit from PeqLab (peqGOLD Total RNA Kit, VWR Peqlab, Darmstadt, Germany) was used. RNA Seq was performed by using Kits from Ilumina (Berlin, Germany). Total RNA Library Construction was performed using the llumina® Stranded Total RNA Prep Kit (Ref# 20040525; Ilumina, Berlin, Germany). In short, 100 ng of total RNA were used as input for initial rRNA depletion with directed probes. The RNA was then fragmented and denatured, and reverse transcribed by first strand cDNA synthesis, followed by second-strand cDNA synthesis to conserve the strandness. After 3’end adenylation, anchors and indexes were attached to the library fragments. Barcoding was performed using the IDT® for Illumina® RNA UD Indexes Set A, Ligation (Ref# 20040553, Ilumina, Berlin, Germany). Final library fragments were purified using standard AMPURE XP Beads protocols (Ref# A63881; Beckman, Krefeld, Germany), and quality checked for size and molarity using a Tape Station 2200 (Agilent, Waldbronn, Germany) and the D1000 ScreenTapes (Ref# 5067-5582; Agilent, Waldbronn, Germany) and Reagents (Ref# 5067-5583; Agilent, Waldbronn, Germany). Equimolarly pooled libraries were then run on an SP flowcell v. 1.5 (Ref# 20028401; Agilent, Waldbronn, Germany) on a NovaSeq 6000 apparatus (Ilumina, Berlin, Germany) for 100 cycles.

#### Differential gene expression analysis

The cleaned reads were mapped onto the genome of the corresponding reference genomes of *Ab* A118 and *Sa* USA300 (NCBI Accessions ASM1467273v1 and ASM1346v1) and were mapped with bowtie2[29] (default parameters). To explore differential gene expression, we compared each strain growing in TSB with its growth in CFCM obtained from the other pathogen. For differential expression analysis the reads belonging to genes of four replicates per condition were counted with featureCounts[30] and tested for differential expression with DESeq2[31] using default settings. Genes with a *P*-value below 0.05 and a log 2 fold-change (logFC) above +1.0 or below −1.0, respectively, were considered to be differentially transcribed under the examined conditions.

#### Proteomics analysis

For Proteomics analysis *Sa* USA300 and *Ab* A118 were cultivated in 50 ml TSB at 37°C with agitation. As control pure TSB was used. After 3 and 24 h, 15 ml culture/TSB were centrifuged for 20 min, 5000 rpm and 4°C. Supernatant was filtrated through a 0.2 μm filter. Filtrated and sterile supernatant was quickly added to liquid nitrogen for 15 min and immediately stored at −80°C. The extraction of proteins, preparation of samples and proteome analysis was performed by BGI TECH SOLUTIONS (HONGKONG, China). Details are summarized in Supplementary File Proteomics.

#### Acetoin measurement

Strains were cultivated following the protocol outlined in “cultivation in CFCM.” These strains, with an initial OD578nm of 0.05, were incubated at 37°C in either TSB or CFCM. After incubation periods of 0, 1, 2, 3, and 4 h, 1 ml of supernatant was collected from each sample. Each sample underwent centrifugation at 10 000g for 10 min at 4°C.

From each supernatant, the acetoin concentration was quantified using a modified Voges-Poskauer test in 96-well plates [3]. For this measurement, 25 μl of the sample or an acetoin standard (ranging from 0.25 to 1.5 mM dissolved in water, cat. #40127-U; Sigma Aldrich, Taufkirchen, Germany) was sequentially mixed with 17.5 μl of Creatine (0.5% w/v in water, #C0780-10MG; Sigma Aldrich, Taufkirchen, Germany), 25 μl of α-naphtol (5% w/v in 95% EtOH, #KK14.1; Carl Roth, Karlsruhe, Germany), and 25 μl of KOH (40% w/v in water, #7986.1; Carl Roth, Karlsruhe, Germany). The samples or standards were thoroughly mixed, and the OD at 560 nm was measured after 15 min of incubation at room temperature.

#### CAMP test

*Sa* strains in this study, along with RN4220, were cultivated in 10 ml of TSB for 17–20 h at 37°C with agitation. For RN4220, an ONC was streaked in the center of a blood agar plate and then incubated for 5 h at 37°C. For the strains, ONCs were streaked vertically to RN4220, and the plate was subsequently incubated for an additional 24 h at 37°C. After a total of 24 h of incubation, the plates were placed in a 4°C environment for 1–2 h. Following this cooling period, hemolysis was visually inspected.

#### Detection of T6SS presence

To determine the presence of T6SS, a colony PCR was performed. Three bacterial colonies were selected from blood agar plates for all *Ab* strains. They were resuspended in 1 ml of PBS and lysed using ZR BashingBead Lysis Tubes (0.1 and 0.5 mm, #S6012-50; Zymo Research, Freiburg, Germany). The lysate was then homogenized using a SpeedMill PLUS (230 V—Homogenizator, Analytik Jena AG, Jena, Germany).

PCR was conducted using the HotStarTaq Master Mix Kit (250 U, #203443; Hilden, Qiagen). For T6SS detection, the following primers were utilized:

- Forward primer: 5′ GAA AGA TAT ATA CGT TGA GTT TCG 3‘
- Reverse primer: 5’ CTG CGT AAG AAG CTG TAT TAT TAG 3‘

The thermal cycling profile included an initial denaturation step at 95°C for 15 min, followed by 32 cycles of denaturation at 94°C for 30 s, primer annealing at 55°C for 90 s, and primer elongation at 72°C for 90 s. The cycling was completed with a final extension step at 72°C for 10 min. PCR products were electrophoresed in a 1.5% agarose gel and stained with ethidium bromide for visualization.

#### Microarray-based characterization of *S. aureus* isolates

Genotyping of all *Sa* isolates was performed using microarray-based assays (FZMB, Bad Langensalza, Thuringia Germany). The array covers 333 different targets related to ~170 different genes and their allelic variants. Thus, virulence and resistance factors as well as typing markers can simultaneously be detected. Furthermore, the assignment of isolates to known strains and clonal complexes is possible.

Detailed protocols, primer and probe sequences have been described previously [32, 33]. In short, *Sa* was cultivated on Colombia blood agar and harvested after overnight incubation. Following DNA preparation, a linear amplification was performed using one specific primer per target. In this step, biotin-16-dUTP was randomly incorporated into the amplicons in this step. After incubation and washing steps, hybridization of the labeled amplicons to probes immobilized on the array was performed, followed by incubation with streptavidin horseradish peroxidase. Addition of a dye generated local dye precipitations at those probes that bound a labeled amplicon. Microarrays were then photographed and analyzed using a designated reading device (InterVision, Bad Langensalza).

#### Survival ratio

Measured CFU counts of two independent experiments ${exp}1$ and ${exp}2$ were used to calculate the survival ratio $SR$ of strain $s$ with


$$ {SR}_{{exp}2}^{{exp}1}(s)=\frac{CF{U}^{{exp}1}- CF{U}^{{exp}2}}{CF{U}^{{exp}2}}, $$

which is the relative change in the CFU counts of ${exp}1$ with respect to ${exp}2$.

### *In silico* model description

For quantification of *Ab/Sa* interaction dynamics, we developed a mathematical model for bacterial growth in monocultures (CFCM, TSB) as well as in co-cultures, which is based on ODE. Bacterial growth was modeled using the following extended logistic growth model:


(1) \begin{equation*} \frac{d{B}_s}{dt}={r}_g^S\cdot{B}_s\cdot \left(1-\frac{B_s}{C_s(t)}\right), \end{equation*}

with $B$ representing the bacterial population size of the species $s\in \left( Ab, Sa\right)$ in terms of the OD. The population increases with growth rate ${r}_g^S$ and is limited by the time-variable carrying capacity ${C}_s(t)$. The carrying capacity describes the ability of the medium to maintain a certain population size. This can depend on various factors such as nutrient availability or the presence of growth promoting or growth limiting molecules like toxins. As the availability of nutrients and the presence of other factors can change over time, we modeled the carrying capacity as a function of time $t$ as follows:


(2) \begin{equation*} {C}_{s=\left\{ AB, SA\right\}}(t)=\frac{C_{end}^s+{C}_{start}^s}{2}+\frac{C_{end}^s-{C}_{start}^s}{2}\cdot \tanh \left({g}_s\cdot \left( time-{p}_s\right)\right), \end{equation*}

where ${C}_{start}^s$ and ${C}_{end}^s$ mark the values of the carrying capacity at the beginning and the end of the experiment, respectively. Thus, for ${C}_{start}^s>{C}_{end}^s$, the carrying capacity will decrease over time and vice versa. Furthermore, ${p}_s$ defines the time point at which the inflection point of the ${tanh}$ function is reached, while ${g}_s$ defines the slope at this point. For $g=0$, the carrying capacity ${C}_s$ will obtain the constant value of ${C}_s(t)=\left({C}_{end}^s+{C}_{start}^s\right)/2$. In that case, ${C}_{start}^s$ and ${C}_{end}^s$ are not uniquely identifiable as various combinations can yield the same value for ${C}_s(t)$ and the model insensitive toward $p$. Similarly, for ${C}_{start}^s\approx{C}_{end}^s$, the model parameters ${p}_s$ and ${g}_s$ become insensitive.

While, for monoculture experiments (in TSB or in CFCM), only the growth of one species is simulated, for co-culture experiments, the growth for both sub-populations of *Sa* and *Ab* was simulated. Thus, the total population size is given by


(3) \begin{equation*} {B}_{total}(t)={B}_{AB}(t)+{B}_{SA}(t). \end{equation*}

### *In silico* model numerical simulation

The model implementation was done in *Python* and numerical integration was performed using the *Python* package *SciPy* with the solver *lsoda*. The numerical integration of the ODE was performed for the initial conditions of the present species in the respective experiment, i.e. for monoculture experiments, this was the initial measured OD value, and for co-cultivation experiments, this value was halved for each species, since the concentration of both species was adjusted to be the same in these experiments. The integration was performed for the duration of the experiment, i.e. 24 h.

### *In silico* model parameter estimation

In order to estimate the model parameters, we fitted both models to the respective experimental data for various conditions. Parameter estimation was done using *simulated annealing* using 100 starting points and 1000 iterations for each starting point. For this, we used the *Python* package *SciPy.optimize* and utilized the dual annealing algorithm without additional local search[34] (URL: http://www.scipy.org/). During each parameter estimation step, the model was integrated with the respective parameter configuration (see *In silico* model numerical simulation). The agreement between the simulated growth curves $sim$ and the experimental data ${exp}$ was evaluated using the Sum-of-Squares-Error (SSE), which was minimized during the parameter estimation procedure [35, 36, 37, 38, 39].

For monoculture experiments, starting points were chosen using Latin Hypercube sampling with parameter ranges as given in Supplementary Table 10. The SSE was defined as follows:


(4) \begin{equation*} SSE\left(\overrightarrow{p}\right)=\kern0.5em \sum_{t=0}^{t_{max}}{\left({exp}(t)- sim(t)\right)}^2, \end{equation*}

For co-cultivation experiments, starting points were generated using the estimated parameters for each species in the CFCM of the other species. The SSE was defined as follows:


(5) \begin{align*}& SSE\left(\overrightarrow{p}\right)=\kern0.5em \sum_{t=0}^{t_{max}}{\left(\exp (t)- sim(t)\right)}^2\nonumber\\&+{\left(\frac{CF{U}_{\mathrm{Ab}}^{\exp, \kern0.75em {\mathrm{t}}_{max}}}{CF{U}_{\mathrm{Ab}}^{\exp, \kern0.75em {\mathrm{t}}_{max}}+ CF{U}_{\mathrm{Sa}}^{\exp, \kern0.75em {\mathrm{t}}_{max}}}-\frac{CF{U}_{\mathrm{Ab}}^{\mathrm{sim},\kern0.75em {\mathrm{t}}_{max}}}{CF{U}_{\mathrm{Ab}}^{\mathrm{sim},\kern0.75em {\mathrm{t}}_{max}}+ CF{U}_{\mathrm{Sa}}^{\mathrm{sim},\kern0.75em {\mathrm{t}}_{max}}}\right)}^2\!, \end{align*}

including not only measured growth curves but also the ratio of CFU counts after 24 h of co-cultivation (Supplementary Fig. 1).

### Statistical analysis

Statistical tests are described in the respective figure captions.

### Data availability

The raw RNA sequencing data in fastq format have been deposited in the Sequence Read Archive at the National Center for Biotechnology Information (NCBI) and can be accessed via the NCBI homepage (https://www.ncbi.nlm.nih.gov/; accession number: GSE250252).

Simulation data are available from https://asbdata.hki-jena.de/TimmeEtAl2023_Sa-Ab_CoInfection/Simulation_Data.

### Code availability

Code is available under https://github.com/applied-systems-biology/SaAbCoInfection.git.

## Results

**Figure 1:**
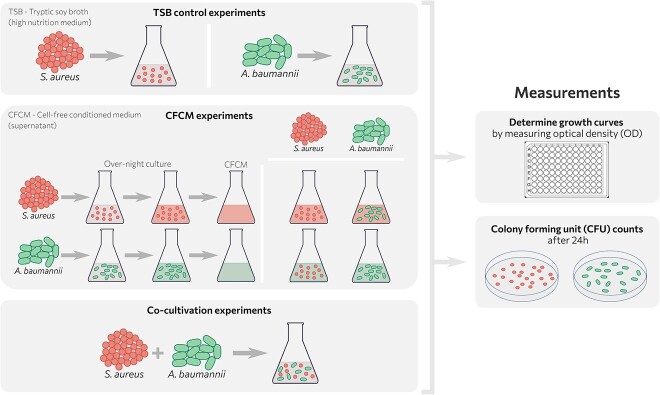
The experimental design of the study includes: (I) the control experiments, where each strain was grown in tryptic soy broth (TSB); (II) CFCM experiments, where each strain was grown overnight to obtain by centrifugation the CFCM. Each strain was cultivated in TSB, CFCM of the other pathogen or in its own CFCM; (III) co-cultivation experiments where both strains were cultivated in TSB. The growth curves were determined by measuring the optical density and the CFU counts were quantified after 24 h of cultivation on blood agar plates.

Interspecies interactions involve a wide variety of molecular mechanisms to compete with other microbes, including both contact-dependent and contact-independent mechanisms [40]. To understand whether the microbial interactions between *Ab* and *Sa* are driven by physical cell–cell contacts or purely by secreted diffusible signals, we performed co-cultivation experiments as well as experiments in which both strains were exposed to the 100% of sterile-filtered supernatants of ONCs (CFCM) of the other microbe (Fig. 1). In order to quantify the experimentally obtained growth curves and to compare the various strain combinations in the different experimental settings, we developed an extended logistic growth model based on ODE with parameters that were calibrated to the measured growth curves.

The results section is structured as follows: first, we investigated general interaction principles using laboratory strains—two different strains for each species. For this, we performed and quantified CFU counts as well as growth curves in mono- and co-culture of the different strain combinations and analyzed differentially expressed genes in the CFCM of the other species by RNAseq. To further elucidate the influence of secreted compounds as well as quorum sensing on the *Ab*/*Sa* interaction, we performed experiments with treated CFCM as well as different *Sa* knock-out mutants. Second, in order to also account for the biological variation observed in the clinical setting, we compared CFU counts as well as growth curves of mono- and co-culture experiments of the laboratory strains with seven clinical isolates, where *Ab* and *Sa* occurred in the same infection site.

### *Ab*/*Sa* growth is reduced in CFCM

First, we investigated whether both pathogens interact through secreted compounds present in the CFCM of the respective other strain. Therefore, we used well-known laboratory strains (*Ab* A42 and A118 and *Sa* LS1 and USA300) (Supplementary Table 1) and prepared CFCM from the ONC of *Ab* or *Sa* after 17 h of cultivation. Subsequently, *Ab* or *Sa* from each pair was grown in the CFCM obtained from the other pathogen as well as in control media (TSB) (Fig. 1).

**Figure 2:**
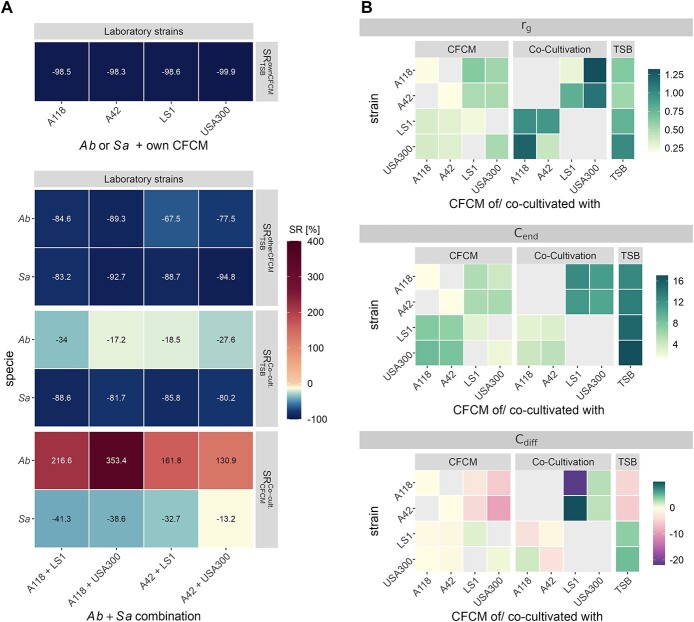
(**A**) Survival ratio $SR$ of the CFU counts after 24 h. Heatmap shows the median values. Raw data and boxplots are presented in Supplementary Fig. 3. The first row shows the $S{R}_{TSB}^{ownCFCM}(s)$ of strain $s$, which is given by ${SR}_{TSB}^{CF CM}(s)=\frac{CF{U}_s^{ownCFCM}- CF{U}_s^{TSB}}{CF{U}_s^{TSB}}$, with the CFU count in its own CFCM ($CF{U}_s^{ownCFCM}$) and its CFU count in the TSB control experiment ($CF{U}_s^{TSB}$). The second row shows the survival ratio $S{R}_{TSB}^{otherCFCM}(s)$ of strain $s$, which is given by ${SR}_{TSB}^{otherCFCM}(s)=\frac{CF{U}_{s+o}^{otherCFCM}- CF{U}_s^{TSB}}{CF{U}_s^{TSB}}$, with the CFU count in the CFCM of the other strain $o$ ($CF{U}_{s+o}^{otherCFCM}$) and its CFU count in the TSB control experiment ($CF{U}_s^{TSB}$). The third row shows the survival ratio $S{R}_{TSB}^{Co- cult.}(s)$ of strain $s$, which is given by ${SR}_{TSB}^{Co- cult.}(s)=\frac{CF{U}_{s+o}^{Co- cult.}- CF{U}_s^{TSB}}{CF{U}_s^{TSB}}$, with the CFU count in co-cultivation with the other strain $o$ ($CF{U}_{s+o}^{Co- cult.}$) and its CFU count in the TSB control experiment ($CF{U}_s^{TSB}$). The fourth row shows the survival ratio $S{R}_{otherCFCM}^{Co- cult.}(s)$ of strain $s$, which is given by ${SR}_{other\ CFCM}^{Co- cult.}(s)=\frac{CF{U}_{s+o}^{Co- cult.}- CF{U}_{s+o}^{other CFCM}}{CF{U}_{s+o}^{other CFCM}}$, with the CFU count in co-cultivation with the other strain $o$ ($CF{U}_{s+o}^{Co- cult.}$) and its CFU count in the TSB control experiment ($CF{U}_s^{TSB}$). (**B**) Estimated model parameters for the growth rate ${r}_g$ at the end value of the carrying capacity and the value ${C}_{diff}={C}_{end}-{C}_{start}$, which shows whether the carrying capacity increases or decreases over the time course of the experiment. Values refer to the median values over all samples. Estimated parameters for individual samples are shown in Supplementary Fig. 6.

After 24 h of incubation, colony forming units (CFU) were counted (Supplementary Fig. 2) and the survival ratio $SR$ of strain $s$ was quantified by calculating ${SR}_{m_{ref}}^m(s)=\frac{CF{U}_s^m- CF{U}_s^{m_{ref}}}{CF{U}_s^{m_{ref}}}$, for medium $m=\left(\mathrm{ownCFCM},\mathrm{otherCFCM}\right)$ and reference medium ${m}_{ref}=\mathrm{TSB}$. $CF{U}_s^m$ refers to the CFU count of strain $s$ in its own CFCM or the CFCM of the other strain, respectively. $CF{U}_s^{m_{ref}}$ refers to the CFU count of strain $s$ in TSB (Fig. 2A and Supplementary Fig. 3; ${SR}_{TSB}^{ownCFCM},{SR}_{TSB}^{otherCFCM}$).

As shown in Fig. 2A, the growth of each species in their own CFCM compared with their growth in TSB is reduced by >98%, indicating that all nutrients have been consumed during the ONC from which the CFCM was derived. Furthermore, the growth of both species is reduced by ~67.5–94.8% in the CFCM of the respective other specie compared with their growth in TSB suggesting competition for the same nutrient sources or impaired growth due to compounds secreted by the other strain (Fig. 2A).

To quantify not only CFU after 24 h but also the time-resolved growth kinetics of the *Ab*/*Sa* interaction dynamics over time, we developed and fitted an extended logistic growth model based on ODE to the measured OD kinetics from the CFCM experiments (see *Methods*). Briefly, we developed an extended logistic growth model based on ODE with a time-dependent carrying capacity, which defines the capability of the medium to maintain a certain population size changes over time due to factors like the availability of nutrients or the secretion of certain toxic compounds. The ODE for a bacterial cell population $B$ is given by $\frac{d{B}_{s=\left\{ AB, SA\right\}}}{dt}={r}_g^S\cdot{B}_s\cdot \left(1-\frac{B_s}{C_s(t)}\right)$ with the growth rate ${r}_g$ and the time-dependent carrying capacity $C(t)$. The dynamic capacity $C(t)$ is given by $C(t)=C(t)=\frac{C_{end}+{C}_{start}}{2}+\frac{C_{end}-{C}_{start}}{2}\cdot \tanh \left(g\cdot \left(t-p\right)\right),$ with ${C}_{start}$ being the start value and ${C}_{end}$ being the end value of the carrying capacity that can increase or decrease over time with a turning point at time point $p$ and gradient $g$ at this time point. Experimental growth curves are shown in Supplementary Fig. 4 together with the fitted simulation kinetics based on the estimated model parameters that are shown in Fig. 2B and Supplementary Fig. 5. It can be seen for laboratory strains in CFCM that the fitted parameters seem to be species-dependent and only small variations are found for the specific strains. Bacterial growth is best described by the growth rate ${r}_g$, the value of the carrying capacity after 24 h, ${C}_{end}$, and the combined value ${C}_{diff}={C}_{end}-{C}_{start}$, which describes whether the carrying capacity decreased (${C}_{diff}<0$) or increased (${C}_{diff}>0$) over the time of the experiment.

As shown in Fig. 2B, for the control experiments in TSB we find high growth rates for both species as well as high values for the end value of the carrying capacity with slightly higher values for *Sa*. This is expected since TSB provides a nutrient rich environment and, thus, optimal growth conditions. However, while we observed an increase in the carrying capacity over time for *Sa*, it is decreasing over time for *Ab*, indicating differences in nutrient utilization or secretion dynamics between the two species. Furthermore, growth in their own CFCM yields for both species very low growth rates as well as end values for the carrying capacity, which is also not changing over time. This is can be explained by the fact that all nutrients have already been consumed during the ONC. For the growth in the CFCM of the other strain, we find for *Ab* growth rates only slightly lower than in TSB, while, for *Sa*, growth rates are strongly reduced compared with its growth in TSB. However, the end value of the carrying capacity is higher for *Sa* compared with *Ab*, although not as high as in TSB. While the carrying capacity remains constant for *Sa,* it decreases for *Ab*.

Therefore, estimated model parameters based on the time-resolved growth curves are in line with the measured survival ratios based on CFU counts: For both species, almost no growth is possible in their own CFCM. However, for both species, the growth in the CFCM of the other species is possible to a certain extent. This suggests that *Ab* and *Sa* share only some of the nutrients. Furthermore, the estimated growth rates indicate that the growth of *Ab* is not directly impaired by *Sa* secreted compounds but *Ab* either rapidly consumes all remaining nutrients or decreases the capacity of the medium by secreting toxic compounds. On the contrary, the growth of *Sa* seems to be impaired by secreted compounds of *Ab*, while the medium still provides sufficient nutrients to allow growth to a certain extent.

### Competition via physical contact promotes *Ab* but not *Sa* growth

Next, we investigated the impact of physical cell–cell contacts on the growth of both species in co-cultivation experiments. Therefore, all bacterial cultures were adjusted to the same initial number of bacteria and the growth was analyzed after 24 h of incubation by CFU counting for each pathogen in both mono and mixed cultures. These values were used to calculate the survival ratio $SR$ in co-cultivation with respect to the growth in TSB (Fig. 2A and Supplementary Fig. 3; ${SR}_{TSB}^{Co- cult.}$). For *Sa*, the survival in co-cultivation is similarly strongly reduced as in CFCM from *Ab*, while the survival of *Ab* in co-cultivation with *Sa* is only reduced by 17.2–34% compared with its growth in TSB. While CFU counts of *Ab* were increased by 130.9–353.4% in co-cultivation compared with its growth in CFCM from *Sa*, the growth of *Sa* was reduced by 13.2–41.3% in co-cultivation compared with its growth in CFCM from *Ab* (Fig. 2A; $S{R}_{otherCFCM}^{Co- cult.}$). Furthermore, as can be seen for the laboratory strains in Supplementary Fig. 1, *Ab* is dominating the growth in co-cultivation after 24 h with fractions of ~75% of the total CFU.

To simulate bacterial growth in co-culture, the model described above was simulated for both species. Since in the experiment only total growth can be measured during co-culture we fitted the combined population sizes to the measured OD kinetics (see Methods). Thus, the model does not only allow quantifying the overall growth in co-culture but also provides predictions on the kinetics of the two subpopulations, which are not accessible through the experiments. Measured growth curves and fitted model dynamics are shown in Supplementary Fig. 4. Estimated model parameters are shown in Fig. 2B and Supplementary Fig. 5. As can be seen, growth rates show higher variability than in CFCM experiments but were in general predicted to be higher for both species compared with growth in the CFCM of the other species. This could be indicative for a cooperative relationship due to physical contacting. In contrast to the growth in the CFCM of the other species we predict that in co-cultivation the end value of the carrying capacity is much higher for *Ab* than for *Sa* and also that the trend of the carrying capacity over the incubation time of the experiment is inverted. Thus, we can conclude that in co-cultivation, the growth rate is more strain-dependent. However, although both species have, in general, similar growth rates, *Ab* can outgrow *Sa* for all strains in a similar way.

These results indicate that *Ab* benefits from physical contact with *Sa*, possibly by accessing shared nutrients more effectively, while Sa is negatively influenced by direct contact with *Ab*, suggesting potential interference or competition mechanisms.

### Cooperative interaction is driven by the alternative carbon source acetoin

Growth reduction in CFCM (Fig. 2A) can be explained by the production of toxins (interference competition) or by depletion of nutrients (exploitative competition). Since toxins can only partly explain growth reduction in CFCM, we analyzed individual transcriptional profiles in our *in vitro* setting by RNAseq of *Sa* USA300 in the CFCM of *Ab* A118 and *vice versa* after 3 h of growth and compared them to their gene expression in fresh media. This provides valuable insights into the influence of both nutrient availability and secreted molecules on *Ab* and *Sa* growth and gene expression. Significantly up- and downregulated genes are presented as volcano plots in Supplementary Fig. 6.

Differential expression analysis of a total of 3400 genes in *Ab* revealed that 587 were significantly regulated (*P* < 0.05; logFC > *1*) upon exposure to CFCM of *Sa* (Supplementary Fig. 6A; Supplementary Table 3). *Ab* showed a higher number of significantly upregulated genes mainly related to the degradation of carbohydrates such as acetoin, aminoacids, quorum sensing like the signal molecules N-homoserine lactones and lipids, while than downregulated genes were related to stress factors and carbohydrates biosynthesis. However, most of the up- or downregulated genes could not be associated to any particular pathway (hypothetical or missing annotation). Downregulated genes with known annotations were associated with lipoproteins, transport reactions, and stress proteins. Upregulated genes in this list with annotation were associated with bacterial regulation, acetoin degradation and benzoate reactions. Significantly regulated genes were also submitted to Uniprot for pathway, which showed that these genes were mostly mapped to amino acid degradation, carbohydrate metabolism and cofactor biosynthesis (Supplementary Table 4).

Differential expression analysis of a total of 2560 genes in *Sa* revealed that 541 were significantly regulated (*P* < 0.05; logFC >1) upon culture in CFCM of *Ab* (Supplementary Fig. 6B; Supplementary Table 5). As can be seen in the volcano plot displayed in Supplementary Fig. 6B, several genes showed significant downregulation related to toxins such as phenol soluble modulins (PSMs), capsule, ABC transporters and quorum sensing. Highly significant and upregulated genes were related to the biosynthesis of aminoacids, carbohydrates and special compounds such as aceptoin.. Other genes found in this group were associated with membrane protein and receptor systems, ATP synthase and acetyl-CoA carboxylase. Significantly regulated genes were submitted to Uniprot for pathway analysis, which showed that they were mostly mapped to amino acid biosynthesis, carbohydrate metabolism, purine metabolism, co-factor biosynthesis, and carbohydrate degradation (Supplementary Table 6). Interestingly, among the upregulated *Sa* genes are several genes involved in acetoin biosynthesis (Supplementary Table 6; Supplementary Figs 6B and 7).

**Figure 3:**
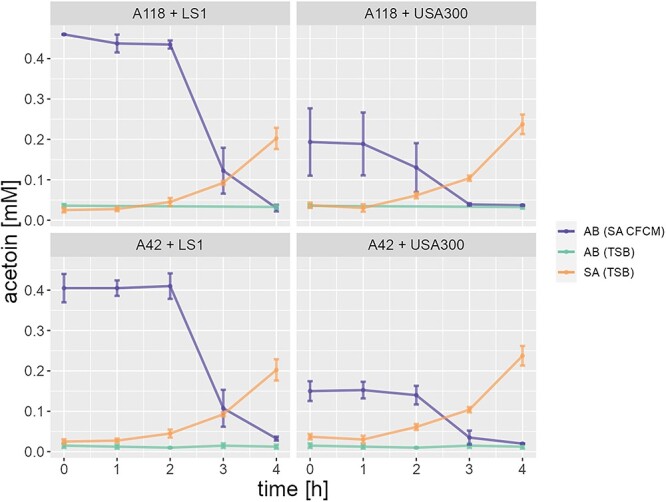
Acetoin dynamics for laboratory strains. The production of acetoin for each strain in TSB after 4 h of incubation is shown for *Ab* strains (AB (TSB)) and for *Sa* strains (SA (TSB)). Furthermore, the consumption of acetoin by the *Ab* strains incubated in the CFCM of the *Sa* strains is shown (AB (SA CFCM)). The final concentration of acetoin is shown in Supplementary Fig. 8.

Based on the differential expression analysis, we hypothesized that the observed cooperative interaction might be driven by the alternative carbon source acetoin (Supplementary Tables 3–6). To investigate this hypothesis, the concentration of acetoin was measured in the CFCMs of *Sa* USA300 and *Ab* A118. In agreement with the literature [3, 41, 42], we found no acetoin in the CFCM of *Ab,* while it accumulated in the CFCM of *Sa* (Supplementary Fig. 8). Next, we monitored the concentration of acetoin during the cultivation of *Ab* A118 and A42 in CFCM of *Sa* LS1 and USA300 (Fig. 3). A decrease of acetoin was observed during the first 4 h of incubation of *Ab* in CFCM of *Sa*, suggesting the consumption of acetoin by *Ab*. Taken together, our results show that *Sa* and *Ab* may have a cooperative interaction due to the use of acetoin.

### *Ab*/*Sa* partially compete via quorum sensing

To identify the nature of molecules in the CFCM from both species (*Sa* USA300 and *Ab* A118) that interfere with the growth of the other species, we pre-treated the CFCM with heat, trypsin or proteinase K. We chose these treatments to target different classes of molecules present in the CFCM. Heat treatment was chosen to denature proteins, while trypsin and proteinase K were chosen to degrade proteins and peptides, respectively. By subjecting the CFCM to these treatments, we aimed to elucidate the role of proteinaceous molecules in mediating the observed growth interference between *Sa* and *Ab*.

**Figure 4:**
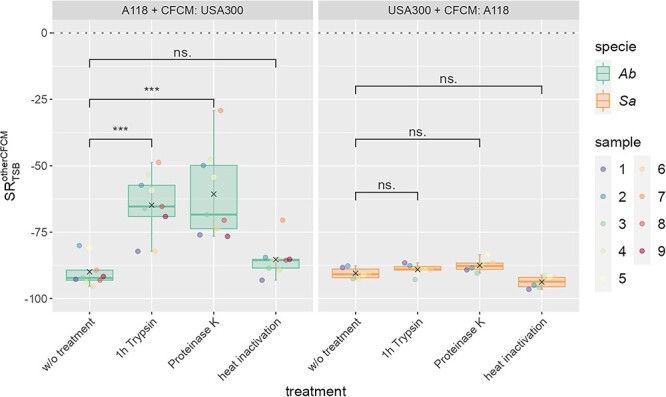
Survival ratio $S{R}_{TSB}^{otherCFCM}$. CFCM was either untreated or treated with trypsin, proteinase K, or heat inactivated. $S{R}_{TSB}^{otherCFCM}$ of *Ab* A118 in CFCM of *Sa* USA300 (left) and growth of *Sa* USA300 in CFCM of *Ab* A118 (right). Points refer to individual samples. Gray crosses denote the mean value and colored boxes denote the interquartile range (IQR) between the 25 and 75% quantiles, with the median shown as a horizontal line. Whiskers show the interval between the $75\%\mathrm{quantile}+1.5\cdot \mathrm{IQR}$ and the $25\%\mathrm{quantile}-1.5\cdot \mathrm{IQR}$. Significance was tested using a paired *t*-test and false discovery rate was considered using the Benjamini–Hochberg correction (significance levels: ns: *P* > 0.05, ^*^P < 0.05; ^*^^*^P < 0.01; ^*^^*^^*^P < 0.001; ^*^^*^^*^^*^P < 0.0001.) for the effect size Cohen’s *d* was calculated as given in Supplementary Table 7.

After cultivation of *Sa* or *Ab* in the pre-treated CFCM, we counted the CFU (Supplementary Fig. 9) and calculated the survival ratio ${SR}_{TSB}^{otherCFCM}$ (Fig. 4). We tested for significant changes of the survival ratios in treated CFCM in comparison to untreated CFCM and calculated the effect size (see Supplementary Table 7). We found that the survival ratio was significantly higher (~25%) for *Ab* A118 when it was cultivated in the CFCM from *Sa* pre-treated with trypsin or proteinase K compared with growth in CFCM without any treatment (Fig. 4). However, pre-heat inactivation of CFCM of *Sa* showed no effect on the growth of *Ab* A118. This suggests that *Sa* interferes with the growth of *Ab* by a heat-resistant protein molecule. Among *Sa* proteins, PSMs are heat-resistant small cytolytic peptides regulated by the accessory gene regulator (Agr) [43, 44, 45, 46, 47, 48]. In contrast, no differences were observed regarding the survival of *Sa* USA300 in CFCM of *Ab* pre- or not treated (Fig. 4). This indicates that a non-protein and heat-resistant secreted compound from *Ab* affects *Sa* growth such as N-acyl homoserine lactones, the quorum sensing signals expressed by *Ab*. These molecules have already been studied by John et al. as a major antibacterial factor produced by *Ab* against *Sa* [49]. Furthermore, a strong upregulation of N-acyl homoserine lactones was found in our RNA seq of A118 in the CFCM of USA300 (Supplementary Table 3, *Ab* in *Sa* CFCM).

Furthermore, RNAseq showed that *agr* and *psm* genes were downregulated in *Sa* USA300 in the CFCM of *Ab* A118 suggesting a potential modulation of quorum sensing and PSMs production in response to the presence of *Ab* A118. Several studies found that *Sa* interacts through quorum sensing with other microbes such as *P. aeruginosa*, *Corynebacterium spp.*, and *Candida albicans* [50, 51, 52, 53, 54]. Therefore, we hypothesize that *Sa* quorum sensing is also involved in the *Ab/Sa* interaction. DNA microarrays showed that *Sa* LS1 and USA300 belong to the CC8 clonal complex (Supplementary Table 8) and the functionality of Agr was investigated using the CAMP test, which was positive for both strains (Supplementary Fig. 10). We also performed a proteomic analysis of the CFCM of *Sa* USA300 and *Ab* A118 to identify what compounds are secreted by each strain into the medium individually. This revealed that *Sa* significantly secretes PSMs (Supplementary Table 9).

**Figure 5:**
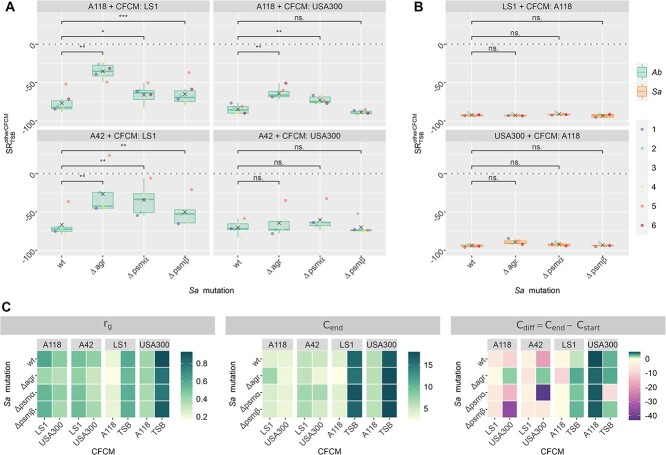
(**A**) Survival ratio $S{R}_{TSB}^{otherCFCM}$ of *Ab* A118 and A42 in CFCM from *Sa* LS1 and USA300 wt, *Δagr, Δpsmα*, and *Δpsmβ* mutants. (**B**) Survival ratio $S{R}_{TSB}^{otherCFCM}$ of *Sa* LS1 and USA300 wt and *Δagr* mutant in *Ab* A118 CFCM. Points refer to individual samples. Gray crosses denote the mean value and colored boxes denote the IQR between the 25 and 75% quantiles, with the median shown as a horizontal line. Whiskers show the interval between the $75\%\mathrm{quantile}+1.5\cdot \mathrm{IQR}$ and the $25\%\mathrm{quantile}-1.5\cdot \mathrm{IQR}$. Significance was tested using a paired *t*-test and false discovery rate was considered using the Benjamini–Hochberg correction (significance levels: ns: *P* > 0.05, ^*^P < 0.05; ^*^^*^P < 0.01; ^*^^*^^*^P < 0.001; ^*^^*^^*^^*^P < 0.0001.) for the effect size Cohen’s d was calculated as given in Supplementary Table 10. (**C**) Estimated model parameters for growth curves of *Ab* A118 and A42 in CFCM from *Sa* USA300 and LS1 as well as the CFCM from their derivate mutants *∆agr, ∆psmα*, and *∆psmβ* and *vice versa.* The growth rate ${r}_g$ the end value of the carrying capacity and the value ${C}_{diff}={C}_{end}-{C}_{start}$, which shows whether the carrying capacity increases or decreases over the time course of the experiment. Colors refer to the median values over all samples. Estimated parameters for individual samples are shown in Supplementary Fig. 14.

Next, we investigated the effect of *Sa* Agr and PSMs on *Ab*. Thus, *Ab* A118 and A42 were exposed to the CFCM from *Sa* USA300 and LS1 as well as the CFCM from their derivate mutants ∆*agr*, ∆*psmα*, and ∆*psmβ*. After 24 h of incubation, we counted CFU (Supplementary Fig. 11) and calculated the survival ratio ${SR}_{TSB}^{otherCFCM}$ (Fig. 5A). We tested for significant changes of the survival ratios in comparison to *Sa* wt and calculated the effect size (see Supplementary Table 10). We observed a significant increase in the survival ratio when *Ab* was exposed to the CFCM of *Sa* LS1 ∆*agr,* ∆*psmα*, and ∆*psmβ* compared with the wild-type (wt) strain. Similarly, A118 showed increased survival ratios when it was exposed to the CFCM of USA300 ∆*agr* and ∆*psmα* but not *∆psmβ.* However, no significant difference was found when A42 was exposed to the CFCM of USA300 and its mutants. Interestingly, the survival ratio of A118 and A42 was 30–47% higher in the CFCM of LS1 ∆*agr* and ~20% higher in the CFCM of LS1 ∆*psmα* compared with the CFCM of LS1 wt. However, these differences were less pronounced when A118 was incubated in the CFCM of USA300 wt and its mutants and imperceptible for A42 (Fig. 5A). Our results suggest that the growth limiting effect of the staphylococcal PSM*s* on *Ab* is strain-dependent.

Next, we analyzed whether *Ab* can target the Agr system from *Sa* (Fig. 5B). Therefore, we incubated *Sa* USA300 and LS1 wt and their corresponding mutants in the CFCM of *Ab* A118 and calculated the survival ratio $S{R}_{TSB}^{otherCFCM}$ (Fig. 5B). The survival ratio was not significantly higher for any of the *Sa* mutant strains showing that they were unable to overcome the effect of CFCM of *Ab* A118. Even though slightly higher survival ratios were obtained with both *Sa* ∆*agr* strains compared with wt strains, these differences were not significant. Likewise, we investigated possible toxins from *Ab* that might interfere with the growth of *Sa*. Proteomic analysis of CFCM of *Ab* revealed the enrichment of type VI (T6SS) and type I (T1SS) secretion systems (Supplementary Table 9). The role of secretion systems of Gram-negative bacteria during the interaction with other bacteria and, in particular, the killing of *Sa* by *Ab* through T6SS was recently described [55, 56]. Therefore, we amplified a gene encoding T6SS in both *Ab* strains and could confirm its presence by PCR (Supplementary Fig. 12). This could explain the contact-dependent inhibition of *Sa* by *Ab* that we observed during co-cultivation experiments (Fig. 2; Supplementary Fig. 5).

Furthermore, we obtained growth curves of *Ab* A118 and A42 in the CFCM from *Sa* USA300 and LS1 wt and their corresponding mutants and *vice versa*. We calibrated the mathematical model to these data and the simulated growth curves are in good agreement with the experimental growth curves as shown in Supplementary Fig. 13. The estimated model parameters are presented in Fig. 5C and Supplementary Fig. 14. The growth rates are reduced for both *Ab* in the *Sa ∆agr* mutant as well as of the *Sa ∆agr* mutant in CFCM from *Ab.* However, ${C}_{end}$ values only slightly differ for the different *Sa* mutants and ${C}_{diff}$ values show a heterogeneous pattern. In summary, while end values of the carrying capacity are similar for *Ab* in CFCM from *Sa* derivate mutants and *vice versa*, growth dynamics are slower for *Ab* in CFCM from *Sa ∆agr*, although viability is increased.

These findings highlight a complex interplay between *Sa* and *Ab*, where the production of PSMs by *Sa* and the expression of T6SS by *Ab* contribute to a partial inhibition of each other's growth.

### Clinical isolates exhibit heterogeneous interaction patterns in co-cultivation

Since the laboratory strains do not naturally occur in coexistence, we aimed to gain a more comprehensive understanding of the biological variability observed in clinical settings. Therefore, we also conducted CFCM and co-cultivation experiments using multiple *Ab*/*Sa* pairs that were co-isolated from hospitalized patients (Supplementary Table 2).

**Figure 6:**
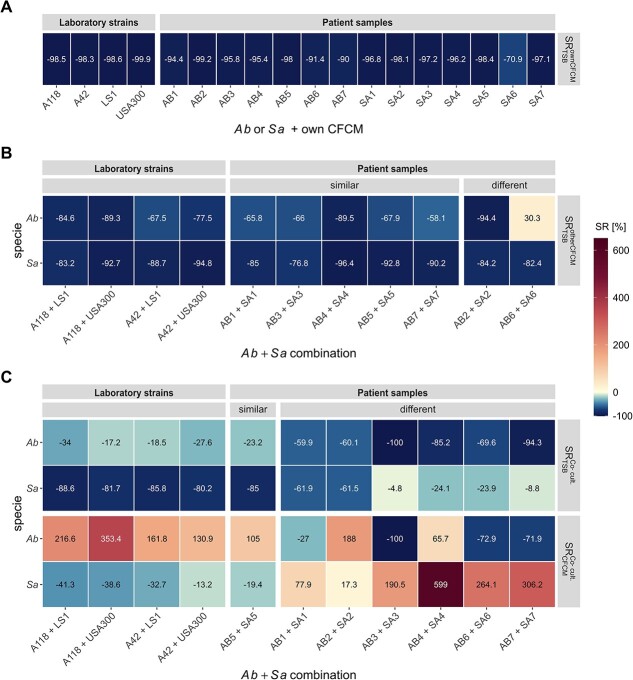
Survival ratio $SR$ of the CFU counts after 24 h. Heatmap shows the median values. Raw data and boxplots are presented in Supplementary Fig. 3. (**A**) Survival ratio $S{R}_{TSB}^{ownCFCM}(s)$ of strain $s$, which is given by ${SR}_{TSB}^{CF CM}(s)=\frac{CF{U}_s^{ownCFCM}- CF{U}_s^{TSB}}{CF{U}_s^{TSB}}$, with the CFU count in its own CFCM ($CF{U}_s^{ownCFCM}$) and its CFU count in the TSB control experiment ($CF{U}_s^{TSB}$). (**B**) Survival ratio $S{R}_{TSB}^{otherCFCM}(s)$ of strain $s$, which is given by ${SR}_{TSB}^{otherCFCM}(s)=\frac{CF{U}_{s+o}^{otherCFCM}- CF{U}_s^{TSB}}{CF{U}_s^{TSB}}$, with the CFU count in the CFCM of the other strain $o$ ($CF{U}_{s+o}^{otherCFCM}$) and its CFU count in the TSB control experiment ($CF{U}_s^{TSB}$). (**C**) Survival ratio $S{R}_{TSB}^{Co- cult.}(s)$ of strain $s$, which is given by ${SR}_{TSB}^{Co- cult.}(s)=\frac{CF{U}_{s+o}^{Co- cult.}- CF{U}_s^{TSB}}{CF{U}_s^{TSB}}$, with the CFU count in co-cultivation with the other strain $o$ ($CF{U}_{s+o}^{Co- cult.}$) and its CFU count in the TSB control experiment ($CF{U}_s^{TSB}$). The fourth row shows the survival ratio $S{R}_{otherCFCM}^{Co- cult.}(s)$ of strain $s$, which is given by ${SR}_{other\ CFCM}^{Co- cult.}(s)=\frac{CF{U}_{s+o}^{other CFCM}- CF{U}_{s+o}^{other CFCM}}{CF{U}_{s+o}^{other CFCM}}$, with the CFU count in co-cultivation with the other strain $o$ ($CF{U}_{s+o}^{Co- cult.}$) and its CFU count in the TSB control experiment ($CF{U}_s^{TSB}$).

First, we analyzed the survival ratios (Fig. 6). For growth in the CFCM of the other species compared with growth in TSB ($S{R}_{TSB}^{otherCFCM}$), we found that most of the samples follow the trend observed for the laboratory strains (pairs 1, 3, 4, 5, and 7). However, for samples 2 and 6 we observed a different behavior: in pair 2, *Ab* was stronger reduced than *Sa*, while in pair 6, *Ab* had a growth advantage over its growth in TSB. Furthermore, we also compared the growth of each pathogen in co-cultivation with the growth in monoculture in either TSB ($S{R}_{TSB}^{Co- cult.}$) or in the CFCM of the other species ($S{R}_{otherCFCM}^{Co- cult.}$). In contrast to the laboratory strains, a heterogeneous pattern was observed for the patient samples, with only isolate 5 conforming to the trend seen in the laboratory strains. The trend in the survival ratio for clinical isolates 3, 4, 6, and 7 has been reversed, whereby *Ab* demonstrated a more substantial reduction (~85–100%) in contrast to *Sa* (~40%). Meanwhile, in clinical isolates 1 and 2, both species were equally reduced (~60–70%). Interestingly, we observed greater growth in co-culture for the patient samples for almost all *Sa* strains compared with the corresponding CFCM experiment ($S{R}_{otherCFCM}^{Co- cult.}$). The growth of *Ab* was decreased in samples 1, 3, 6, and 7 during co-cultivation compared with the growth in CFCM from *Sa*.

**Figure 7:**
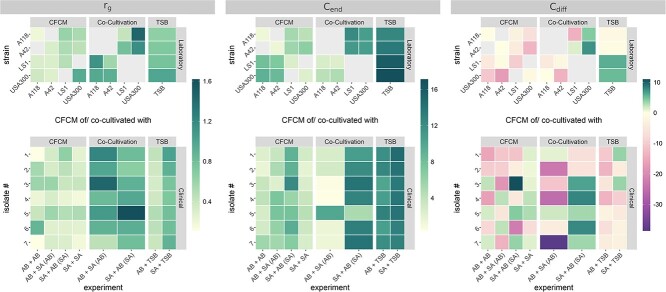
Estimated model parameters. Values refer to the median values over all samples. Estimated parameters for individual samples are shown in Supplementary Fig. 5.

Next, we also fitted the *in silico* model to the growth curves (Supplementary Fig. 15). All estimated model parameters are shown in Supplementary Fig. 16. Median values for the parameters ${r}_g$, ${C}_{end}$, and ${C}_{diff}$ are presented as a heatmap in Fig. 7. As expected for patient samples, we observed larger variations at the level of individual strains. For the growth in TSB, we estimated similar growth rates ${r}_g$ and end values for the carrying capacity ${C}_{end}$ as for the laboratory strains. In line with the laboratory strains, these values are slightly higher for *Sa* compared with those of *Ab*. However, the reduction in the carrying capacity over time that we observed for the laboratory strains of *Ab* was not present for most of the patient samples. Instead, we estimate a slight increase in the carrying capacity for both species.

For the growth in their own CFCM all three model parameters are in line with the laboratory strains. However, for the growth in the CFCM of the other species, the difference in the growth rates ${r}_g$ of the two species is not present for the patient samples, but the growth rate of *Ab* is estimated to be lower than that of the laboratory strains and more similar to that of *Sa*. For the laboratory strains, estimated ${C}_{end}$ values were higher for *Sa* than for *Ab*, which we only observed for clinical pairs 1, 2, 3, and 6, while the opposite was the case for pairs 4 and 5 and similar values for both species were found for sample 7. The trend of the carrying capacity showed also a heterogeneous pattern. In general, we predict an increase in the carrying capacity for all *Sa* strains, although the strength is highly strain-dependent. Furthermore, for *Ab* we predict a decrease in the carrying capacity for isolates 1, 2, and 7, an increase for isolates 4, 5, and 6 and a constant carrying capacity for isolate 3. For the growth in co-cultivation we predict in general high growth rates. However, here we cannot identify a clear pattern that would be in line with the laboratory strains. Interestingly, for the end value of the carrying capacity ${C}_{end}$, we find one clinical isolate (pair 5) that is in line with the laboratory strains, while all other isolates behave contrary, i.e. ${C}_{end}$ is much higher for *Sa* than for *Ab*. For ${C}_{diff}$, estimated values are very heterogeneous, and no clear pattern can be identified, which was also the case for the laboratory strains.

These results indicate that the interaction via secreted compounds is conserved for the majority of patient samples. However, the contact-dependent interaction is highly strain-dependent and might be influenced by the environmental conditions inside the specific infection site.

### *Ab*/*Sa* interaction is not influenced by the type of Agr locus, clonal complex, or T6SS

Both laboratory *Sa* strains belong to the clonal complex CC8 and contain the Agr I locus. To investigate whether the observed differences in *Ab* growth in the presence of *Sa* could be related to the clonal complex and/or the Agr locus, we performed a microarray analysis for all clinical *Sa* isolates (Supplementary Table 8).

Clinical strains belong to six different clonal complexes (CC8, CC25, CC15, CC1, and CC45). The *agr* genotyping also revealed the phenotypic diversity of the strains; where the strains SA1-SA4 contain genes for Agr I and SA5 and SA6 contain the Agr genes II and III, respectively. Furthermore, the functional expression of *agr* was investigated by the CAMP test among all clinical strains (Supplementary Fig. 10). However, there were no observable differences in survival ratios and growth curves for *Ab*/*Sa* interactions across different clonal complexes or Agr loci. Thus, we conclude that the *Ab*/*Sa* interaction is not directly linked to the type of Agr locus or the clonal complex.

Furthermore, proteomics analysis of the CFCM of *Ab* A118 revealed the enrichment of T6SS (Supplementary Table 9). The amplification of T6SS in all clinical *Ab* isolates showed enrichment for AB2, AB3, and AB4, while less copies were found in AB1 and AB4 and none in AB6 and AB7 (Supplementary Fig. 11). However, similar growth reduction of *Sa* was observed in co-cultivation with *Ab* isolates with and without T6SS (Fig. 2, Supplementary Fig. 3). These results suggest that *Ab* may also interfere with *Sa* via other factors in addition to T6SS.

**Figure 8:**
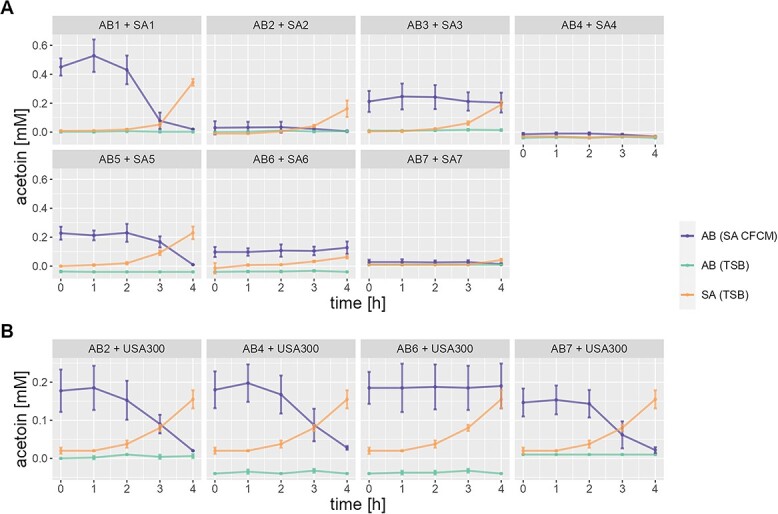
(**A**) Acetoin dynamics in patient samples. The production of acetoin for each strain in TSB after 4 h of incubation is shown for *Ab* strains (AB (TSB)) and for *Sa* strains (SA (TSB)). Furthermore, the consumption of acetoin by the *Ab* strains incubated in the CFCM of the *Sa* strains is shown (AB (SA CFCM). (**B**) The combined measurements represent the consumption of acetoin by *Ab* strains 2, 4, 6, and 7 in the CFCM of *Sa* USA300, which is a high producer of this metabolite. The final concentration of acetoin is shown in Supplementary Fig. 8.

Taking into account that for the laboratory strains we identified a cooperative interaction via the cross-feeding of acetoin, we also measured the acetoin production by the clinical strains after 24 h (Supplementary Fig. 8). As expected, none of the *Ab* strains did produce acetoin. Similar to the laboratory strains, clinical strains SA1, SA2, and SA5 produced high amounts of acetoin, while SA2, SA4, SA6, and SA7 produced only low amounts or no acetoin at all. Next, we measured acetoin consumption by clinical *Ab* strains over 4 h for *Sa* in TSB as well as for *Ab* in TSB and in the 24h CFCM of *Sa* (Fig. 8A). Clinical pairs 1 and 5 are in line with the laboratory strains, while AB3 and AB6 seem not to consume acetoin. For the low/no producer *Sa* strains 2, 4, 6, and 7, it is unclear if their corresponding *Ab* strains effectively consume this metabolite. To test this, these strains were grown in the CFCM of the high-producer *Sa* USA300. As can be seen in Fig. 8B, AB2, AB4, and AB7 indeed consume acetoin, while this was not the case for AB6, which could be due to a mutation in the operon responsible for acetoin metabolism in this strain.

## Discussion

In this study, we investigated the *Ab/Sa* interaction using a comprehensive approach. To capture both general principles and biological variation seen in clinical samples, we deliberately selected two laboratory strains for each species, as well as seven co-isolated *Ab/Sa* pairs from hospitalized patients. Our investigation included a total of 11 *Ab/Sa* pairs, using a mixture of wet-lab experiments and subsequent development of an *in silico* model. This model allowed us to effectively quantify and compare different experimental conditions. Our results revealed a nuanced interplay between the two pathogens, revealing a dynamic mix of competition and cooperation (see Graphical Abstract).

First, we examined the general interaction principles using laboratory strains. We assessed their growth under two different conditions: (i) in the CFCM of the respective other species to account for contact-independent mechanisms and (ii) in co-culture with the other species to evaluate the impact of direct physical contacts. For this, CFU counts were performed after 24 h of incubation and OD measurements were done to obtain growth curves over a time span of 24 h (Fig. 1).

The impact of each pathogen's CFCM on the respective other was evaluated in comparison to pathogen viability in TSB medium. The resulting survival ratios indicated that both *Sa* and *Ab* exhibited reduced growth in the CFCM of the respective other species when compared with monocultures in TSB. Interestingly, the survival ratio from co-cultivation experiments revealed that, while the growth of *Sa* was impaired, *Ab*'s growth was only slightly affected. In fact, when comparing the survival ratios between growth in CFCM and in co-cultivation, *Ab* exhibited higher growth in the co-cultivation setting. To quantitatively characterize this interaction dynamics, we developed an extended logistic growth model with parameters that were calibrated to the measured growth curves. The estimated model parameters are in line with the observations from the survival ratios. Consequently, these results imply that the competition between *Sa* and *Ab* is due to their shared utilization of similar nutrients and due to toxic compound secretion. Furthermore, *Sa's* growth is detrimentally impacted by physical contact with *Ab*.

To investigate components responsible for the observed competition, we pre-treated the CFCM from each pathogen with trypsin, proteinase, or heat before exposure to the respective other pathogen. Our results indicate that the growth of *Ab* was influenced by staphylococcal PSMs. Additionally, PSMs were found to be involved in the interaction between *Sa* and *Corynebacterium pseudodiphtheriticum* [34]. We employed a similar approach to assess the secreted compound from *Ab* that affects the growth of *Sa* and found that this compound was resistant to trypsin, proteases, and heat. Upon consulting the literature, we identified the quorum sensing molecule N-acyl homoserine lactone produced by *Ab* as a promising candidate with the aforementioned attributes. Furthermore, the synthesis of N-homoserine lactones was upregulated in the RNA-Seq of *Ab* 118 in the USA300 CFCM. These results correlate with another study documenting that N-acyl homoserine lactones have antibacterial properties against several pathogens, including *Sa* [49]. Future studies should be conducted to confirm N-acyl homoserine lactone as the quorum sensing molecule produced by *Ab* that interferes with *Sa*.

To identify the specific compounds that are regulated when a pathogen is grown in the CFCM of another species in comparison to fresh medium, we performed RNAseq analyses of USA300 in the CFCM of A118 and *vice versa*. Genes related to quorum sensing and acetoin degradation were upregulated in A118 in CFCM of USA300 [41], which is in line with findings reported by Eckhardt et al [27]., while, among others, genes related to PSMs and quorum sensing were downregulated in *Sa* USA300 in CFCM of A118.

Furthermore, pathways related to acetoin production and certain amino acids were upregulated. This suggests a cooperative interaction via cross-feeding of alternative carbon sources to generate energy. Recent studies reported a similar interaction between *P. aeruginosa* and *Sa*[3] as well as between *K. pneumonia* and *Ab* [5]. Given the well-established competition between pathogens and the host for glucose during infection [57], it is evident that bacterial organisms have developed alternative survival strategies in low-glucose environments. Consequently, interactions between *S. aureus* and other pathogens may involve a cooperative mechanism facilitated by acetoin production, which serves as an alternative carbon source to sustain fermentative bacteria. It follows that different regulatory mechanisms of the acetoin system and possible deficiencies in essential genes associated with acetoin production or metabolism in clinical samples may account for the variability observed in clinical strains. It is known that *Sa* can generate acetoin through the decarboxylation of alpha-acetolactate [3, 42], while *Ab* cannot. However, *Ab* is able to enhance the catabolism pathway of acetoin under stress conditions [41]. In fact, we have shown that acetoin was present in a high concentration in CFCM from *Sa* while CFCM from *Ab* lacked this metabolite. Furthermore, we could show that *Ab* was able to use acetoin in the CFCM from *Sa*. This cooperative interaction could explain the frequent co-occurrence of these pathogens in clinical samples [11, 12, 15, 16]. While the benefit during the *Ab*/*Sa* interaction seems obvious for *Ab*, *Sa* may benefit from the creation of a microenvironment conducive to its survival and persistence. In the presence of *Ab*, a downregulation of virulence factors by *Sa* was observed. We and others have shown that downregulating virulence factors by *Sa* increases its ability to persist [58, 59, 60]. In addition, *Sa* may benefit from metabolic interactions, shared nutrient resources, or modulation of the host immune response. Further investigation is needed to fully elucidate these advantages for *Sa*, which will lead to a better understanding of polymicrobial infections and may have implications for therapeutic strategies.

Subsequently, we conducted a proteomic analysis to examine the protein composition in each CFCM. Interestingly, one of the proteins found to be upregulated in *Sa* CFCM were PSMs. In contrast, the *Ab* CFCM revealed a high enrichment of transporter system VI and I (T6SS and T1SS, respectively). Several studies have described the killing of Gram-positive bacteria by T6SS [55, 61]. PCR analysis revealed the absence of T6SS in two *Ab* strains. Since the N-acyl homoserine lactones are not proteins, they were not found by proteomics.

To further investigate the role of PSMs in the growth of *Ab*, we exposed *Ab* to CFCM from *Sa* ∆*agr*, ∆*psmα*, and ∆*psmβ* mutant strains. *Ab* viability was notably higher in CFCM from the mutant strains compared with the wt strain. However, these differences were not statistically significant when we exposed the multidrug resistant *Ab* A42 to USA300. Additionally, we exposed *Sa* ∆*agr*, ∆*psmα*, and ∆*psmβ* mutant strains to CFCM from *Ab*. Surprisingly, we found no significant differences between the wt and mutant strains in their response. The *in silico* model predicts lower growth rates for both, *Ab* in *Sa* ∆*agr* CFCM as well as *Sa* ∆*agr* in *Ab* CFCM, while the growth curves reach similar values after 24 h (Supplementary Fig. 13). This shows that bacterial growth might be slower, but the medium is able to maintain cell viability due to an absence of toxins or due to their eventual inactivation, and due to an absence of changes to the pH. Therefore, quorum sensing partly contributes to the competition between *Ab* and *Sa*.

To explore biological variations in the *Ab*/*Sa* interaction when they naturally occur together, we conducted a detailed analysis of seven clinical samples. We observed a heterogeneous picture where some pairs followed the trends observed for the laboratory strains, while others did not. During cultivation in the CFCM of the other strain survival ratios were in line with the laboratory strains for most clinical isolates, this suggests that interaction via secreted compounds is conserved among the strains. In contrast, interaction patterns in co-cultivation experiments showed much higher variability. These findings indicated higher strain-dependency related to the expression of different virulence factors such as PSMs, N-homoserin lactones, and secretion system VI among clinical samples. Furthermore, also the cooperation via cross-feeding of acetoin underlies higher strain-dependency. While none of the *Ab* strains produced acetoin, some *Sa* strains were high producers, similar to the laboratory strains, and others were only low- or even no-producers. Among other factors this could be a reason that for several clinical isolates (3, 4, 6, 7) the trend in the survival ratios was reversed in comparison to the laboratory strains (see Fig. 6C; $S{R}_{TSB}^{Co- cult.}$). For these clinical isolates, either *Ab* was a non-consumer or *Sa* was a non-producer of acetoin, thus, depriving *Ab* of the benefit of cross-feeding during the interaction. These results suggest that acetoin is a major player in the *Ab/Sa* interaction network, but is not essential for the two pathogens to co-exist.

In the current study, *Ab*/*Sa* interaction was investigated under planktonic conditions. In order to further shed light on the complex interaction between *Ab* and *Sa*, future experiments should account for their interaction in biofilms, since in wound infections they typically form biofilms. There, the spatial structure as well as the extracellular matrix might also affect gene regulation [62]. For example, due to much more physical interactions of both species within a biofilm the T6SS from *Ab* could have a much more pronounced negative effect on *Sa* growth. In addition, the activation of quorum sensing is expected to be enhanced in both *Ab* and *Sa*, resulting in the upregulation of certain toxins. Consequently, the increased expression of these toxins by each pathogen could profoundly affect the competitive fitness of the other within the biofilm environment [23].

Furthermore, the interaction with host cells is an important factor to consider in future studies as well as the interaction of both species *in vivo,* which increases complexity even more. Li et al. performed an *in vivo* study on *Sa* co-infections and could show that co-infections with *Ab* dramatically altered the fitness requirements of *Sa in vivo* [18]. They performed mono- and co-infections of one pair of laboratory strains in mouse models and hypothesized that a direct interaction between *Sa* and *Ab* might shape this altered gene expression. Therefore, they acknowledge the need for further *in vitro* studies to assess this direct interaction [18]. Furthermore, a validation of possible interactions in clinical strains was not done in this study.

In conclusion, in this study we investigated the complex interplay between *Ab* and *Sa*. Here, we have not only conducted numerous experiments but also effectively constructed an *in silico* model to simulate and quantify these interactions across various laboratory and clinical strains of both pathogens. Our results reveal critical pathways for potential therapeutic targets and alternative strategies in combatting *Ab/Sa* infections. The new targets proposed in our study are primarily the PSMs and the production of acetoin by *Sa*. These molecules have been implicated in bacterial virulence and interspecies interactions, making them potential targets for therapeutic intervention. In addition, our results indicate that the system responsible for the catabolism of acetoin is critical in *Ab*, suggesting a potential vulnerability in the pathogen that could be exploited for therapeutic purposes. Furthermore, our results suggest that homoserine lactones produced by *Ab* may play an important role in modulating interspecies interactions. While further investigation will have to fully elucidate the significance of these molecules, our experiments with the treatment of *Ab* CFCM provide preliminary evidence of their potential importance in the context of *Ab*/*Sa* co-infections. These findings pave the way for innovative strategies to combat *Ab*/*Sa* infections, emphasizing the importance of considering these factors in future therapeutic interventions.

## Supplementary Material

2024-06-20_Supplementary_Figures_ycae077

Table_S1_strains_ycae077

Table_S2_model_parameter_estimation_ranges_ycae077

Table_S3_RNAseq_Ab_in_USA300_CFCM_ycae077

Table_S4_Main_RNAseq_pathways_Ab_04_04_2024_ycae077

Table_S5_RNAseq_Sa_in_A118_CFCM_ycae077

Table_S6_Main_RNAseq_pathways_Sa_04_04_2024_ycae077

Table_S7_statistics_for_Figure_4_ycae077

Table_S8_microarray_staphylococcal_strains_ycae077

Table_S9_proteomics_significant_USA300_and_A118_ycae077

Table_S10_statistics_for_Figure_5_ycae077

Suppl_Mat_Proteomics_ycae077

## References

[1] Murray JL , Connell JL, Stacy A et al. Mechanisms of synergy in polymicrobial infections. J Microbio*l* 2014;52:188–99. 10.1007/s12275-014-4067-3 24585050 PMC7090983

[2] Peters BM , Jabra-Rizk MA, O'May GA et al. Polymicrobial interactions: impact on pathogenesis and human disease. Clin Microbiol Re*v* 2012;25:193–213. 10.1128/CMR.00013-11 22232376 PMC3255964

[3] Camus L , Briaud P, Bastien S et al. Trophic cooperation promotes bacterial survival of *Staphylococcus aureus* and *Pseudomonas aeruginosa*. ISME J 2020;14:3093–105. 10.1038/s41396-020-00741-9 32814867 PMC7784975

[4] Tanner WD , Atkinson RM, Goel RK et al. Horizontal transfer of the blaNDM-1 gene to *Pseudomonas aeruginosa* and *Acinetobacter baumannii* in biofilms. FEMS Microbiol Let*t* 2017;364:1–4. 10.1093/femsle/fnx048 28333234

[5] Semenec L , Cain AK, Dawson CJ et al. Cross-protection and cross-feeding between *Klebsiella pneumoniae* and *Acinetobacter baumannii* promotes their co-existence. Nat Commu*n* 2023;14:702. 10.1038/s41467-023-36252-2 36759602 PMC9911699

[6] Tuchscherr L , Bischoff M, Lattar SM et al. Sigma factor SigB is crucial to mediate *Staphylococcus aureus* adaptation during chronic infections. PLoS Patho*g* 2015;11:e1004870. 10.1371/journal.ppat.1004870 25923704 PMC4414502

[7] Nguyen AT , Oglesby-Sherrouse AG. Interactions between *Pseudomonas aeruginosa* and *Staphylococcus aureus* during co-cultivations and polymicrobial infections. Appl Microbiol Biotechno*l* 2016;100:6141–8. 10.1007/s00253-016-7596-3 27236810 PMC4916000

[8] Hibbing ME , Fuqua C, Parsek MR et al. Bacterial competition: surviving and thriving in the microbial jungle. Nat Rev Microbio*l* 2010;8:15–25. 10.1038/nrmicro2259 19946288 PMC2879262

[9] Stubbendieck RM , Straight PD. Multifaceted interfaces of bacterial competition. J Bacterio*l* 2016;198:2145–55. 10.1128/JB.00275-16 27246570 PMC4966439

[10] Shealy NG , Yoo W, Byndloss MX. Colonization resistance: metabolic warfare as a strategy against pathogenic Enterobacteriaceae. Curr Opin Microbio*l* 2021;64:82–90. 10.1016/j.mib.2021.09.014 34688039 PMC8612973

[11] Castellanos N , Nakanouchi J, Yüzen DI et al. A study on *Acinetobacter baumannii* and *Staphylococcus aureus* strains recovered from the same infection site of a diabetic patient. Curr Microbio*l* 2019;76:842–7. 10.1007/s00284-019-01696-7 31053906 PMC6556059

[12] Furuno JP , Hebden JN, Standiford HC et al. Prevalence of methicillin-resistant *Staphylococcus aureus* and *Acinetobacter baumannii* in a long-term acute care facility. Am J Infect Contro*l* 2008;36:468–71. 10.1016/j.ajic.2008.01.003 18786448 PMC2853910

[13] Konovalova A , Søgaard-Andersen L. Close encounters: contact-dependent interactions in bacteria. Mol Microbio*l* 2011;81:297–301. 10.1111/j.1365-2958.2011.07711.x 21651624

[14] Ikryannikova LN , Kurbatov LK, Gorokhovets NV et al. Contact-dependent growth inhibition in bacteria: do not get too close! Int J Mol Sc*i* 2020;21:1–15. 10.3390/ijms21217990 PMC7662968 33121148

[15] Sancho S , Artero A, Zaragoza R et al. Impact of nosocomial polymicrobial bloodstream infections on the outcome in critically ill patients. Eur J Clin Microbiol Infect Di*s* 2012;31:1791–6. 10.1007/s10096-011-1503-8 22167257

[16] Bernards AT , Frénay HM, Lim BT et al. Methicillin-resistant *Staphylococcus aureus* and *Acinetobacter baumannii*: an unexpected difference in epidemiologic behavior. Am J Infect Contro*l* 1998;26:544–51. 10.1053/ic.1998.v26.a84555 9836836

[17] Manian FA , Griesenauer S, Senkel D et al. Isolation of *Acinetobacter baumannii* complex and methicillin-resistant *Staphylococcus aureus* from hospital rooms following terminal cleaning and disinfection: can we do better? Infect Control Hosp Epidemio*l* 2011;32:667–72. 10.1086/660357 21666397

[18] Li G , Shen W, Gong Y et al. Essential fitness repertoire of *Staphylococcus aureus* during co-infection with *Acinetobacter baumannii* in vivo. mSystem*s* 2022;7:e0033822, 1–14. 10.1128/msystems.00338-22 36040021 PMC9600432

[19] Boucher HW , Talbot GH, Bradley JS et al. Bad bugs, no drugs: no ESKAPE! An update from the Infectious Diseases Society of America. Clin Infect Di*s* 2009;48:1–12. 10.1086/595011 19035777

[20] Tacconelli E , Carrara E, Savoldi A et al. Discovery, research, and development of new antibiotics: the WHO priority list of antibiotic-resistant bacteria and tuberculosis. Lancet Infect Di*s* 2018;18:318–27. 10.1016/S1473-3099(17)30753-3 29276051

[21] Hardy BL , Bansal G, Hewlett KH et al. Antimicrobial activity of clinically isolated bacterial species against *Staphylococcus aureus*. Front Microbio*l* 2019;10:2977. 10.3389/fmicb.2019.02977 32010080 PMC6975196

[22] Smith NM , Ang A, Tan F et al. Interaction of *Staphylococcus aureus* and *Acinetobacter baumannii* during in vitro β-lactam exposure. Antimicrob Agents Chemothe*r* 2021;65:1–12. 10.1128/AAC.02414-20 PMC8097447 33495215

[23] Fernandez JS , Tuttobene MR, Montaña S et al. *Staphylococcus aureus* α-toxin effect on *Acinetobacter baumannii* behavior. Biology (Basel*)* 2022;11:1–13. 10.3390/biology11040570 PMC9028598 35453769

[24] Mottola C , Mendes JJ, Cristino JM et al. Polymicrobial biofilms by diabetic foot clinical isolates. Folia Microbiol (Praha*)* 2016;61:35–43. 10.1007/s12223-015-0401-3 26104539

[25] Medyukhina A , Timme S, Mokhtari Z et al. Image-based systems biology of infection. Cytometry *A* 2015;87:462–70. 10.1002/cyto.a.22638 25641512

[26] Young D , Stark J, Kirschner D. Systems biology of persistent infection: tuberculosis as a case study. Nat Rev Microbio*l* 2008;6:520–8. 10.1038/nrmicro1919 18536727

[27] Eckhardt M , Hultquist JF, Kaake RM et al. A systems approach to infectious disease. Nat Rev Gene*t* 2020;21:339–54. 10.1038/s41576-020-0212-5 32060427 PMC7839161

[28] Charlebois DA , Balázsi G. Modeling cell population dynamics. In Silico Bio*l* 2019;13:21–39. 10.3233/ISB-180470 30562900 PMC6598210

[29] Langmead B , Salzberg SL. Fast gapped-read alignment with Bowtie 2. Nat Method*s* 2012;9:357–9. 10.1038/nmeth.1923 22388286 PMC3322381

[30] Liao Y , Smyth GK, Shi W. featureCounts: an efficient general purpose program for assigning sequence reads to genomic features. Bioinformatic*s* 2014;30:923–30. 10.1093/bioinformatics/btt656 24227677

[31] Love MI , Huber W, Anders S. Moderated estimation of fold change and dispersion for RNA-seq data with DESeq2. Genome Bio*l* 2014;15:550. 10.1186/s13059-014-0550-8 25516281 PMC4302049

[32] Monecke S , Slickers P, Ehricht R. Assignment of *Staphylococcus aureus* isolates to clonal complexes based on microarray analysis and pattern recognition. FEMS Immunol Med Microbio*l* 2008;53:237–51. 10.1111/j.1574-695X.2008.00426.x 18507678

[33] Monecke S , Coombs G, Shore AC et al. A field guide to pandemic, epidemic and sporadic clones of methicillin-resistant *Staphylococcus aureus*. PLoS On*e* 2011;6:e17936, 1–24. 10.1371/journal.pone.0017936 21494333 PMC3071808

[34] Jones E , Oliphant T, Peterson P. SciPy: Open Source Scientific Tools for Pytho*n*, 2001.

[35] Hünniger K , Lehnert T, Bieber K et al. A virtual infection model quantifies innate effector mechanisms and *Candida albicans* immune escape in human blood. PLoS Comput Bio*l* 2014;10:e1003479. 10.1371/journal.pcbi.1003479 24586131 PMC3930496

[36] Lehnert T , Leonhardt I, Timme S et al. Ex vivo immune profiling in patient blood enables quantification of innate immune effector functions. Sci Re*p* 2021;11:12039. 10.1038/s41598-021-91362-5 34103589 PMC8187451

[37] Lehnert T , Timme S, Pollmächer J et al. Bottom-up modeling approach for the quantitative estimation of parameters in pathogen-host interactions. Front Microbio*l* 2015;6:608. 10.3389/fmicb.2015.00608 26150807 PMC4473060

[38] Prauße MTE , Lehnert T, Timme S et al. Predictive virtual infection modeling of fungal immune evasion in human whole blood. Front Immuno*l* 2018;9:560. 10.3389/fimmu.2018.00560 29619027 PMC5871695

[39] Timme S , Lehnert T, Prauße MTE et al. Quantitative simulations predict treatment strategies against fungal infections in virtual neutropenic patients. Front Immuno*l* 2018;9:667. 10.3389/fimmu.2018.00667 29670632 PMC5893870

[40] Peterson SB , Bertolli SK, Mougous JD. The central role of interbacterial antagonism in bacterial life. Curr Bio*l* 2020;30:R1203–r1214. 10.1016/j.cub.2020.06.103 33022265 PMC7595158

[41] Tuttobene MR , Fernández-García L, Blasco L et al. Quorum and light signals modulate acetoin/butanediol catabolism in *Acinetobacter* spp. Front Microbio*l* 2019;10:1376. 10.3389/fmicb.2019.01376 31281296 PMC6595428

[42] Xiao Z , Xu P. Acetoin metabolism in bacteria. Crit Rev Microbio*l* 2007;33:127–40. 10.1080/10408410701364604 17558661

[43] Li L , Pian Y, Chen S et al. Phenol-soluble modulin α4 mediates *Staphylococcus aureus*-associated vascular leakage by stimulating heparin-binding protein release from neutrophils. Sci Re*p* 2016;6:29373. 10.1038/srep29373 27383625 PMC4935938

[44] Novick RP , Geisinger E. Quorum sensing in staphylococci. Annu Rev Gene*t* 2008;42:541–64. 10.1146/annurev.genet.42.110807.091640 18713030

[45] Queck SY , Jameson-Lee M, Villaruz AE et al. RNAIII-independent target gene control by the agr quorum-sensing system: insight into the evolution of virulence regulation in *Staphylococcus aureus*. Mol Cel*l* 2008;32:150–8. 10.1016/j.molcel.2008.08.005 18851841 PMC2575650

[46] Bojer MS , Lindemose S, Vestergaard M et al. Quorum sensing-regulated phenol-soluble modulins limit persister cell populations in *Staphylococcus aureus*. Front Microbio*l* 2018;9:255. 10.3389/fmicb.2018.00255 29515541 PMC5826201

[47] Siwczak F , Cseresnyes Z, Hassan MIA et al. Human macrophage polarization determines bacterial persistence of *Staphylococcus aureus* in a liver-on-chip-based infection model. Biomaterial*s* 2022;287:121632. 10.1016/j.biomaterials.2022.121632 35728409

[48] Tuchscherr L , Pöllath C, Siegmund A et al. Clinical *S. aureus* isolates vary in their virulence to promote adaptation to the host. Toxins (Basel*)* 2019;11:1–15. 10.3390/toxins11030135 PMC6468552 30823631

[49] John J , Saranathan R, Adigopula LN et al. The quorum sensing molecule N-acyl homoserine lactone produced by *Acinetobacter baumannii* displays antibacterial and anticancer properties. Biofoulin*g* 2016;32:1029–47. 10.1080/08927014.2016.1221946 27643959

[50] Hardy BL , Dickey SW, Plaut RD et al. Corynebacterium pseudodiphtheriticum exploits *Staphylococcus aureus* virulence components in a novel polymicrobial defense strategy. MBi*o* 2019;10:1–24. 10.1128/mBio.02491-18 PMC6325251 30622190

[51] Matias C , Serrano I, Van-Harten S et al. Polymicrobial interactions influence the agr copy number in *Staphylococcus aureus* isolates from diabetic foot ulcers. Antonie Van Leeuwenhoe*k* 2018;111:2225–32. 10.1007/s10482-018-1103-z 29796774

[52] Ramsey MM , Freire MO, Gabrilska RA et al. *Staphylococcus aureus* shifts toward commensalism in response to *Corynebacterium* species. Front Microbio*l* 2016;7:1230. 10.3389/fmicb.2016.01230 27582729 PMC4988121

[53] Todd OA , Fidel PL Jr, Harro JM et al. *Candida albicans* augments *Staphylococcus aureus* virulence by engaging the staphylococcal agr quorum sensing system. MBi*o* 2019;10:1–16. 10.1128/mBio.00910-19 PMC6550526 31164467

[54] Gomes-Fernandes M , Gomez AC, Bravo M et al. Strain-specific interspecies interactions between co-isolated pairs of *Staphylococcus aureus* and *Pseudomonas aeruginosa* from patients with tracheobronchitis or bronchial colonization. Sci Re*p* 2022;12:3374. 10.1038/s41598-022-07018-5 35233050 PMC8888623

[55] Le NH , Pinedo V, Lopez J et al. Killing of Gram-negative and Gram-positive bacteria by a bifunctional cell wall-targeting T6SS effector. Proc Natl Acad Sci US*A* 2021;118:1–6. 10.1073/pnas.2106555118 PMC8501793 34588306

[56] Carruthers MD , Nicholson PA, Tracy EN et al. *Acinetobacter baumannii* utilizes a type VI secretion system for bacterial competition. PLoS On*e* 2013;8:e59388. 10.1371/journal.pone.0059388 23527179 PMC3602014

[57] Troha K , Ayres JS. Metabolic adaptations to infections at the organismal level. Trends Immuno*l* 2020;41:113–25. 10.1016/j.it.2019.12.001 31959515 PMC7409656

[58] Tuchscherr L , Medina E, Hussain M et al. *Staphylococcus aureus* phenotype switching: an effective bacterial strategy to escape host immune response and establish a chronic infection. EMBO Mol Me*d* 2011;3:129–41. 10.1002/emmm.201000115 21268281 PMC3395110

[59] Wong Fok Lung T , Chan LC, Prince A et al. *Staphylococcus aureus* adaptive evolution: recent insights on how immune evasion, immunometabolic subversion and host genetics impact vaccine development. Front Cell Infect Microbio*l* 2022;12:1060810. 10.3389/fcimb.2022.1060810 36636720 PMC9831658

[60] Goormaghtigh F , Van Bambeke F. Understanding *Staphylococcus aureus* internalisation and induction of antimicrobial tolerance. Expert Rev Anti-Infect The*r* 2024;22:87–101. 10.1080/14787210.2024.2303018 38180805

[61] Zwe YH , Yadav M, Ten MMZ et al. Bacterial antagonism of *Chromobacterium haemolyticum* and characterization of its putative type VI secretion system. Res Microbio*l* 2022;173:103918. 10.1016/j.resmic.2021.103918 34906677

[62] Resch A , Rosenstein R, Nerz C et al. Differential gene expression profiling of *Staphylococcus aureus* cultivated under biofilm and planktonic conditions. Appl Environ Microbio*l* 2005;71:2663–76. 10.1128/AEM.71.5.2663-2676.2005 15870358 PMC1087559

