# Supplementary material for: Competitive inhibition and mutualistic growth in co-infections: deciphering Staphylococcus aureus–Acinetobacter baumannii interaction dynamics
Source: ISME Commun. 2024 Jun 10;4(1):ycae077. doi: 10.1093/ismeco/ycae077 (PMC11221087; doi:10.1093/ismeco/ycae077)
Supplement: 2024-06-20_Supplementary_Figures_ycae077 [file 2024-06-20_supplementary_figures_ycae077.docx]

**Supplementary Figures**


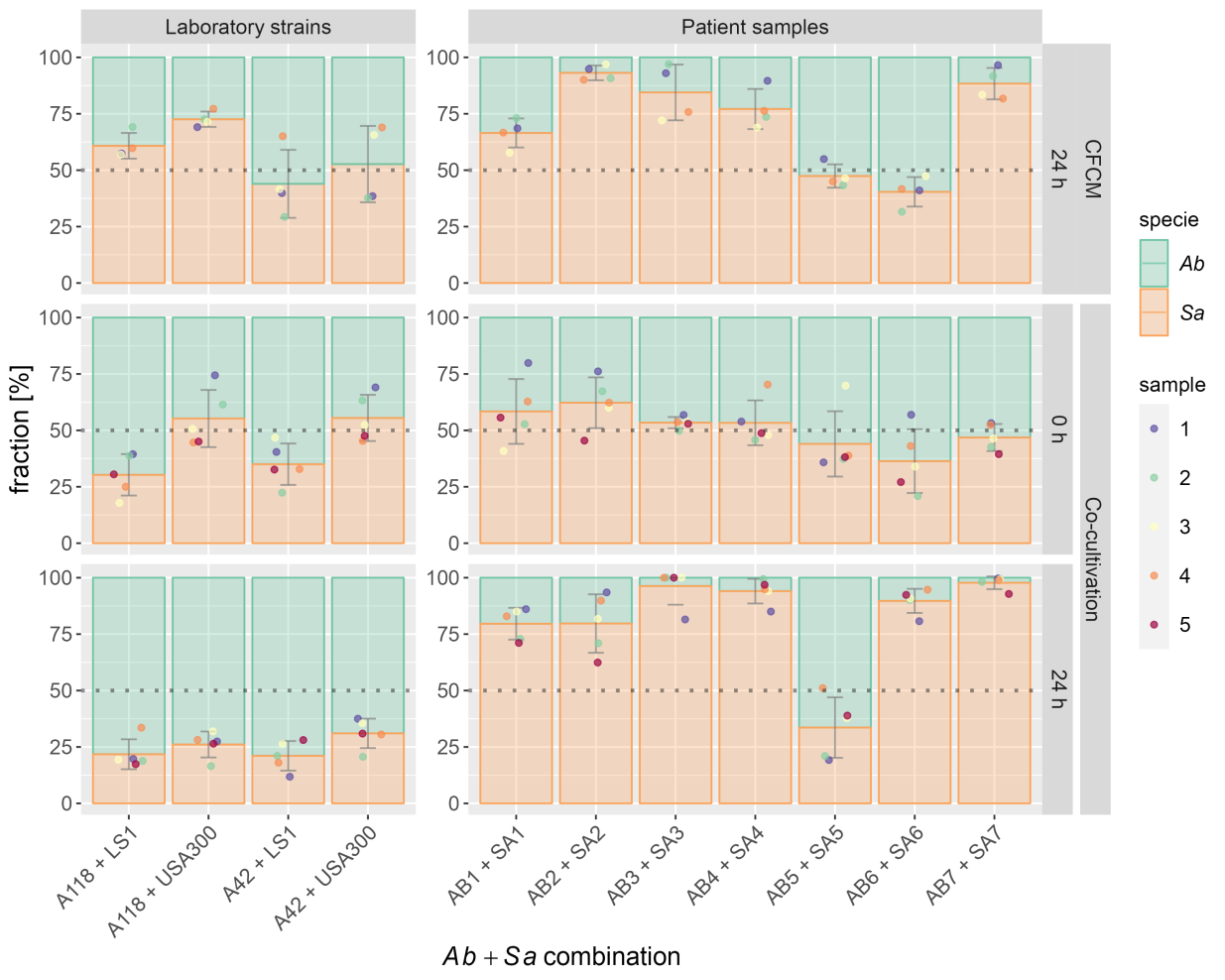


Supplementary Figure 1 Relative CFU fraction $\boldsymbol{CF}\boldsymbol{U}_{\boldsymbol{fraction}}^{\boldsymbol{s}}\mathbf{=}\frac{\boldsymbol{CF}\boldsymbol{U}_{\boldsymbol{s}\mathbf{+}\boldsymbol{o}}^{\boldsymbol{s}}}{\boldsymbol{CF}\boldsymbol{U}_{\boldsymbol{s}\mathbf{+}\boldsymbol{o}}^{\boldsymbol{s}}\boldsymbol{+CF}\boldsymbol{U}_{\boldsymbol{s}\mathbf{+}\boldsymbol{o}}^{\boldsymbol{o}}}\boldsymbol{\cdot}\boldsymbol{100\%}$ of strain $\boldsymbol{s}$ in CFCM and Co-cultivation experiments compared to the total CFU count of strain $\boldsymbol{s}$ and the other strain $\boldsymbol{o}$ for laboratory strains and patient samples at the beginning (0 h) and the end (24 h) of the experiment. Colored points refer to the individual measurements (samples), Colored bars indicate the average fraction of the respective specie and error bars denote the standard deviation.


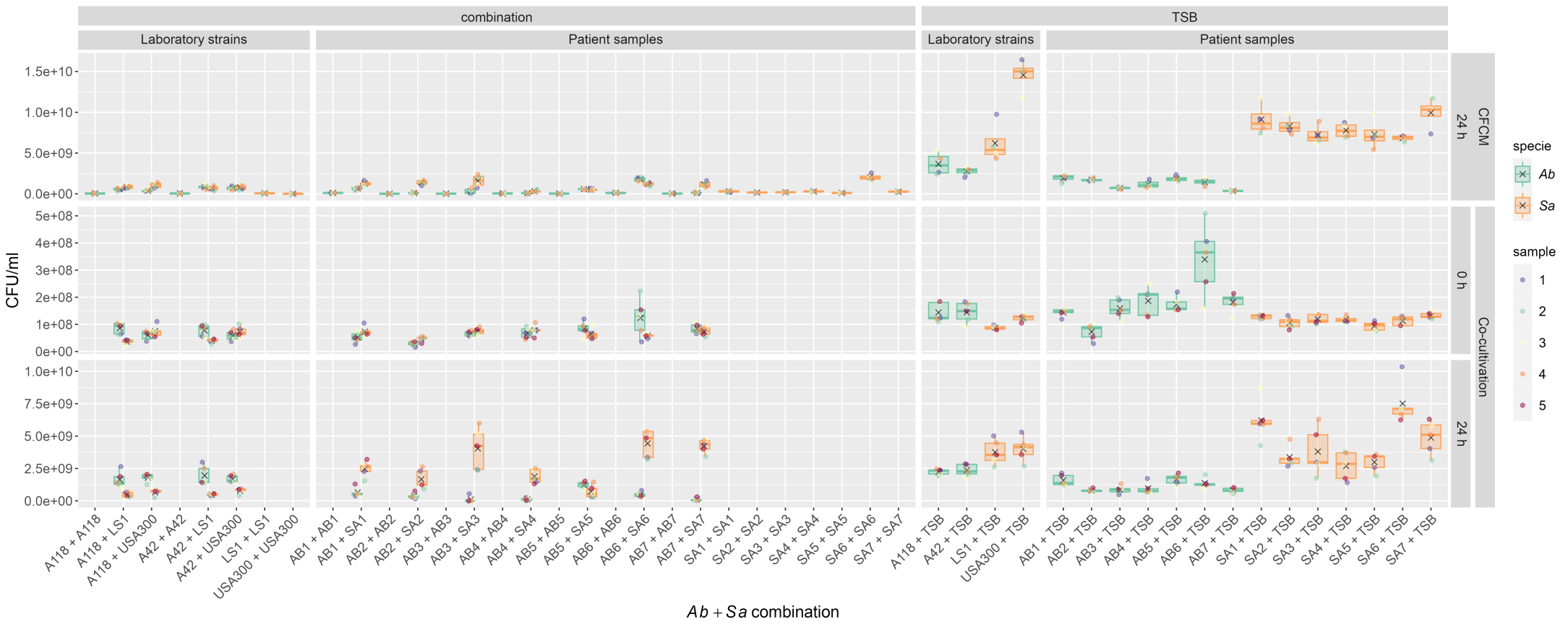


Supplementary Figure 2 CFU counts of laboratory strains and patient samples at the beginning of the experiments (0 h) after 24 h for CFCM and co-cultivation experiments as well as for control experiments with TSB medium. Colored points refer to the individual measurements (samples), Grey crosses denote the mean value and colored boxes denote the interquartile range (IQR) of the respective specie between the 25% and 75% quantile, with the median shown as a horizontal line. Whiskers show the interval between the $\boldsymbol{75\%}\text{quantile}\boldsymbol{+ 1.5\cdot}\text{IQR}$ and the $\boldsymbol{25\%}\text{quantile}\boldsymbol{- 1.5\cdot}\text{IQR}$.


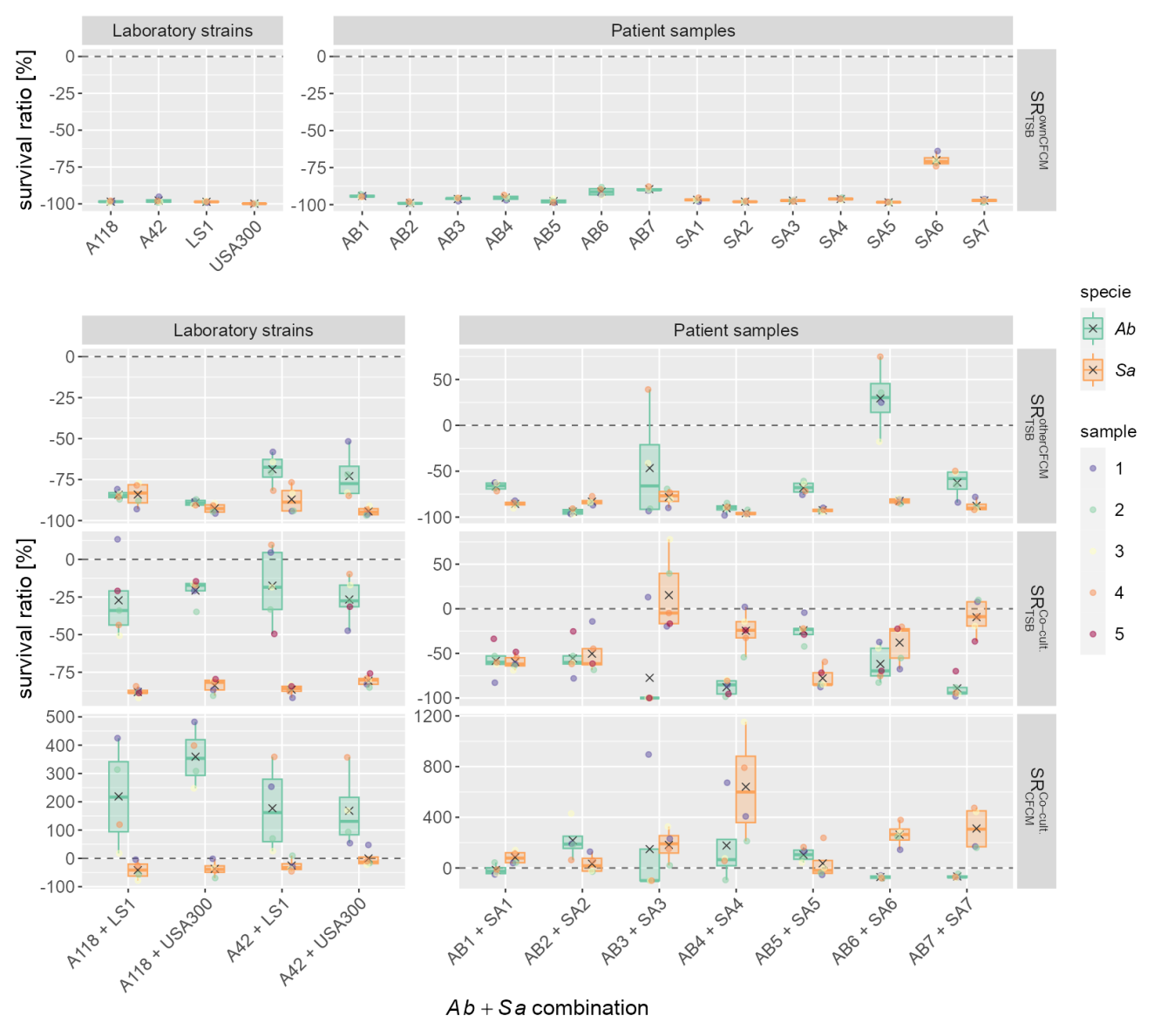


Supplementary Figure 3 Survival ratio $\boldsymbol{SR}$ of the CFU counts after 24h. The first row shows the $\boldsymbol{S}\boldsymbol{R}_{\boldsymbol{TSB}}^{\boldsymbol{ownCFCM}}\boldsymbol{(s)}$ of strain $\boldsymbol{s}$, which is given by $\boldsymbol{SR}_{\boldsymbol{TSB}}^{\boldsymbol{CFCM}}\boldsymbol{(s)=}\frac{\boldsymbol{CF}\boldsymbol{U}_{\boldsymbol{s}}^{\boldsymbol{ownCFCM}}\boldsymbol{- CF}\boldsymbol{U}_{\boldsymbol{s}}^{\boldsymbol{TSB}}}{\boldsymbol{CF}\boldsymbol{U}_{\boldsymbol{s}}^{\boldsymbol{TSB}}}$, with the CFU count in its own CFCM ($\boldsymbol{CF}\boldsymbol{U}_{\boldsymbol{s}}^{\boldsymbol{ownCFCM}}$) and its CFU count in the TSB control experiment ($\boldsymbol{CF}\boldsymbol{U}_{\boldsymbol{s}}^{\boldsymbol{TSB}}$). The second row shows the survival ratio $\boldsymbol{S}\boldsymbol{R}_{\boldsymbol{TSB}}^{\boldsymbol{otherCFCM}}\boldsymbol{(s)}$ of strain $\boldsymbol{s}$, which is given by $\boldsymbol{SR}_{\boldsymbol{TSB}}^{\boldsymbol{otherCFCM}}\boldsymbol{(s)=}\frac{\boldsymbol{CF}\boldsymbol{U}_{\boldsymbol{s+o}}^{\boldsymbol{otherCFCM}}\boldsymbol{- CF}\boldsymbol{U}_{\boldsymbol{s}}^{\boldsymbol{TSB}}}{\boldsymbol{CF}\boldsymbol{U}_{\boldsymbol{s}}^{\boldsymbol{TSB}}}$ , with the CFU count in the CFCM of the other strain $\boldsymbol{o}$ ($\boldsymbol{CF}\boldsymbol{U}_{\boldsymbol{s+o}}^{\boldsymbol{otherCFCM}}$) and its CFU count in the TSB control experiment ($\boldsymbol{CF}\boldsymbol{U}_{\boldsymbol{s}}^{\boldsymbol{TSB}}$). The third row shows the survival ratio $\boldsymbol{S}\boldsymbol{R}_{\boldsymbol{TSB}}^{\boldsymbol{Co-cult.}}\boldsymbol{(s)}$ of strain $\boldsymbol{s}$, which is given by $\boldsymbol{SR}_{\boldsymbol{TSB}}^{\boldsymbol{Co-cult.}}\boldsymbol{(s)=}\frac{\boldsymbol{CF}\boldsymbol{U}_{\boldsymbol{s+o}}^{\boldsymbol{Co-cult.}}\boldsymbol{- CF}\boldsymbol{U}_{\boldsymbol{s}}^{\boldsymbol{TSB}}}{\boldsymbol{CF}\boldsymbol{U}_{\boldsymbol{s}}^{\boldsymbol{TSB}}}$ , with the CFU count in co-cultivation with the other strain $\boldsymbol{o}$ ($\boldsymbol{CF}\boldsymbol{U}_{\boldsymbol{s+o}}^{\boldsymbol{Co-cult.}}$) and its CFU count in the TSB control experiment ($\boldsymbol{CF}\boldsymbol{U}_{\boldsymbol{s}}^{\boldsymbol{TSB}}$). The fourth row shows the survival ratio $\boldsymbol{S}\boldsymbol{R}_{\boldsymbol{otherCFCM}}^{\boldsymbol{Co-cult.}}\boldsymbol{(s)}$ of strain $\boldsymbol{s}$, which is given by $\boldsymbol{SR}_{\boldsymbol{other CFCM}}^{\boldsymbol{Co-cult.}}\boldsymbol{(s)=}\frac{\boldsymbol{CF}\boldsymbol{U}_{\boldsymbol{s+o}}^{\boldsymbol{otherCFCM}}\boldsymbol{-CF}\boldsymbol{U}_{\boldsymbol{s+o}}^{\boldsymbol{otherCFCM}}}{\boldsymbol{CF}\boldsymbol{U}_{\boldsymbol{s+o}}^{\boldsymbol{otherCFCM}}}$ , with the CFU count in co-cultivation with the other strain $\boldsymbol{o}$ ($\boldsymbol{CF}\boldsymbol{U}_{\boldsymbol{s+o}}^{\boldsymbol{Co-cult.}}$) and its CFU count in the TSB control experiment ($\boldsymbol{CF}\boldsymbol{U}_{\boldsymbol{s}}^{\boldsymbol{TSB}}$). Colored points refer to the individual measurements (samples), Grey crosses denote the mean value and colored boxes denote the interquartile range (IQR) of the respective specie between the 25% and 75% quantile, with the median shown as a horizontal line. Whiskers show the interval between the $\boldsymbol{75\%}\text{quantile}\boldsymbol{+ 1.5\cdot}\text{IQR}$ and the $\boldsymbol{25\%}\text{quantile}\boldsymbol{- 1.5\cdot}\text{IQR}$.


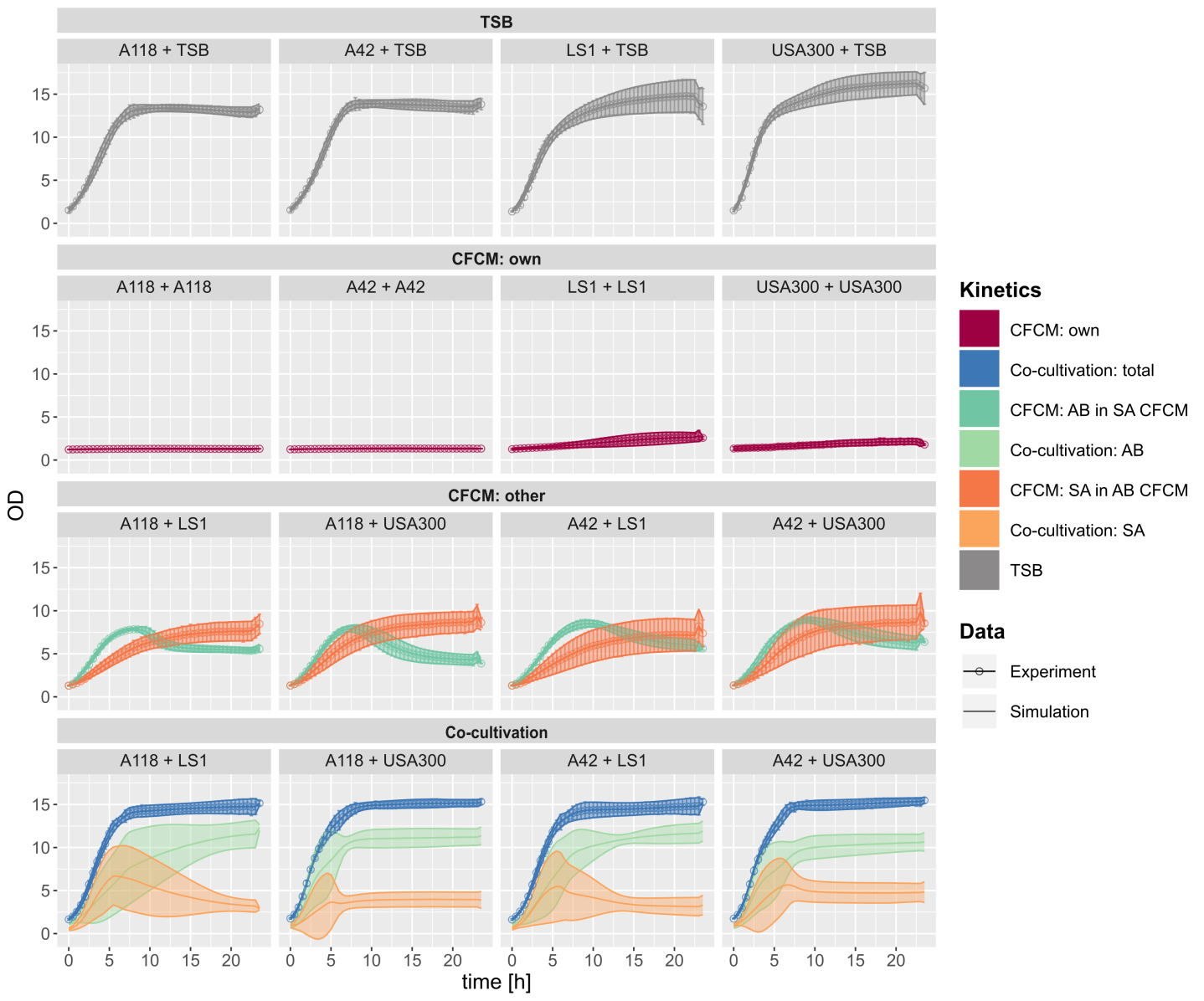


Supplementary Figure 4 Growth curves measured as optical density (OD) over 24 h for CFCM and co-cultivation experiments as well as in TSB as a control for laboratory strains.


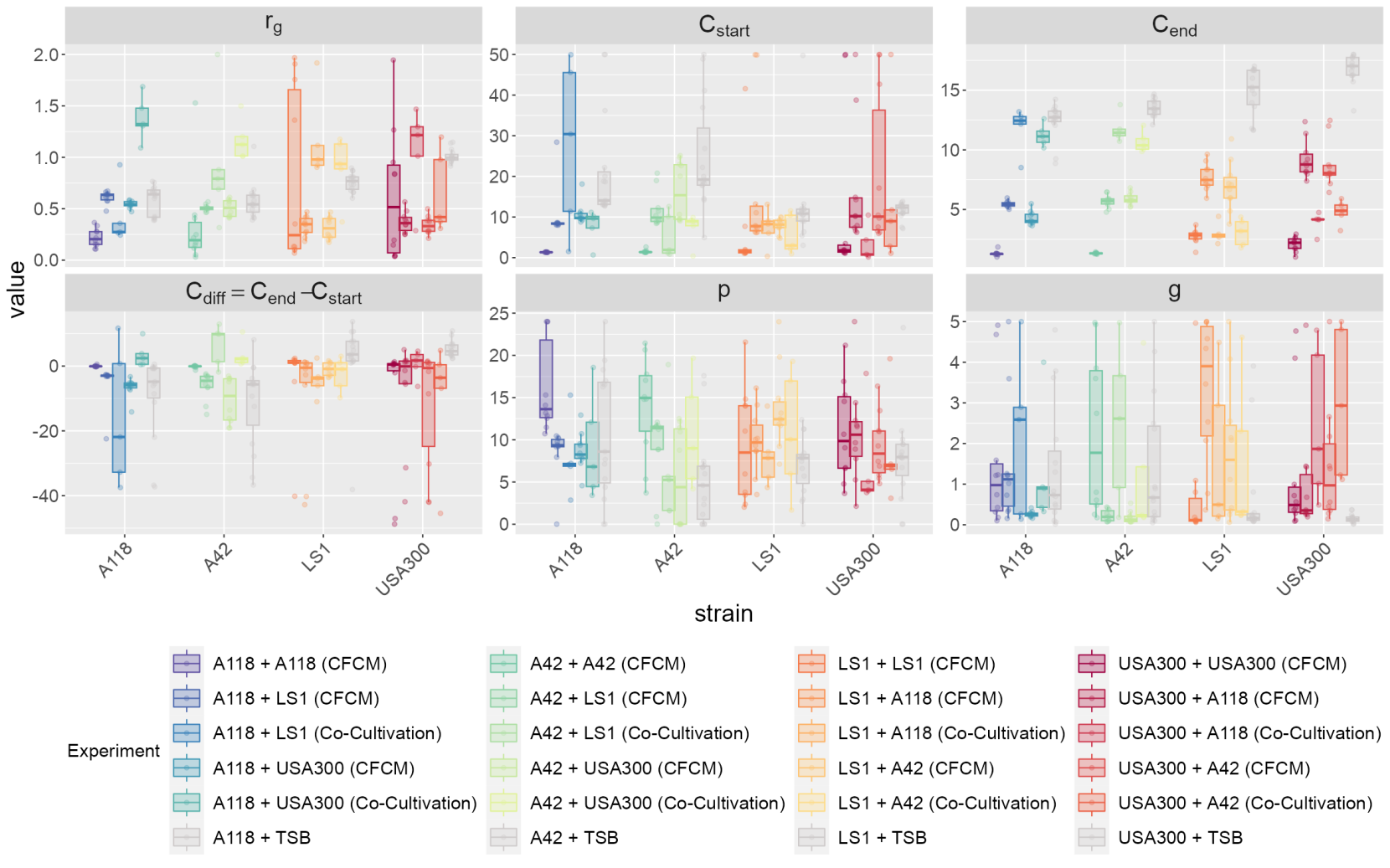


Supplementary Figure 5 Estimated model parameters for laboratory strains. Points refer to the estimated parameters for the individual samples, Boxes denote the interquartile range (IQR) of the respective strain between the 25% and 75% quantile, with the median shown as a horizontal line. Whiskers show the interval between the $\boldsymbol{75\%}\text{quantile}\boldsymbol{+ 1.5\cdot}\text{IQR}$ and the $\boldsymbol{2}\boldsymbol{5\%}\text{quantile}\boldsymbol{- 1.5\cdot}\text{IQR}$. Colors refer to the different experiments

*Sa* USA300

*Ab* 118


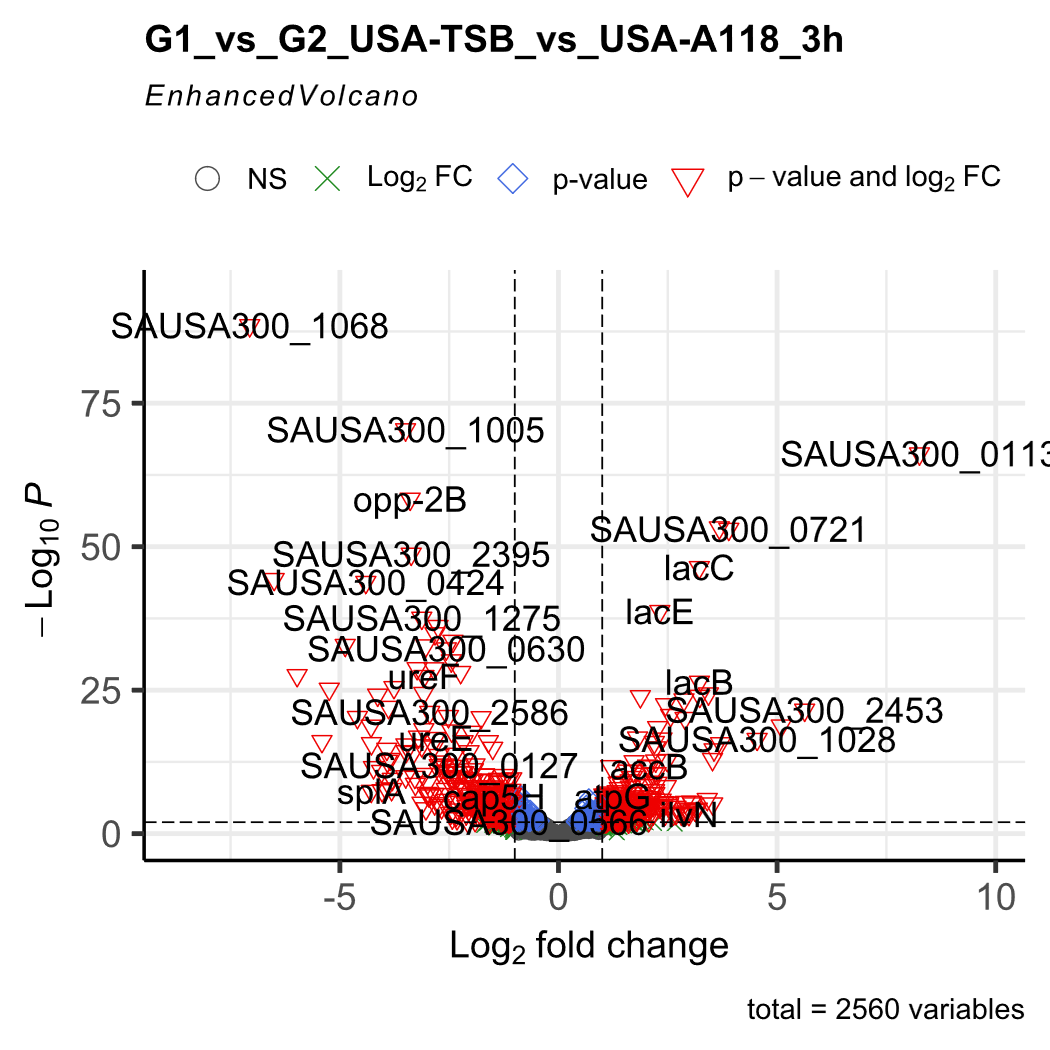

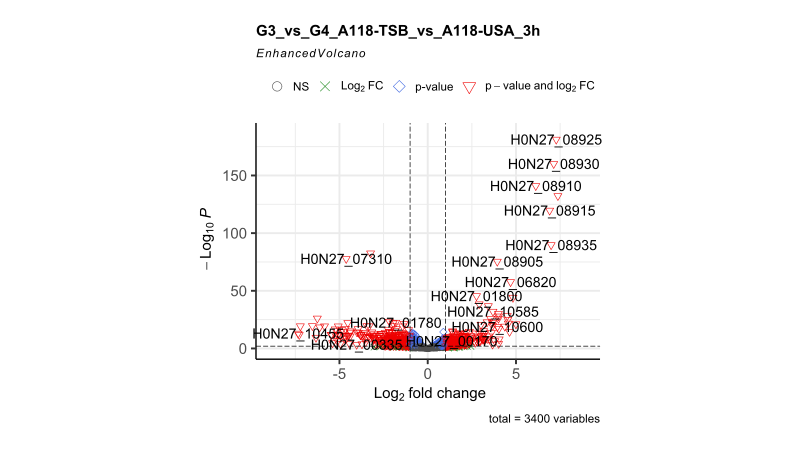


**A**

**B**

Supplementary Figure 6 Vulcano plot of RNA seq analysis. (A) Differentially expressed genes of *Ab* A118 in TSB medium compared to growth in *Sa* USA300 CFCM after 3h. (B) Differentially expressed genes of *Sa* USA300 in TSB medium compared to growth in *Ab* A118 CFCM after 3 h.

**
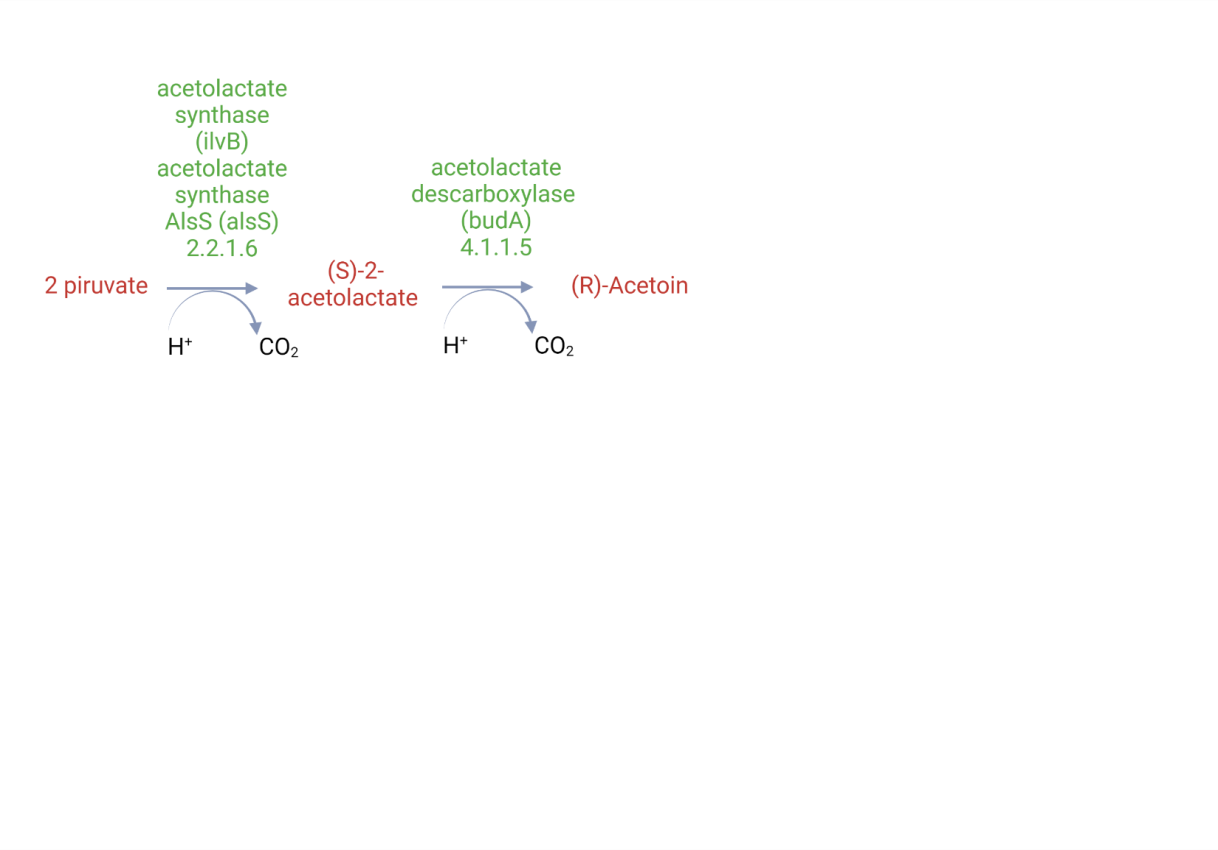
**

Supplementary Figure 7 Acetoin biosynthesis (Adapted from biocyc: <https://biocyc.org>)


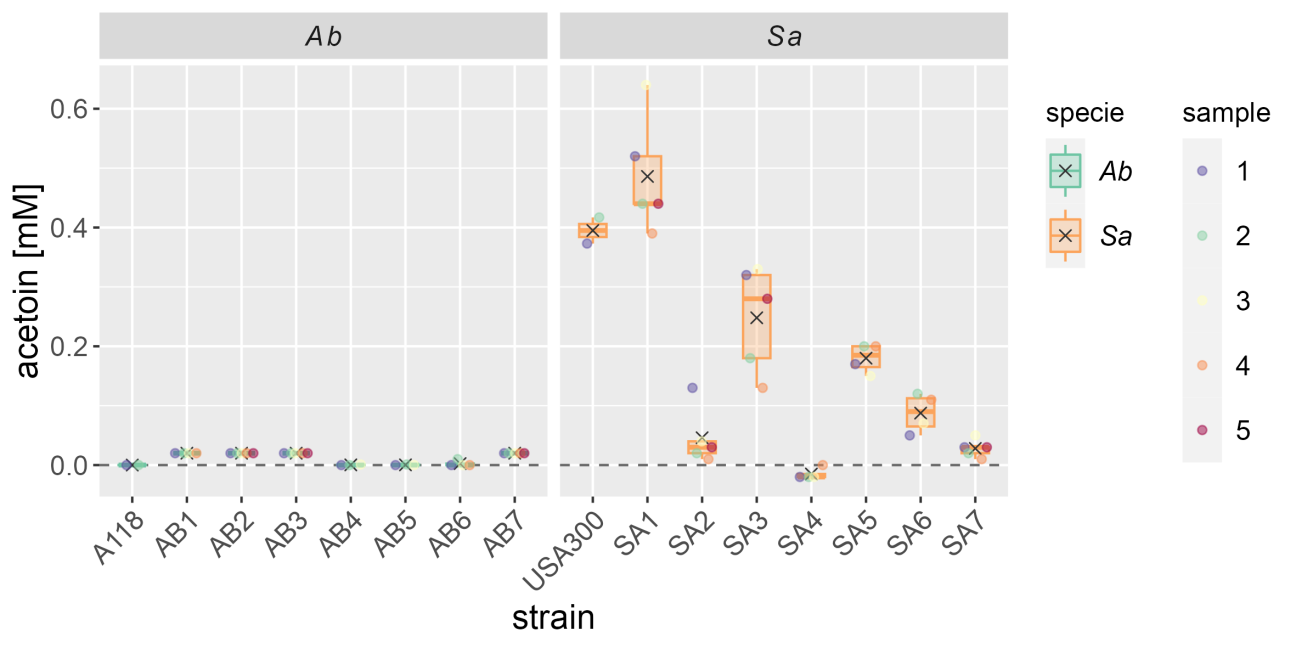


Supplementary Figure 8 Measured acetoin from CFCM (24 h) in *AB* and *SA* strains

Quorum sensing – Treatment of CFCM


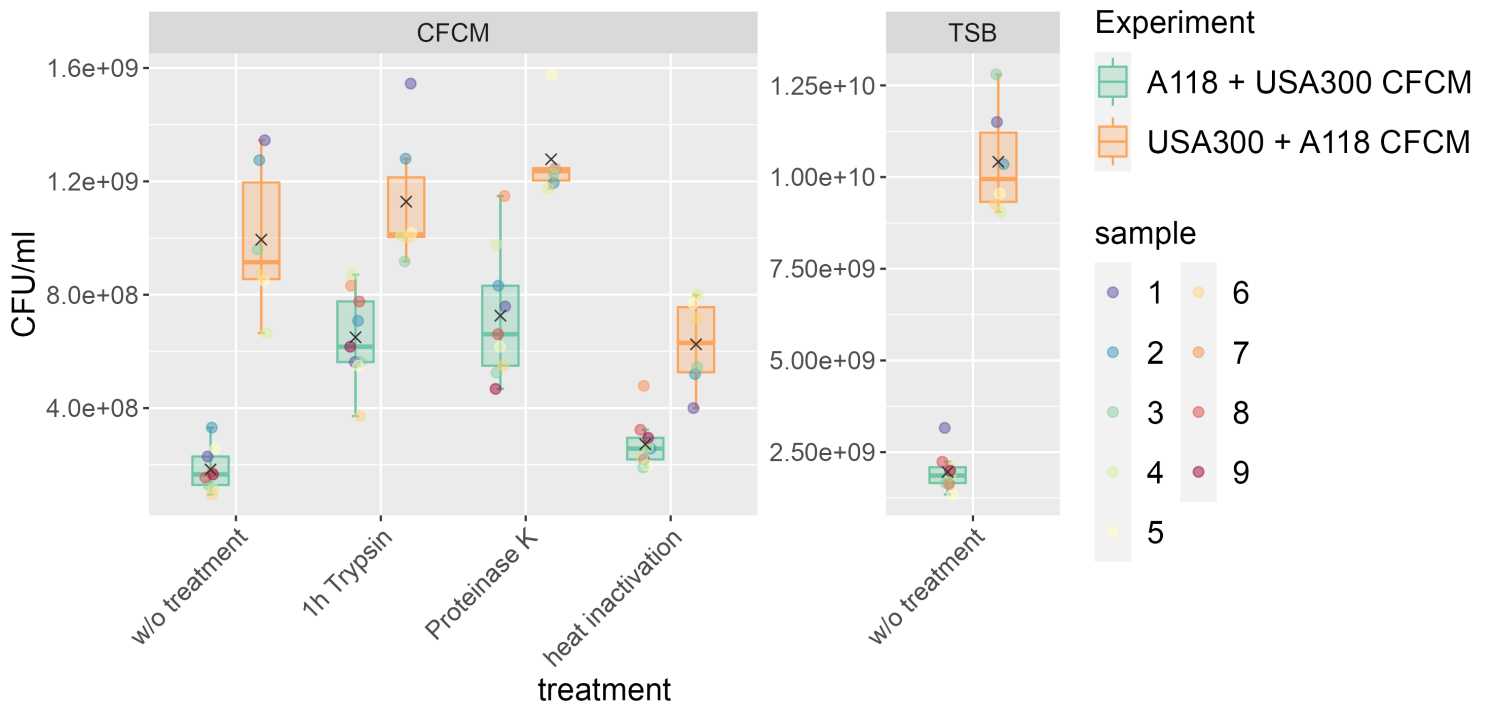


Supplementary Figure 9 CFU counts of laboratory strains and patient samples at the beginning of the experiments (0 h) after 24 h in CFCM with and without treatment as well as in TSB medium. Colored points refer to the individual measurements (samples), Grey crosses denote the mean value and colored boxes denote the interquartile range (IQR) of the respective specie between the 25% and 75% quantile, with the median shown as a horizontal line. Whiskers show the interval between the $\boldsymbol{75\%}\text{quantile}\boldsymbol{+ 1.5\cdot}\text{IQR}$ and the $\boldsymbol{25\%}\text{quantile}\boldsymbol{- 1.5\cdot}\text{IQR}$.


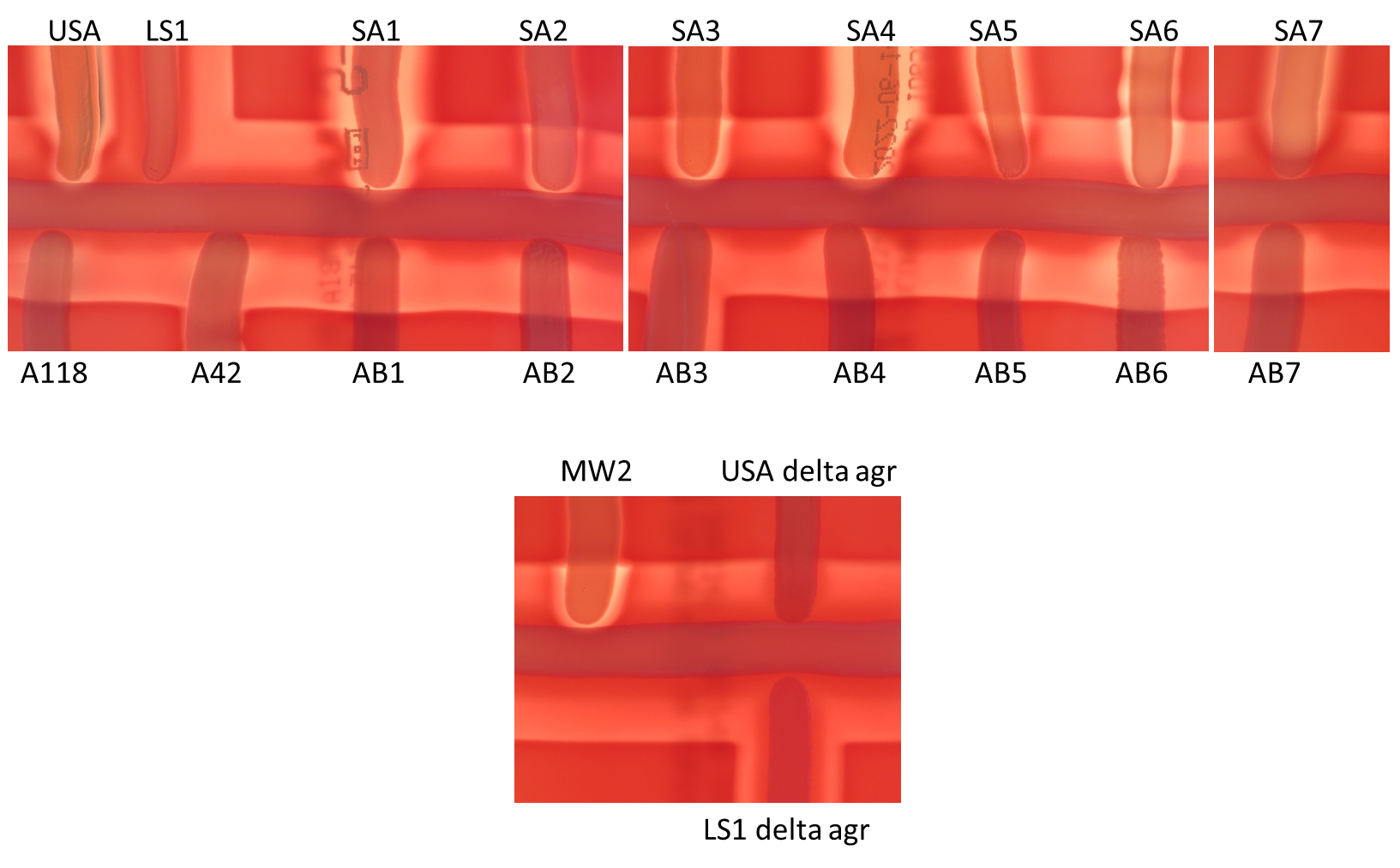


Supplementary Figure 10 CAMP test to test functional expression of *agr* gene.


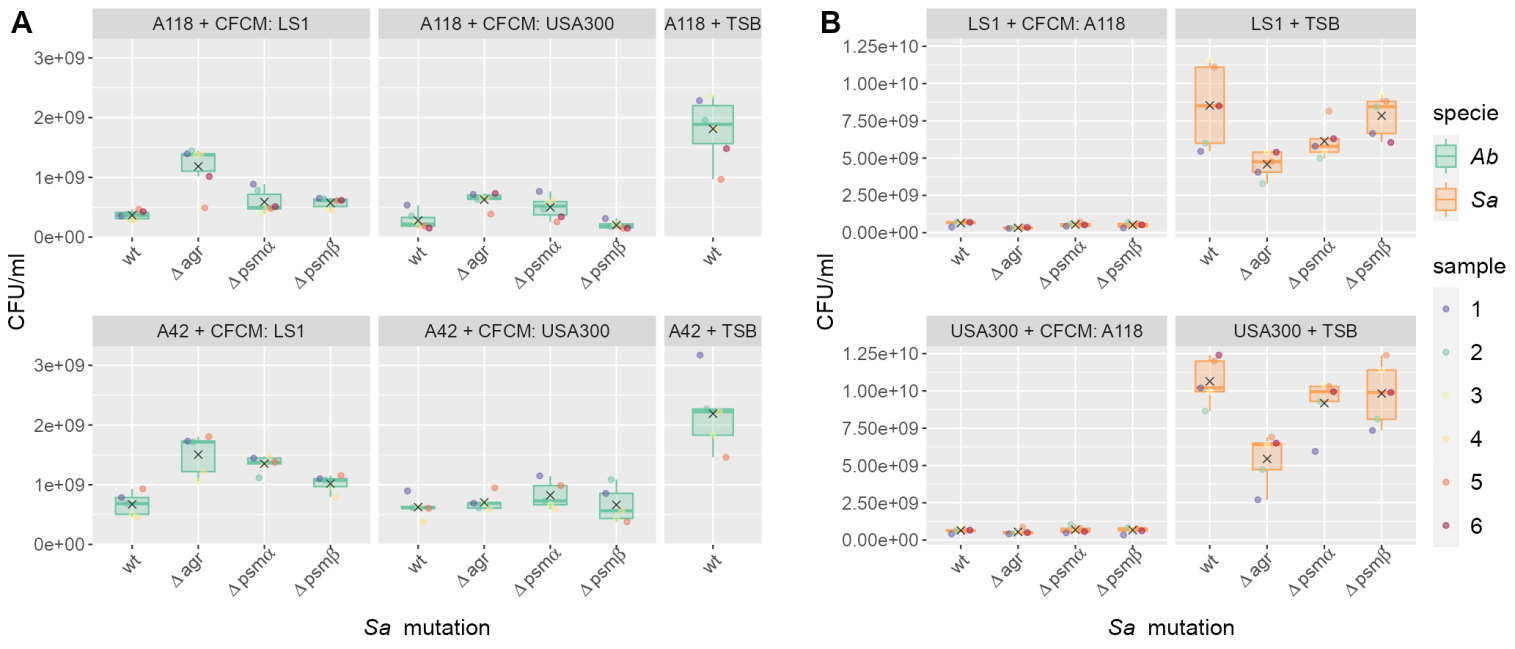


Supplementary Figure 11 CFU counts of (A) *Ab* A118 and A42 in CFCM from *Sa* LS1 and USA300 wt, *Δagr, Δpsmα* and *Δpsmβ*  mutants as well as in TSB medium and of (B) *Sa* LS1 and USA300 wt, *Δagr, Δpsmα* and *Δpsmβ* mutants in *Ab* A118 CFCM as well as in TSB medium. Colored points refer to the individual measurements (samples), Grey crosses denote the mean value and colored boxes denote the interquartile range (IQR) of the respective specie between the 25% and 75% quantile, with the median shown as a horizontal line. Whiskers show the interval between the $\boldsymbol{75\%}\text{quantile}\boldsymbol{+ 1.5\cdot}\text{IQR}$ and the $\boldsymbol{25\%}\text{quantile}\boldsymbol{- 1.5\cdot}\text{IQR}$.


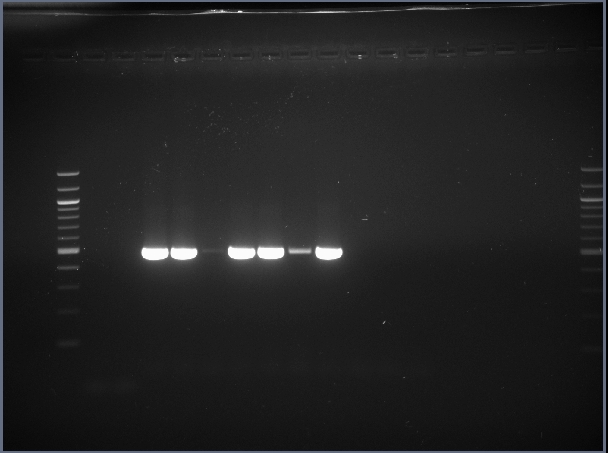


100bp Marker

A118

A42

AB1

AB2

AB3

AB4

AB5

AB6

AB7

H2O Control

Supplementary Figure 12 PCR for T6SS gene amplify in all *AB* strains

***
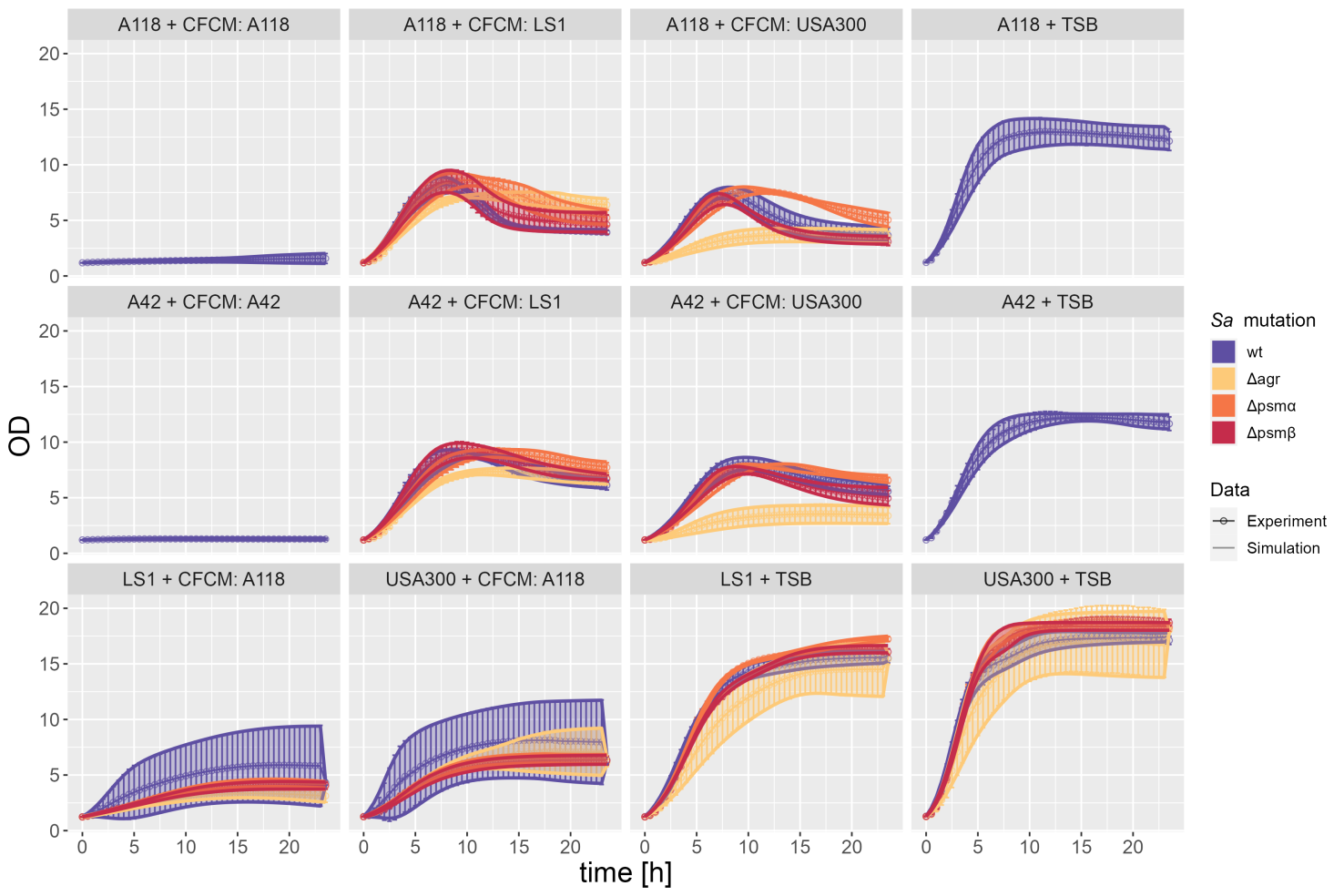
***

Supplementary Figure 13 Growth curves measured as optical density (OD) over 24 h for growth of *Ab* A118 (first row) and A42 in their own CFCM, in *Sa* CFCM or in TSB control medium, as well as of *Sa* LS1 and USA300 (third row) in Ab CFCM or in TSB control medium. Colors denote Sa mutations.


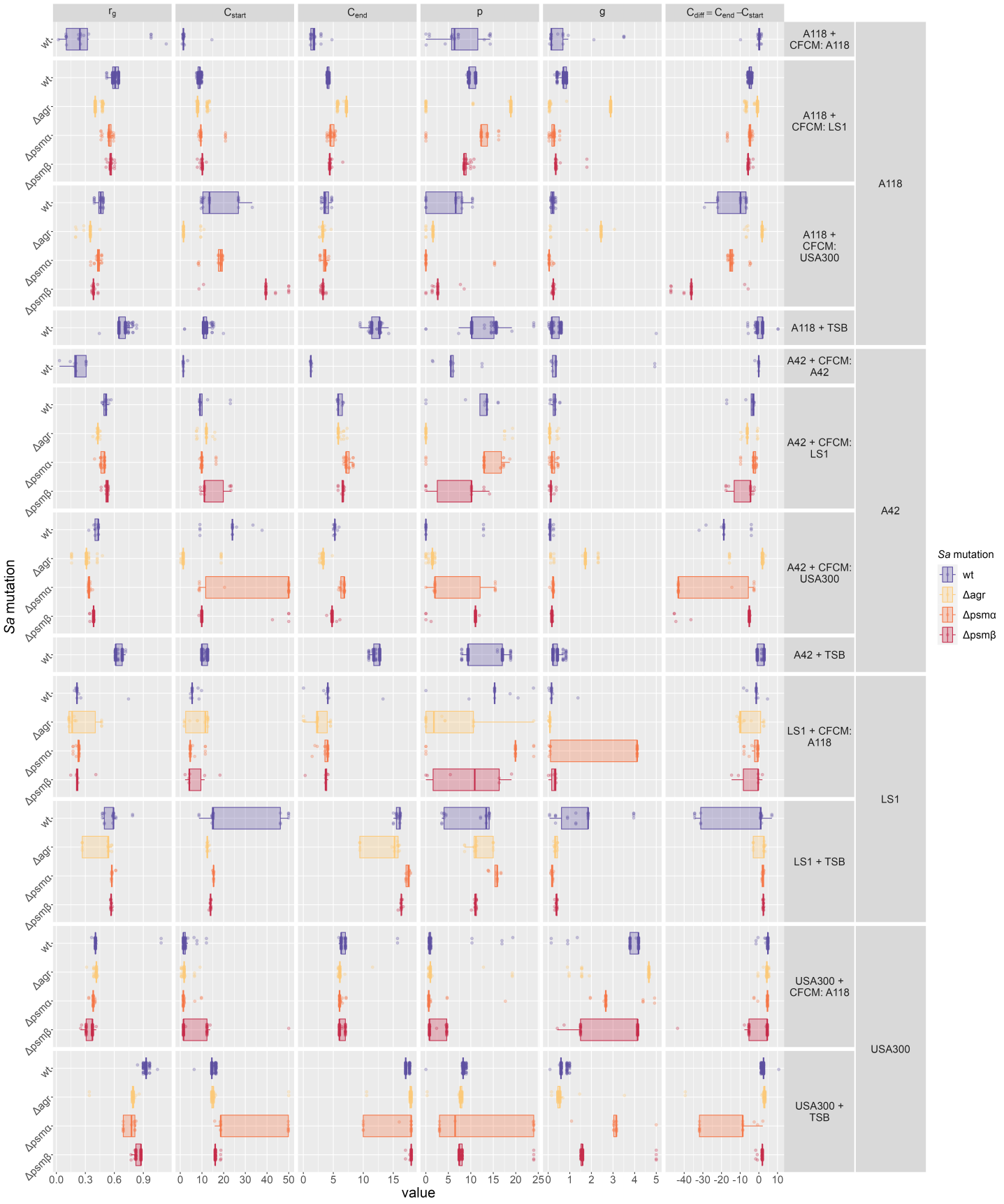


Supplementary Figure 14 Estimated model parameters for growth of *Ab* A118 (first row) and A42 in their own CFCM, in *Sa* CFCM or in TSB control medium, as well as of *Sa* LS1 and USA300 (third row) in Ab CFCM or in TSB control medium. Colors denote Sa mutations. Points refer to the estimated parameters for the individual samples, Boxes denote the interquartile range (IQR) of the respective strain between the 25% and 75% quantile, with the median shown as a horizontal line. Whiskers show the interval between the $\boldsymbol{75\%}\text{quantile}\boldsymbol{+ 1.5\cdot}\text{IQR}$ and the $\boldsymbol{25\%}\text{quantile}\boldsymbol{- 1.5\cdot}\text{IQR}$.


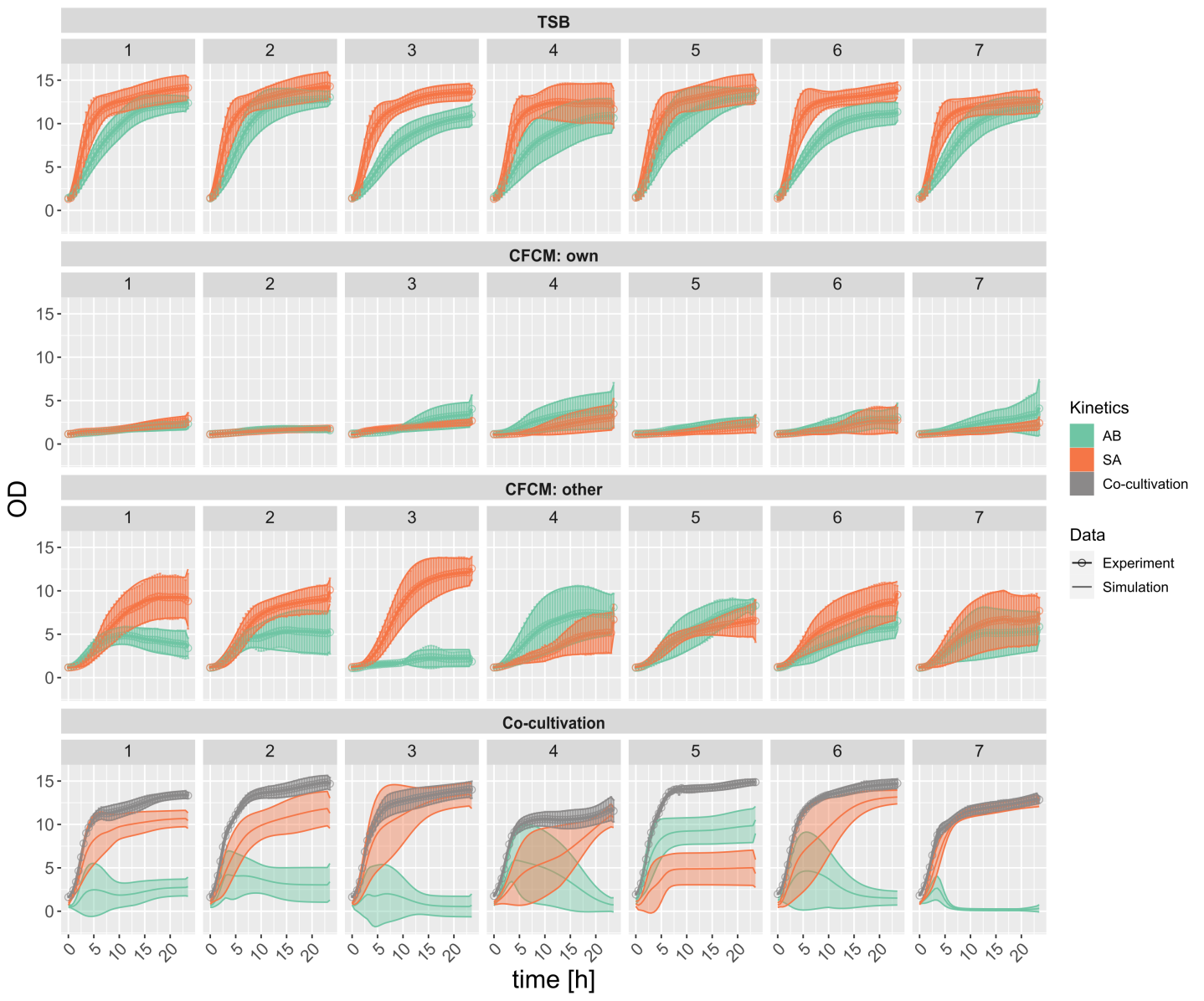


Supplementary Figure 15 Growth curves measured as optical density (OD) over 24 h for CFCM and co-cultivation experiments as well as in TSB as a control for patient samples.


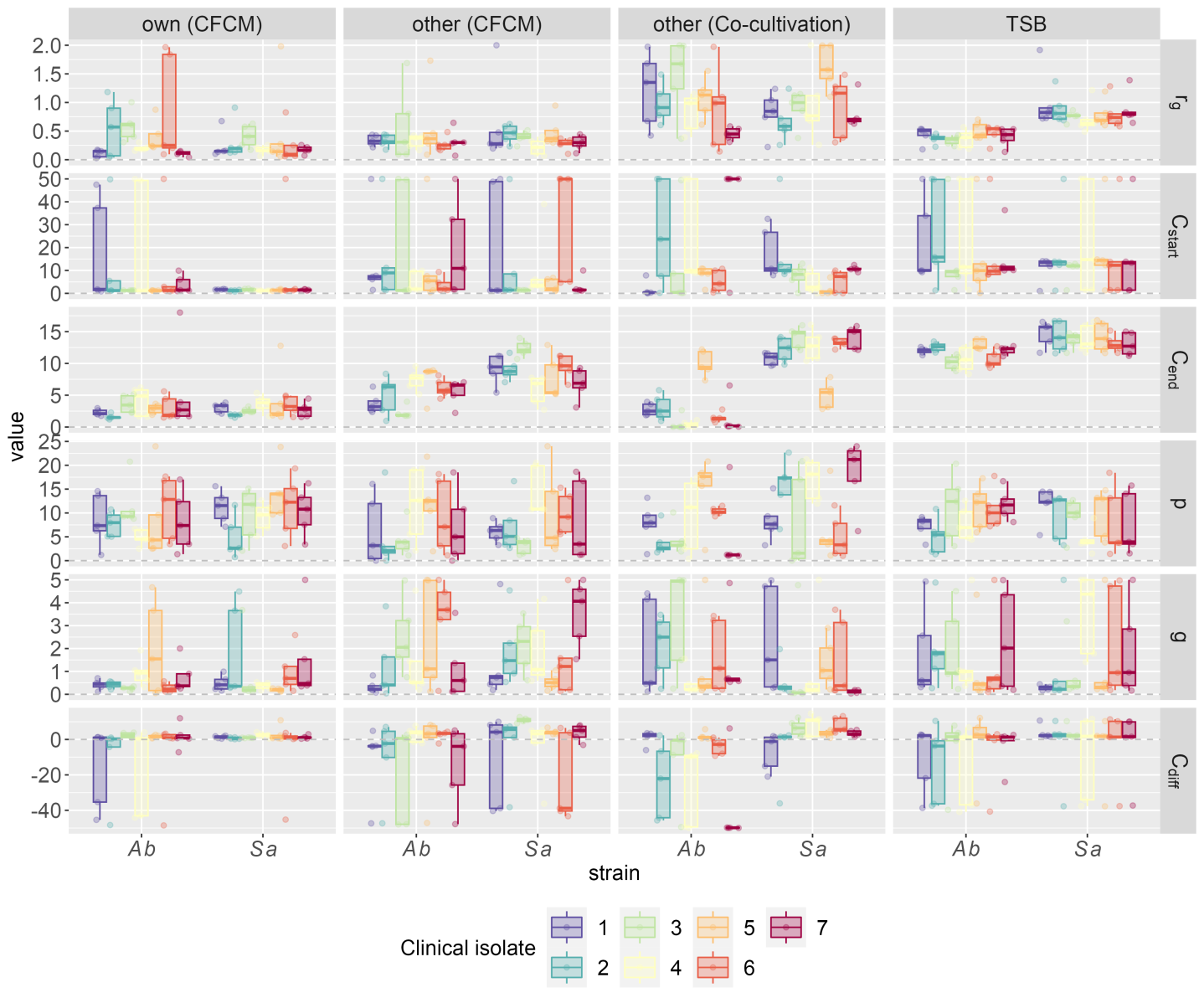


Supplementary Figure 16 Estimated model parameters for patient samples. Points refer to the estimated parameters for the individual samples, Boxes denote the interquartile range (IQR) of the respective specie between the 25% and 75% quantile, with the median shown as a horizontal line. Whiskers show the interval between the $\boldsymbol{75\%}\text{quantile}\boldsymbol{+ 1.5\cdot}\text{IQR}$ and the $\boldsymbol{25\%}\text{quantile}\boldsymbol{- 1.5\cdot}\text{IQR}$. Colors refer to the different patient samples.
