# Supplementary material for: Competitive inhibition and mutualistic growth in co-infections: deciphering Staphylococcus aureus–Acinetobacter baumannii interaction dynamics
Source: ISME Commun. 2024 Jun 10;4(1):ycae077. doi: 10.1093/ismeco/ycae077 (PMC11221087; doi:10.1093/ismeco/ycae077)
Supplement: Table_S1_strains_ycae077 [file table_s1_strains_ycae077.docx]

**Bacterial strains used in this study**

|  | **Specie** | **Strain** | **Source** | **Reference** |
| --- | --- | --- | --- | --- |
| **Laboratory strains** | *A. baumannii* | A118 | Bloodstream isolate recovered from a patient in an intensive care unit | Ramirez *et al*. 2010 |
|  | *A. baumannii* | A42 | MDR clinical strain isolated from sputum | Vilacoba *et al.* 2013 |
|  | *S. aureus* | LS1 | Murine arthritis isolate,  septic arthritis isolate | Bremell *et al.* 1992 |
|  | *S. aureus* | USA300 | Highly successful S. aureus clone that emerged in the community and quickly spread through the North American continent to become the leading cause of MRSA infection even in healthcare settings | Boyle-Vavra and Daum *et al.* 2007 |
|  | *A. baumannii* | AB1 | Isolated from tracheal/bronchial secret | This study |
| **Patient samples** | *A. baumannii* | AB2 | Isolated from surface wound smear | This study |
|  | *A. baumannii* | AB3 | Isolated from nose/throat smear | This study |
|  | *A. baumannii* | AB4 | Isolated from urine from kidney fistula | This study |
|  | *A. baumannii* | AB5 | Isolated from inner wound smear | This study |
|  | *A. baumannii* | AB6 | Isolated from throat smear | This study |
|  | *A. baumannii* | AB7 | Isolated from throat smear | This study |
|  | *S. aureus* | SA1 | Isolated from tracheal/bronchial secret | This study |
|  | *S. aureus* | SA2 | Isolated from surface wound smear | This study |
|  | *S. aureus* | SA3 | Isolated from nose/throat smear | This study |
|  | *S. aureus* | SA4 | Isolated from urine from kidney fistula | This study |
|  | *S. aureus* | SA5 | Isolated from inner wound smear | This study |
|  | *S. aureus* | SA6 | Isolated from throat smear – MRSA strain | This study |
|  | *S. aureus* | SA7 | Isolated from throat smear | This study |

**Supplementary references**

1. Bremell T, Abdelnour A, Tarkowski A. Histopathological and serological progression of experimental staphylococcus aureus arthritis. *Infect Immun*. 1992; **60**: 2976-2985.
2. Boyle-Vavra S, Daum RS.Community-acquired methicillin-resistant Staphylococcus aureus: the role of Panton-Valentine leukocidin*.Lab Invest*. 2007 Jan;87(1):3-9.
3. Vilacoba, E. and M. Almuzara, Emergence and Spread of Plasmid-Borne tet(B)::ISCR2 in Minocycline-Resistant Acinetobacter baumannii Isolates. *Antimicrobial Agents and Chemotherapy*. 2012. 57(1): p. 651–654.
4. Ramirez, M.S., et al., Genomic analysis of Acinetobacter baumannii A118 by comparison of optical maps: identification of structures related to its susceptibility phenotype. *Antimicrob Agents Chemother*, 2011; 55(4): p. 1520-6.
