# Supplementary material for: Competitive inhibition and mutualistic growth in co-infections: deciphering Staphylococcus aureus–Acinetobacter baumannii interaction dynamics
Source: ISME Commun. 2024 Jun 10;4(1):ycae077. doi: 10.1093/ismeco/ycae077 (PMC11221087; doi:10.1093/ismeco/ycae077)
Supplement: Table_S2_model_parameter_estimation_ranges_ycae077 [file table_s2_model_parameter_estimation_ranges_ycae077.docx]

| **Parameter** | **Range** |
| --- | --- |
| $r_{g}$ | $0.01-2$ |
| $C_{start}$ | $0.01-50$ |
| $C_{end}$ | $0.01-18$ |
| $p$ | $0.01-24$ |
| $g$ | $0.01-5$ |

Supplementary Table 8 Ranges of model parameters used for parameter estimation.
