# Supplementary material for: Competitive inhibition and mutualistic growth in co-infections: deciphering Staphylococcus aureus–Acinetobacter baumannii interaction dynamics
Source: ISME Commun. 2024 Jun 10;4(1):ycae077. doi: 10.1093/ismeco/ycae077 (PMC11221087; doi:10.1093/ismeco/ycae077)
Supplement: Table_S7_statistics_for_Figure_4_ycae077 [file table_s7_statistics_for_figure_4_ycae077.docx]

| **Experiment** | 1h Trypsin | | Proteinase K | | Heat inactivation | |
| --- | --- | --- | --- | --- | --- | --- |
|  | p-value | Cohen’s d | p-value | Cohen’s d | p-value | Cohen’s d |
| A118 + CFCM: USA300 | 0.000267 | -2.491  (large) | 0.000508 | -2.095  (large) | 0.089928 | -0.782  (medium) |
| USA300 + CFCM: A118 | 0.089192 | -0,666 (medium) | 0.089192 | -1.31  (large) | 0.089192 | 1.52  (large) |

Supplementary Table 6 Statistical data for survival ratios $\boldsymbol{S}\boldsymbol{R}_{\boldsymbol{TSB}}^{\boldsymbol{otherCFCM}}$shown in Figure 4. Significance of survival ratios in treated CFCM was tested in comparison to survival ratios in CFCM without treatment using a paired t-test and false discovery rate was considered using the Benjamini-Hochberg correction. (Significance levels: ns.: p>0.05, *: p < 0.05; **: p < 0.01; ***: p < 0.001; ****: p < 0.0001.) For the effect size Cohen’s d was calculated.
