# Supplementary material for: Competitive inhibition and mutualistic growth in co-infections: deciphering Staphylococcus aureus–Acinetobacter baumannii interaction dynamics
Source: ISME Commun. 2024 Jun 10;4(1):ycae077. doi: 10.1093/ismeco/ycae077 (PMC11221087; doi:10.1093/ismeco/ycae077)
Supplement: Table_S10_statistics_for_Figure_5_ycae077 [file table_s10_statistics_for_figure_5_ycae077.docx]

| **Experiment** | **Δagr** | | **Δpsmα** | | **Δpsmβ** | |
| --- | --- | --- | --- | --- | --- | --- |
|  | p-value | Cohen’s d | p-value | Cohen’s d | p-value | Cohen’s d |
| A118 + CFCM: LS1 | 0.006221 | -3.548  (large) | 0.0299475 | -0.867  (large) | 0.000504 | -0.545  (medium) |
| A118 + CFCM: USA300 | 0.007839 | -3.078  (large) | 0.007839 | -2.29  (large) | 0.089754 | 0.707  (medium) |
| A42 + CFCM: LS1 | 0.003986 | -0.587  (medium) | 0.005235 | -1.727  (large) | 0.003987 | -0.935  (large) |
| A42 + CFCM: USA300 | 0.473127 | -0.318  (small) | 0.2241914 | -0.574  (medium) | 0.961724 | -0.035  (negligible) |
| LS1 + CFCM: A118 | 0.346275 | 0.241  (small) | 0.131539 | -0.604 (medium) | 0.227149 | 0.544 (medium) |
| USA300 + CFCM: A118 | 0.306944 | -1.720 (large) | 0.505417 | -0.687 (medium) | 0.505417 | -0.32  (small) |

Supplementary Table 7 Statistical data for survival ratios $SR_{TSB}^{otherCFCM}$shown in Figure 5. Significance of survival ratios in treated CFCM was tested in comparison to survival ratios in CFCM without treatment using a paired t-test and false discovery rate was considered using the Benjamini-Hochberg correction. (Significance levels: ns.: p>0.05, *: p < 0.05; **: p < 0.01; ***: p < 0.001; ****: p < 0.0001.) For the effect size Cohen’s d was calculated.
