# Supplementary material for: Competitive inhibition and mutualistic growth in co-infections: deciphering Staphylococcus aureus–Acinetobacter baumannii interaction dynamics
Source: ISME Commun. 2024 Jun 10;4(1):ycae077. doi: 10.1093/ismeco/ycae077 (PMC11221087; doi:10.1093/ismeco/ycae077)
Supplement: Suppl_Mat_Proteomics_ycae077 [file suppl_mat_proteomics_ycae077.pdf]

DIA Quantification Proteomics Report

2021/7/22

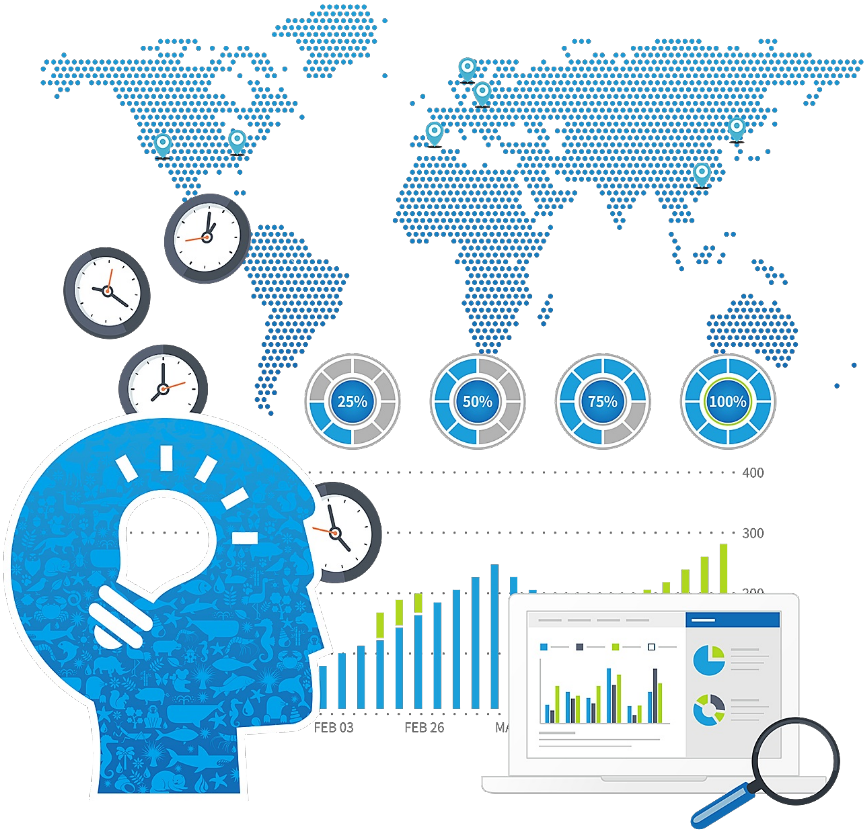

@2021 BGI All Rights Reserved

Table of Contents

Results 2
- 1 Project Overview and Quality Control Analysis 2
- 2 DDA Spectral Library 3
- 3 Protein Quantification 5
- 4 Function Annotation 8
- 5 Differential Proteins Function Annotation 12

Methods 17
- 1 Pipeline Introduction 17
- 2 Experimental Pipeline 18
- 3 Bioinformatic Analysis Pipeline 20

Help 23
- 1 Protein Sequence: FASTA Format 23
- 2 File Format Description of all_peptideSummary.xls 23
- 3 File Format Description of proteinGroups_recalibration.txt 24
- 4 File Format Description of annotation_allprotein.xls 24
- 5 File Format Description of XX2-VS-XX1.All.xls 25
- 6 File Format Description of dia-proteinSummary.xls 26
- 7 File Format Description of dia-peptideSummary.xls 26
- 8 How to Read Report of Clustering Analysis 27
- 9 File Format Description of Time Series Analysis Results 28
- 10 How to Read Report of GO Functional Annotation and Enrichment Results 29
- 11 How to Read Report of COG/KOG Functional Annotation Results 30
- 12 How to Read Report of Pathway Functional Annotation and Enrichment Results 31
- 13 How to Read Report of Protein-protein Interaction Results 33
- 14 How to Read Report of Subcellular Localization 34
- 15 NR Database 35
- 16 Swiss-Prot Database 35
- 17 COG/KOG Database 35
- 18 GO Database 35
- 19 KEGG Database 36

FAQs 36

References 36

BGI Co., Ltd. 400-706-6615 ©2021 BGI All Rights Reserved.

Results

1 Project Overview and Quality Control Analysis

1.1 Overview

In this project, Fusion Lumos (Thermo Fisher Scientific, San Jose, CA) was used to acquire mass spectrometry (MS) data for 12 samples in Data Independent Acquisition (DIA) mode, 12468 peptide and 1600 protein were quantitated. Quantification of peptides and proteins was performed using MSstats software packages. The quantitative statistics of each sample are as follows:

Table 1 Overview of quantitative results for each sample （See all)

Name	Peptide number	Protein number
A118_24h_11	2145	489
A118_24h_12	2010	476
A118_3h_5	853	222
A118_3h_6	467	139
Medium_24h_7	105	56
Medium_24h_8	78	48
Medium_3h_1	112	64
Medium_3h_2	109	70
USA300_24h_9	6605	1175
USA300_24h_10	7226	1183

1.2 Quality control

The quality of the DIA data was evaluated based on intra-group coefficient of variation (CV), principal component analysis (PCA), and quantitative correlation of samples. When the sample size is large, quality control (QC) samples, which are generally a mixture of all samples, are inserted intermittently between the continuous original samples. Thus, experimental conditions can be evaluated by the following QC analysis to ensure stability and repeatability of the experiment.

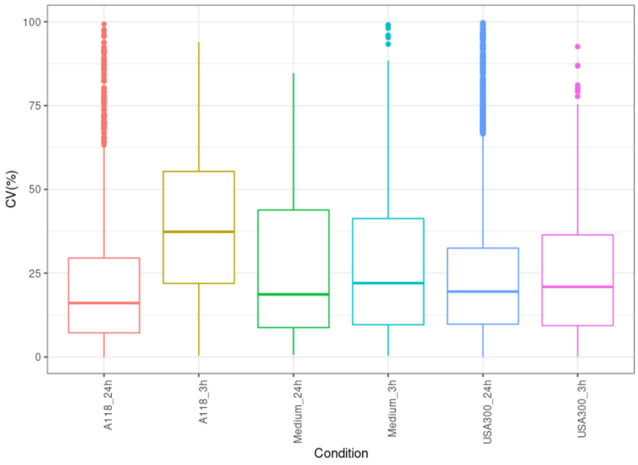

Figure 1 CV distribution.

This analysis was used to calculate intra-group CV of different sample groups. The X-axis denotes the sample group and the Y-axis denotes the corresponding CV. (When the sample size in project is large, there is a QC group to be used to evaluate stability and repeatability of the experiment).

BGI Co., Ltd. 400-706-6615 ©2021 BGI All Rights Reserved.

In this project, Pearson correlation coefficient of all protein expression between every two samples was calculated to demonstrate correlation of protein quantification between samples and was represented as a heat map as follows:

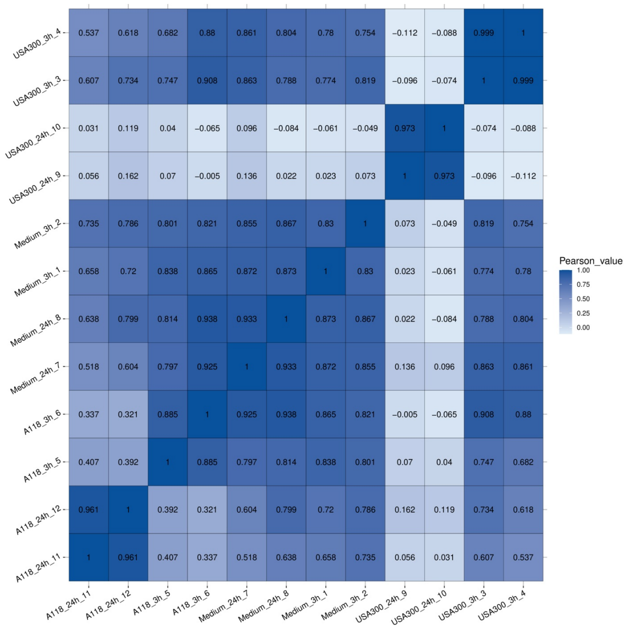

Figure 2 Heat map of sample correlation analysis.

Both X and Y axes represent samples. The color represents the correlation coefficient (the deeper color represents the higher correlation; the lighter color represents the lower correlation).

1.3 Identification and quantification detail table

The following are the lists of peptides and proteins for each sample. For the meaning of each column in the list, please refer to the attached Help documentations.

Peptide Quantification List: [dia-peptideSummary.xls]

Protein Quantification List: [dia-proteinSummary.xls]

Protein Identification List: [annotation_allprotein.xls]

2 DDA Spectral Library

The samples of interest went through mass spectrometry data collection in data dependent acquisition (DDA) mode. MaxQuant was then used to carry out database search identification process and obtain all detectable non-redundant high-quality MS/MS spectral information as DIA spectral library, which contains fragment ion intensity and retention time describing the peak characteristics of the peptide, for quantification. The statistics of peptides and proteins in the spectral library are listed as follows:

Table 2 DDA spectral library statistics (Download)

©2021 BGI All Rights Reserved.

Item	Number
Peptide	18970
Protein	2831

The following figures are the basic statistics of the DDA identification results, which are unique peptide distribution, protein mass distribution and protein coverage distribution respectively.

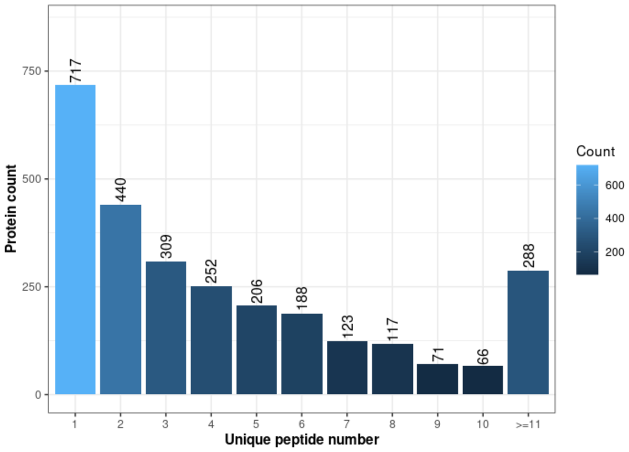

Figure 3 Unique peptide distribution.

The X-axis is the number of unique peptides for each protein, and the Y-axis is the number of proteins.

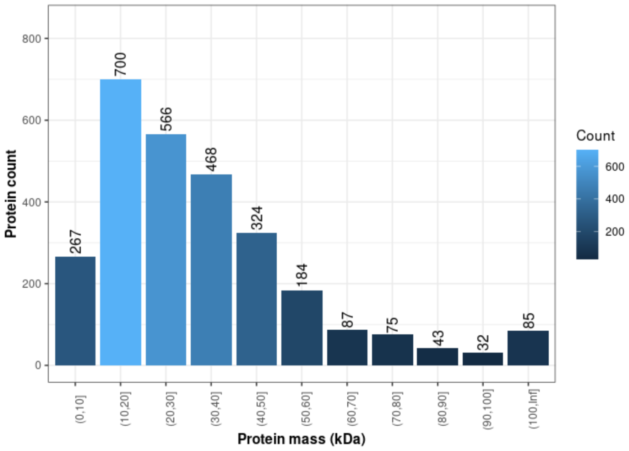

Figure 4 Protein mass distribution.

The X-axis is protein mass interval (Kilodalton), and the Y-axis is the number of proteins.

BGI Co., Ltd. 400-706-6615 ©2021 BGI All Rights Reserved.

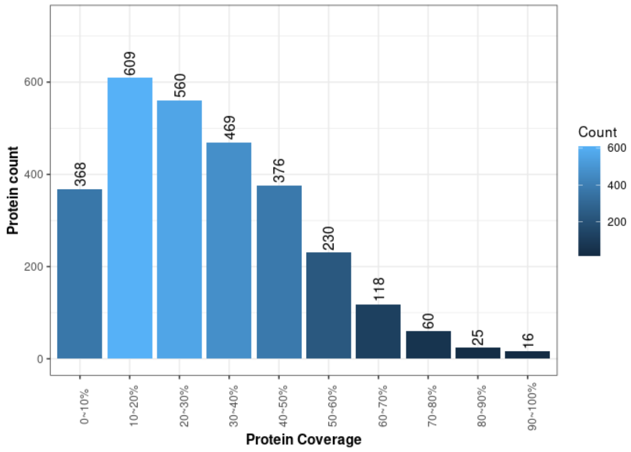

Figure 5 Protein coverage distribution.

The X-axis is coverage percentage interval, and the Y-axis is the number of proteins.

The following lists are peptide list and protein list after DDA data are analysed by MaxQuant. For the meaning of each column in the list, please refer to the attached Help documentation.

Peptide list: [all-peptideSummary]

Protein list: [proteinGroups_recalibration]

3 Protein Quantification

3.1 Quantitative differential analysis

In this project, MSstats software package was applied to intra-system error correction, normalization for each sample. Then based on the predefined comparison groups and the linear mixed effect model, the significance of differentially expressed proteins (DEPs) was evaluated. Two filtration criteria (Fold change > 1.5 and Pvalue < 0.05) were used to get significant differential proteins. The statistics of the differences between the comparison groups are as follows:

Table 3 Statistics of differentially expressed proteins (Download)

Comparsion group	Down-regulated	Non-regulated	Up-regulated
A118_24h-VS-A118_3h	17	131	41
A118_24h-VS-Medium_24h	2	37	13
A118_3h-VS-Medium_3h	1	50	10
USA300_24h-VS-A118_24h	27	83	26
USA300_24h-VS-Medium_24h	14	26	16
USA300_24h-VS-USA300_3h	23	71	108
USA300_3h-VS-A118_3h	1	70	5
USA300_3h-VS-Medium_3h	1	42	19

 ©2021 BGI All Rights Reserved.

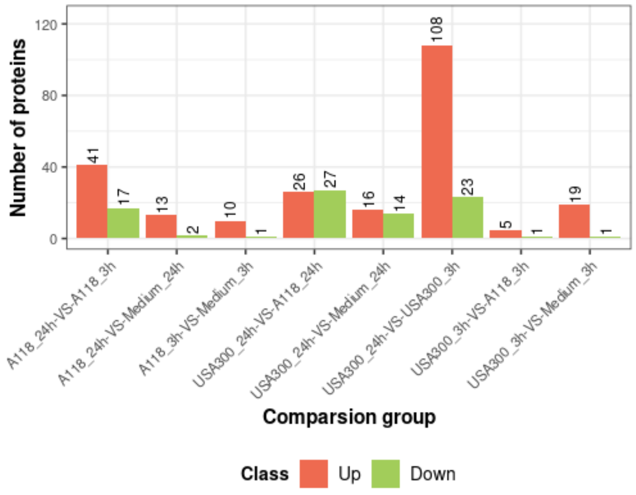

Figure 6 Bar chart of differentially expressed proteins.

The following is a volcano plot that helps to filter differentially expressed proteins visually:

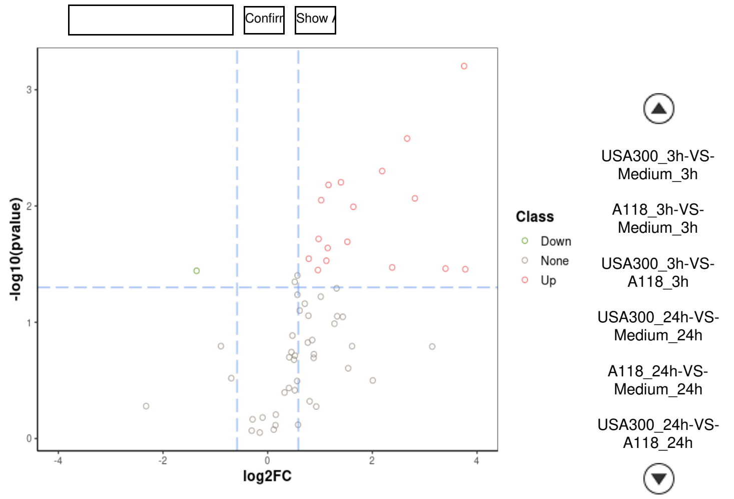

Figure 7 Volcano plot.

The X-axis of the graph is the protein fold change (log 2), and the Y-axis is the corresponding -log10 (P-value). In the figure, the red dot indicates significantly up-regulated proteins, the green dot indicates significantly down-regulated proteins, and the grey dot indicates proteins without significant change.

The following is a table of quantitative differential results for each comparison group.

USA300_3h-VS-Medium_3h quantitative differential result: [USA300_3h-VS-Medium_3h.All.xls] [USA300_3h-VS-Medium_3h.Up.xls] [USA300_3h-VS-Medium_3h.Down.xls]

A118_3h-VS-Medium_3h quantitative differential result: [A118_3h-VS-Medium_3h.All.xls] [A118_3h-VS-Medium_3h.Up.xls] [A118_3h-VS-Medium_3h.Down.xls]

USA300_3h-VS-A118_3h quantitative differential result: [USA300_3h-VS-A118_3h.All.xls] [USA300_3h-VS-A118_3h.Up.xls] [USA300_3h-VS-A118_3h.Down.xls]

USA300_24h-VS-Medium_24h quantitative differential result: [USA300_24h-VS-

 ©2021 BGI All Rights Reserved.

Medium_24h.All.xls] [USA300_24h-VS-Medium_24h.Up.xls] [USA300_24h-VS-Medium_24h.Down.xls]

A118_24h-VS-Medium_24h quantitative differential result: [A118_24h-VS-Medium_24h.All.xls] [A118_24h-VS-Medium_24h.Up.xls] [A118_24h-VS-Medium_24h.Down.xls]

USA300_24h-VS-A118_24h quantitative differential result: [USA300_24h-VS-A118_24h.All.xls] [USA300_24h-VS-A118_24h.Up.xls] [USA300_24h-VS-A118_24h.Down.xls]

USA300_24h-VS-USA300_3h quantitative differential result: [USA300_24h-VS-USA300_3h.All.xls] [USA300_24h-VS-USA300_3h.Up.xls] [USA300_24h-VS-USA300_3h.Down.xls]

A118_24h-VS-A118_3h quantitative differential result: [A118_24h-VS-A118_3h.All.xls] [A118_24h-VS-A118_3h.Up.xls] [A118_24h-VS-A118_3h.Down.xls]

3.2 Principal component analysis

Principal component analysis (PCA) is a method of dimension reduction that combines multiple variables to a new set of integrated variables, and then selects several (usually 2-3) to represent as much original information as possible, thus to achieve the purpose of dimension reduction. PCA is mainly used to observe the trend of separation between groups in the experimental model, and whether there are exceptional value points, and reflect the inter- and intra- group variations from the original data.

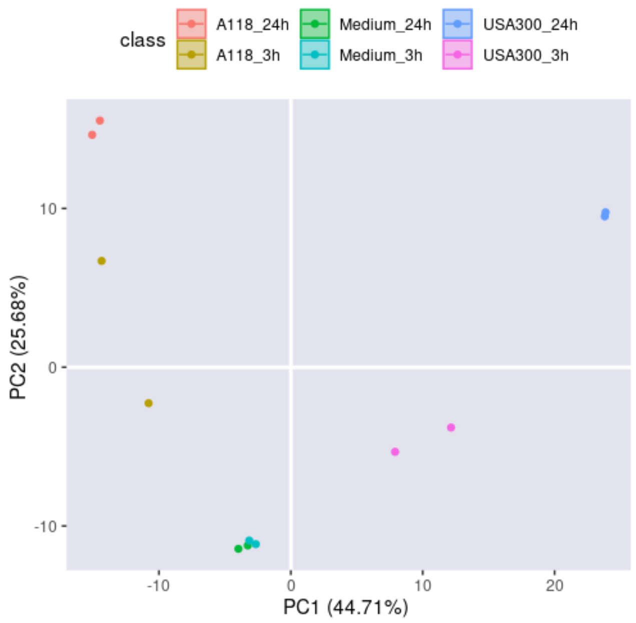

Figure 8 Principal component analysis.

The X-axis is the first principal component and the Y-axis is the second principal component.

PCA result of protein level:[pca.protein.png]

3.3 Cluster analysis

BGI Co., Ltd. 400-706-6615 ©2021 BGI All Rights Reserved.

As an effective data analysis tool, cluster analysis has been widely used in the fields such as image processing, information retrieval, data mining. Cluster analysis is also widely used in the gene and protein expression data analysis, including discovering the unknown function of genes or proteins by clustering genes or proteins, automatically classifying pathological features or experimental conditions by clustering samples, finding regulatory genes or protein cluster under certain conditions through two-way clustering. We used the Euclidean distance and Hierarchical Cluster to cluster the differential proteins.

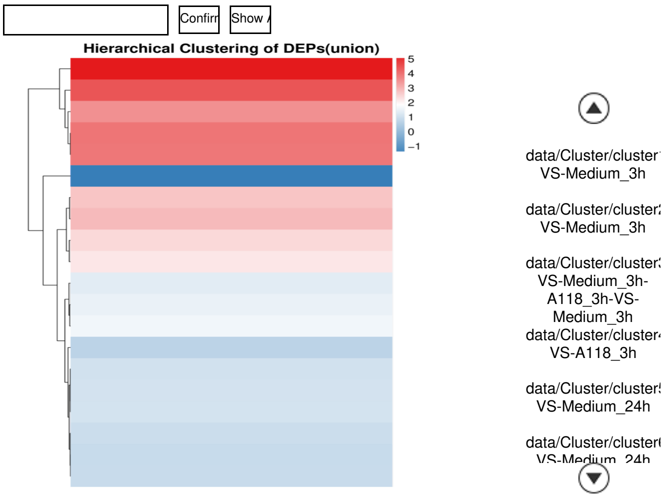

Figure 9 Differential protein clustering.

Red represents significantly up-regulated proteins, blue represents significantly down-regulated proteins, and white represents proteins without significant change.

Cluster1: [cluster USA300_3h-VS-Medium_3h]

Cluster2: [cluster A118_3h-VS-Medium_3h]

Cluster3: [cluster USA300_3h-VS-Medium_3h,A118_3h-VS-Medium_3h]

Cluster4: [cluster USA300_3h-VS-A118_3h]

Cluster5: [cluster USA300_24h-VS-Medium_24h]

Cluster6: [cluster A118_24h-VS-Medium_24h]

Cluster7: [cluster USA300_24h-VS-Medium_24h,A118_24h-VS-Medium_24h]

Cluster8: [cluster USA300_24h-VS-A118_24h]

Cluster9: [cluster USA300_24h-VS-USA300_3h]

Cluster10: [cluster A118_24h-VS-A118_3h]

Cluster11: [cluster USA300_3h-VS-Medium_3h,A118_3h-VS-Medium_3h,USA300_24h-VS-Medium_24h,A118_24h-VS-Medium_24h]

4 Function Annotation

4.1 GO annotation

 ©2021 BGI All Rights Reserved.

Gene Ontology (GO) is an international standard gene function classification system that provides a timely updated standard vocabulary (Controlled Vocabulary) to comprehensively describe the properties of genes and gene products in organisms. GO includes in total three ontologies, describing Molecular Function, Cellular Component, and Biological Process of genes.

See the website for details: http://www.geneontology.org.

We carried out a GO function annotation analysis to all identified proteins, and the results include two parts: protein2go and go2protein.

protein2go:For each protein, a list of IDs and all corresponding GO functions are given.

go2protein:For the GO entries involved in the three ontologies (cellular component, biological process, molecular function), the IDs and the number of all the corresponding proteins are listed, and a statistical chart is made, and the GO entries without the corresponding proteins are excluded.

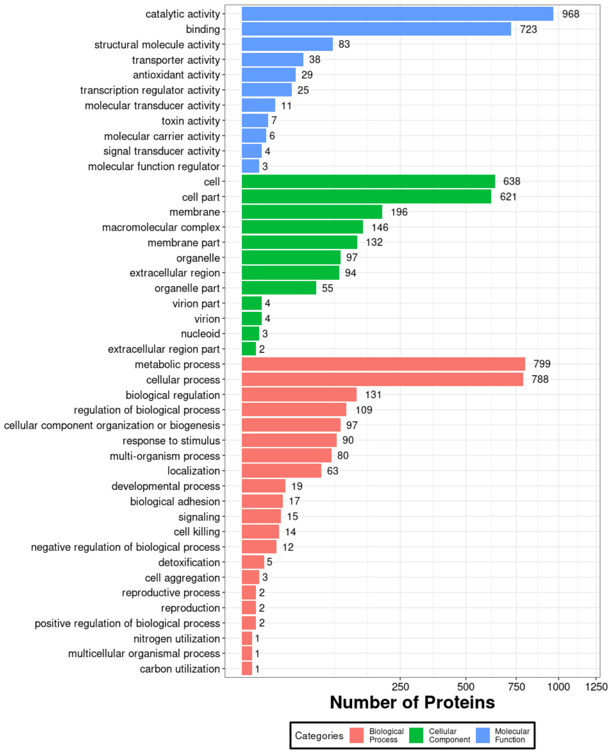

Figure 10 GO function annotation.

The GO classification map shows the distribution of the entries involved in the three ontologies, and the different colors are labeled as the individual entries involved in the three ontologies.

GO Annotation Result: [Staphylococcus_aureus_and_Acinetobacter_baumannii_GO.zip]

4.2 COG annotation

COG (Cluster of Orthologous Groups of proteins) is a database for orthologous classification of proteins. The proteins that make up each COG are assumed to be derived from an ancestral protein and are orthologss or paralogs proteins. Orthologs are proteins that evolved from vertical pedigrees (species formation) from different species and are specifically retained and the same function of the

 ©2021 BGI All Rights Reserved.

original protein. Paralogs are proteins that are derived from gene duplication in certain species and may evolve new and previously relevant functions. The analysis compares the identified proteins with the COG database, predicts the possible functions of these proteins and performs functional classification statistics.

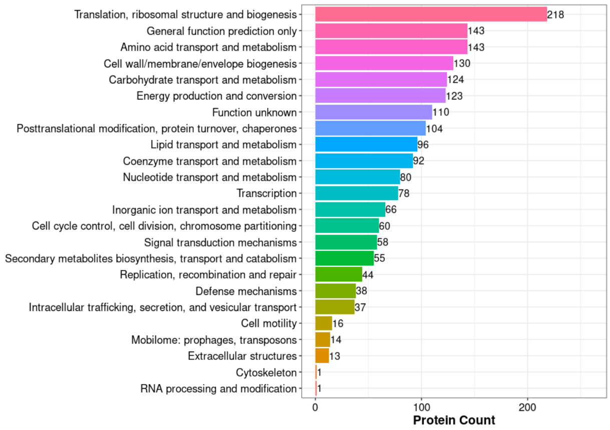

Figure 11 Bar plot of the COG analysis.

Y-axis displays the COG entries, X-axis displays the corresponding protein count for each entry. The figure illustrates the protein number with different functions in the sample.

COG annotation result: [Staphylococcus_aureus_and_Acinetobacter_baumannii_COG.zip]

4.3 Pathway annotation

In vivo, DEPs coordinate and carry out their biological behavior, and Pathway-based analysis helps to further understand their biological functions. KEGG is the Kyoto Encyclopedia of Genes and Genomes, founded in 1995 by the Kanehisa Laboratory of the Bioinformatics Center of Kyoto University, Japan, which is one of the most commonly used bioinformatics databases in the world and is known for understanding the advanced functions and utility libraries of biological systems. The KEGG PATHWAY database is the core of the KEGG database. Its distinctive feature includes powerful graphics functionality. It uses graphics instead of verbose words to introduce metabolic pathways and their relationships, so that researchers can have an intuitive and comprehensive understanding towards different pathways. References: http://www.genome.jp/kegg/pathway.html

BGI Co., Ltd. 400-706-6615 ©2021 BGI All Rights Reserved.

1. Human

#	Pathway	Proteins with pathway annotation (6877)	Pathway ID
1	Metabolic pathways	973 (14.15%)	ko01100
2	Focal adhesion	280 (4.07%)	ko04510
3	Epstein-Barr virus infection	280 (4.07%)	ko05169
4	Pathways in cancer	268 (3.9%)	ko05200
5	Regulation of actin cytoskeleton	241 (3.5%)	ko04810
6	Endocytosis	236 (3.43%)	ko04144
7	Phagosome	231 (3.36%)	ko04145
8	HTLV-I infection	211 (3.07%)	ko05166
9	Tuberculosis	209 (3.04%)	ko05152
10	MAPK signaling pathway	207 (3.01%)	ko04010
11	Influenza A	190 (2.76%)	ko05164
12	Tight junction	185 (2.69%)	ko04530
13	Transcriptional misregulation in cancer	184 (2.68%)	ko05202
14	Huntington's disease	177 (2.57%)	ko05016
15	Fc gamma R-mediated phagocytosis	176 (2.56%)	ko04666
16	RNA transport	175 (2.54%)	ko03013
17	Herpes simplex infection	171 (2.49%)	ko05168
18	Amoebiasis	167 (2.43%)	ko05146
19	Spliceosome	163 (2.37%)	ko03040
20	Protein processing in endoplasmic reticulum	162 (2.36%)	ko04141
21	Leukocyte transendothelial migration	157 (2.28%)	ko04670
22	Viral myocarditis	157 (2.28%)	ko05416
23	Alzheimer's disease	155 (2.25%)	ko05010
24	Dilated cardiomyopathy	155 (2.25%)	ko05414
25	Measles	152 (2.21%)	ko05162

Figure 12 Pathway annotation result.

This picture is a screenshot of the Pathway annotation result. The actual results are subject to the following compression package.

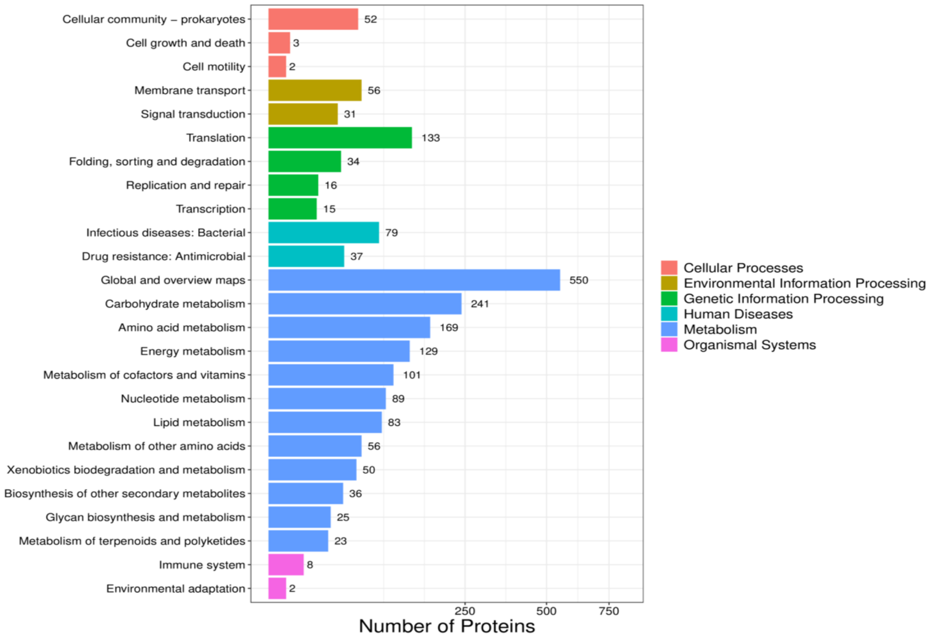

Figure 13 Pathway annotation histogram .

The X-axis represents the number of protein annotations and the Y-axis represents the KEGG functional classification. The KEGG metabolic pathway contains seven branches: Cellular Processes, Environmental Information Processing, Genetic Information Processing, Human Diseases (animals only), Metabolism, Organic Systems, Drug Development.

Pathway Annotation Result: [Staphylococcus_aureus_and_Acinetobacter_baumannii_Pathway.zip]

©2021 BGI All Rights Reserved.

5 Differential Proteins Function Annotation

5.1 GO enrichment analysis

For proteins with significant up/down-regulation, GO enrichment analysis provides GO entries that are significantly enriched for differential proteins, revealing the biological functions on which researchers mostly focus. This result is usually a significantly enriched GO entries with P-value < 0.05.

Result Table

Terms from the Component Ontology with p-value as good or better than 1

Gene Ontology term	Cluster frequency	Protein frequency of use	P-value
ribonucleoprotein complex	12 out of 36 genes, 33.3%	90 out of 766 genes, 11.7%	0.0003887879
intracellular	30 out of 36 genes, 83.3%	511 out of 766 genes, 66.7%	0.01947108
chromatin	2 out of 36 genes, 5.6%	6 out of 766 genes, 0.8%	0.02860762
intracellular part	29 out of 36 genes, 80.6%	503 out of 766 genes, 65.7%	0.03624558
macromolecular complex	14 out of 36 genes, 38.9%	197 out of 766 genes, 25.7%	0.05277838
cell	32 out of 36 genes, 88.9%	592 out of 766 genes, 77.3%	0.05989476
cell part	32 out of 36 genes, 88.9%	592 out of 766 genes, 77.3%	0.05989476
non-membrane-bounded organelle	5 out of 36 genes, 13.9%	47 out of 766 genes, 6.1%	0.06258606
intracellular non-membrane-bounded organelle	5 out of 36 genes, 13.9%	47 out of 766 genes, 6.1%	0.06258606
endoplasmic reticulum	2 out of 36 genes, 5.6%	9 out of 766 genes, 1.2%	0.06281128
chromosomal part	2 out of 36 genes, 5.6%	9 out of 766 genes, 1.2%	0.06281128
chromosome	2 out of 36 genes, 5.6%	11 out of 766 genes, 1.4%	0.09047561
cytoplasmic part	5 out of 36 genes, 13.9%	80 out of 766 genes, 10.4%	0.3197551
cytoplasm	5 out of 36 genes, 13.9%	81 out of 766 genes, 10.6%	0.3295799

Figure 14 Demonstration of GO enrichment analysis results.

This picture is a screenshot of the GO enrichment results demo. The actual results can be found in the following compression package.

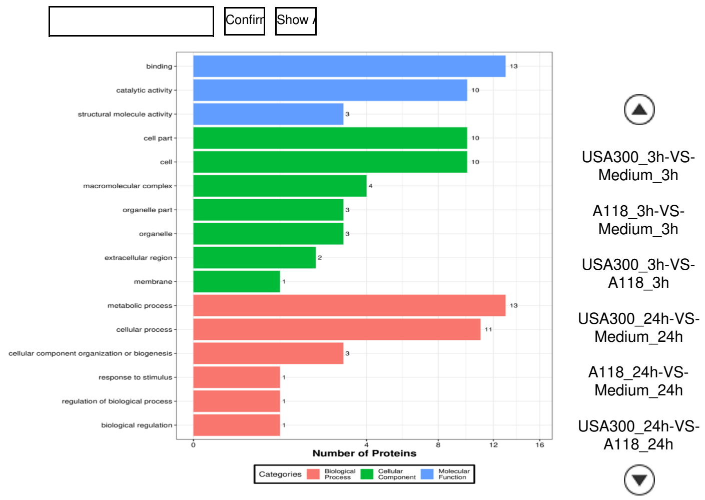

Figure 15 Differential protein GO function classification.

The X-axis represents the number of differential proteins and the Y-axis represents the GO annotation entry.

BGI Co., Ltd. 400-706-6615 ©2021 BGI All Rights Reserved.

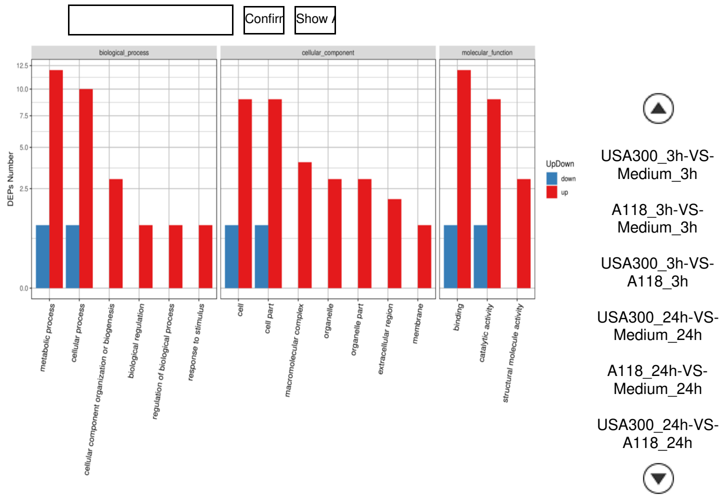

Figure 16 Up or down regulation of differential proteins in GO function classification.

The X-axis represents the GO annotation entry, and the Y-axis represents the number of differential proteins with up or down regulation.

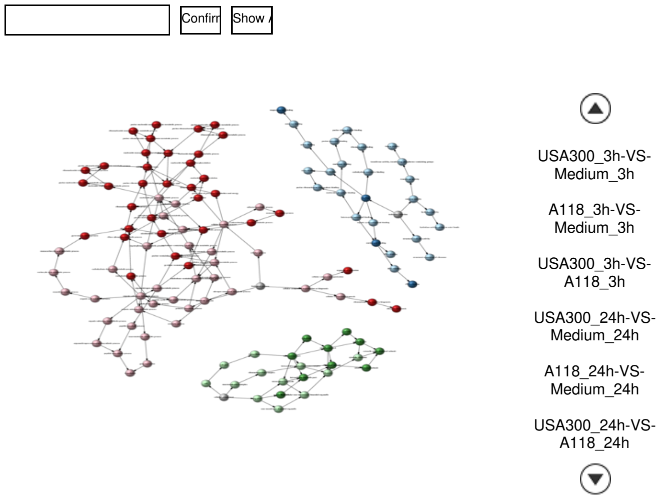

Figure 17 GO term relationship network.

Each node represents a GO term, with different colors representing different functional classification, red for biological processes, green for cellular component, and blue for molecular function. Dark color indicates significantly enriched GO term, light color indicates insignificant GO term, and grey indicate no enriched GO term. Similar to the directed acyclic graph, the relationship between the various GO terms can be seen, where the solid arrow indicates the inclusion relationship, the dotted arrow indicates the regulation relationship, the red dotted line indicates up regulation, and the green dotted line indicates down regulation. (You can freely choose or adjust the location of the gene from the web page.)

GO Enrichment Result: [GO_Enrichment.zip]

5.2 Pathway enrichment analysis

The following figure shows a screenshot of the pathway enrichment analysis result web page. In the list, P-value<0.05 is the threshold for metabolic pathway with significant enrichment of differential proteins.

BGI Co., Ltd. 400-706-6615 ©2021 BGI All Rights Reserved.

1. F-VS-E

#	Pathway	Diff Proteins with pathway annotation (273)	All Proteins with pathway annotation (6877)	Pvalue	Pathway ID
1	Primary immunodeficiency	26 (9.52%)	126 (1.83%)	2.054643e-12	ko05340
2	Staphylococcus aureus infection	18 (6.59%)	139 (2.02%)	8.330702e-06	ko05150
3	Cytokine-cytokine receptor interaction	10 (3.66%)	46 (0.67%)	9.464853e-06	ko04060
4	Dilated cardiomyopathy	19 (6.96%)	155 (2.25%)	1.048952e-05	ko05414
5	TGF-beta signaling pathway	11 (4.03%)	59 (0.86%)	1.616117e-05	ko04350
6	Complement and coagulation cascades	14 (5.13%)	97 (1.41%)	2.500151e-05	ko04610
7	African trypanosomiasis	13 (4.76%)	87 (1.27%)	3.351947e-05	ko05143
8	ECM-receptor interaction	16 (5.86%)	130 (1.89%)	5.036522e-05	ko04512
9	Intestinal immune network for IgA production	13 (4.76%)	92 (1.34%)	6.122737e-05	ko04672
10	Amoebiasis	17 (6.23%)	167 (2.43%)	0.0003154249	ko05146
11	Hematopoietic cell lineage	14 (5.13%)	132 (1.92%)	0.000704302	ko04640
12	p53 signaling pathway	8 (2.93%)	57 (0.83%)	0.001694385	ko04115
13	Rheumatoid arthritis	12 (4.4%)	114 (1.66%)	0.00178888	ko05323
14	Systemic lupus erythematosus	14 (5.13%)	147 (2.14%)	0.002012887	ko05322
15	Autoimmune thyroid disease	11 (4.03%)	104 (1.51%)	0.002647169	ko05320
16	PPAR signaling pathway	8 (2.93%)	68 (0.99%)	0.00520706	ko03320
17	NF-kappa B signaling pathway	12 (4.4%)	133 (1.93%)	0.006378992	ko04064
18	Asthma	9 (3.3%)	87 (1.27%)	0.00731645	ko05310
19	Butirosin and neomycin biosynthesis	2 (0.73%)	4 (0.06%)	0.008934195	ko00524
20	Hypertrophic cardiomyopathy (HCM)	10 (3.66%)	106 (1.54%)	0.009122396	ko05410
21	Protein digestion and absorption	9 (3.3%)	93 (1.35%)	0.01117142	ko04974
22	Bladder cancer	5 (1.83%)	35 (0.51%)	0.01161771	ko05219
23	B cell receptor signaling pathway	12 (4.4%)	144 (2.09%)	0.01173280	ko04662
24	Fc epsilon RI signaling pathway	11 (4.03%)	127 (1.85%)	0.01185026	ko04664
25	Transcriptional misregulation in cancer	14 (5.13%)	184 (2.68%)	0.01442243	ko05202
26	Natural killer cell mediated cytotoxicity	12 (4.4%)	149 (2.17%)	0.01508876	ko04650
27	Focal adhesion	19 (6.96%)	280 (4.07%)	0.01531133	ko04510
28	Tuberculosis	15 (5.49%)	209 (3.04%)	0.01882283	ko05152
29	Allograft rejection	9 (3.3%)	102 (1.48%)	0.01956793	ko05330
30	Malaria	5 (1.83%)	40 (0.58%)	0.02006485	ko05144

Figure 18 Pathway enrichment analysis results demo.

This figure is a screenshot of the pathway enrichment demo. The actual results can be found in results directory.

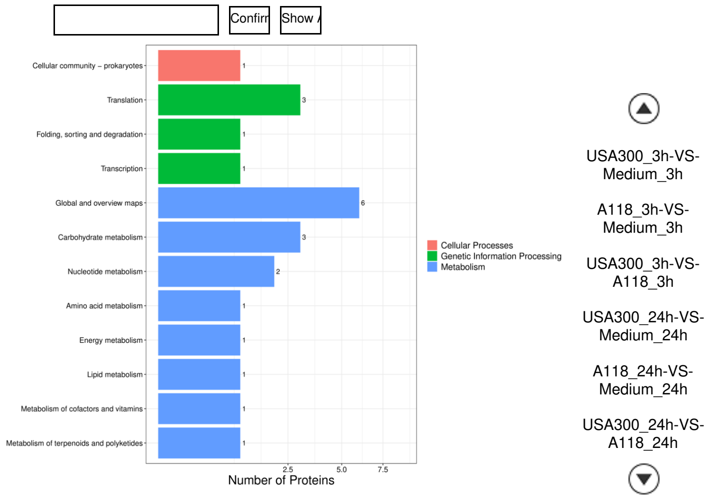

Figure 19 Differential protein pathway classification.

The X-axis represents the number of differential proteins, and the Y-axis represents the pathway annotation entry.

BGI Co., Ltd.   400-706-6615   ©2021 BGI All Rights Reserved.

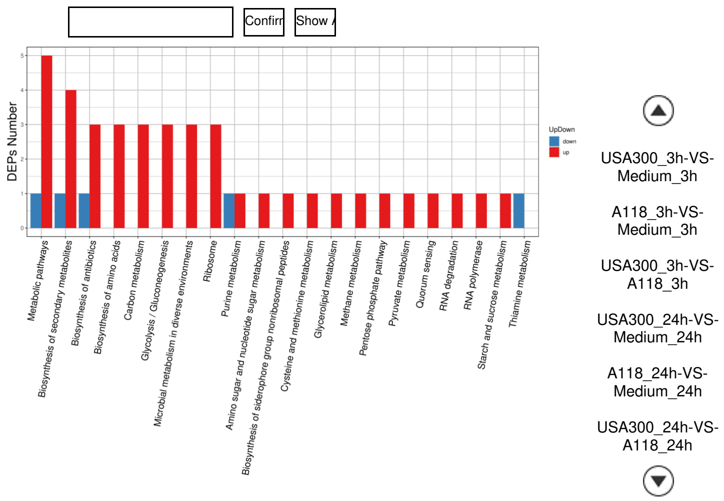

Figure 20 Up and down regulation differential protein pathway classification.

The X-axis represents the pathway annotation entry, and the Y-axis represents the number of differential protein with up or down regulation..

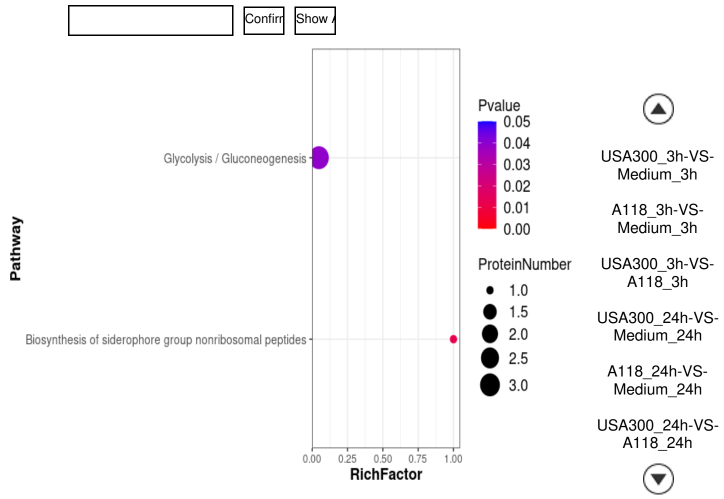

Figure 21 Significantly enriched pathway.

This figure shows the metabolic pathway in which the differential proteins are significantly enriched. The X-axis is enrichment factor (RichFactor) which represents the number of differential proteins annotated to the pathway divided by all the proteins identified in the pathway. The larger the value, indicating the greater the proportion of differential proteins annotated to the pathway. The size of the circle represents the number of differential proteins annotated to the pathway.

 ©2021 BGI All Rights Reserved.

Confirm Show

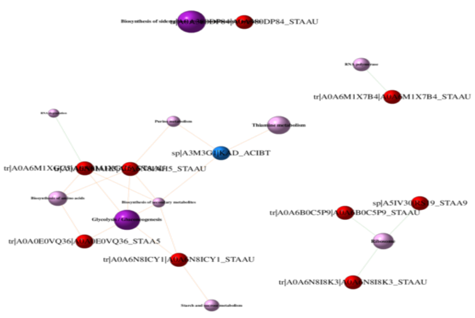

USA300_3h-VS-Medium_3h

A118_3h-VS-Medium_3h

USA300_3h-VS-A118_3h

USA300_24h-VS-Medium_24h

A118_24h-VS-Medium_24h

USA300_24h-VS-A118_24h

Figure 22 Pathway relationship networks.

The red dots represent differential proteins with up regulation and the blue dots represent the differential proteins with down regulation. The purple circles represent top ten enriched pathway, darker purple indicates significant enrichment, lighter purple indicates insignificant enriched, while larger area indicates higher level of enrichment. Different color lines represent different classifications of pathway, red represents Cellular Processes, blue represents Environmental Information Processing, green represents Genetic Information Processing, purple represents Human Disease (animal only), orange represents Metabolism, yellow represents Organic Systems and brown represents Drug Development. (You can freely choose or adjust the layout in the web page.)

Pathway Enrichment Result: [Pathway_Enrichment.zip]

5.3 COG annotation of DEPs

For DEPs, their annotated COG terms were extracted, and represented as bar plots accordingly. Thus, we can easily visualize the functional classifications of DEPs.

Confirm Show

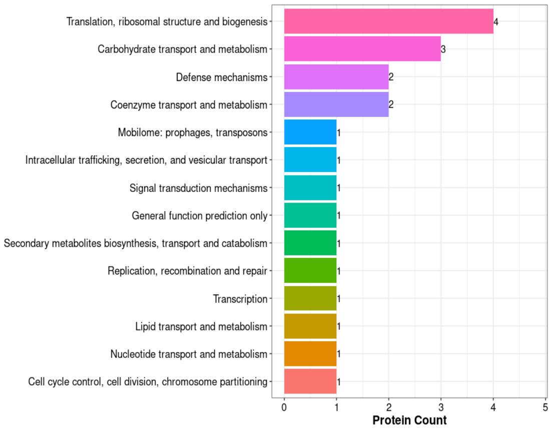

USA300_3h-VS-Medium_3h

A118_3h-VS-Medium_3h

USA300_3h-VS-A118_3h

USA300_24h-VS-Medium_24h

A118_24h-VS-Medium_24h

USA300_24h-VS-A118_24h

Figure 23 COG annotation of DEPs.

X-axis displays DEPs count, Y-axis displays COG terms.

COG Annotation Results:[COG.zip]

BGI Co., Ltd. 400-706-6615 ©2021 BGI All Rights Reserved.

5.4 Protein-Protein interaction analysis of DEPs

Proteins often carry out a specific function after combining into a complex though protein-protein interaction. STRING[1] is a database of known and predicted protein-protein interactions(PPI). PPI analysis of DEPs was done by searching STRING PPI database, and top 100 interactions with confidence was used to construct the interaction map, as below:

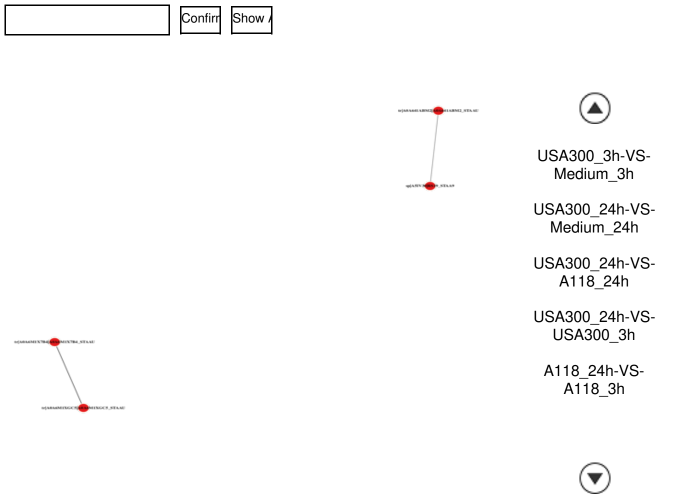

Figure 24 PPI network of DEPs.

Red nodes represent proteins with up regulation; blue nodes represent proteins with down regulation. The size of the circle indicates the density of relationship.

PPI Analysis Results: [PPI.zip]

Methods

1 Pipeline Introduction

This project was analyzed using next generation label-free quantitative proteomics technology. In data independent acquisition (DIA) mode, it can deliver unprecedented proteomic coverage while enabling accurate and highly repeatable quantification for large amounts of proteins per sample. The DIA analysis pipeline provides an ideal differential proteomic analysis or a proteomic quantification platform for large amounts of samples.

The DIA analysis pipeline is based on three essential steps:

1) Spectral library construction: A spectral library collects all detectable non-redundant, high-quality peptide information (MS/MS spectra) of the sample that can be used as a peptide identification template for subsequent data analysis. It contains fragment ion intensity and retention time that characterize the peptide spectrum. The spectral library is constructed from samples of interest by using data dependent acquisition (DDA) technique.

2) Large sample data acquisition in DIA mode: Data independent acquisition (DIA, also called SWATH) mode utilizes the latest high-resolution mass spectrometer to simultaneously acquire peptide ion characteristics in mass and retention time space. Compared to traditional technique of extracting single ion for fragmentation analysis, in DIA mode the mass spectrometer is set to a wide precursor ion

BGI Co., Ltd. 400-706-6615 ©2021 BGI All Rights Reserved.

window to collect product ions in turn. Thus complete collection of all detectable protein peak information in the sample and high-reproducible analysis of large number of samples is achieved.

3) Data analysis: Identificaton and quantification of peptides and proteins were obtained from DDA spectral library by deconvolution of the DIA data. MSstats software package was used to perform differential analysis, followed by functional analysis of the differential proteins.

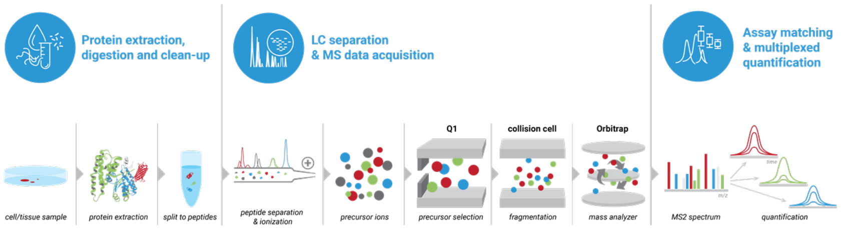

Figure 1 DIA workflow.

2 Experimental Pipeline

The main experimental steps are shown below:

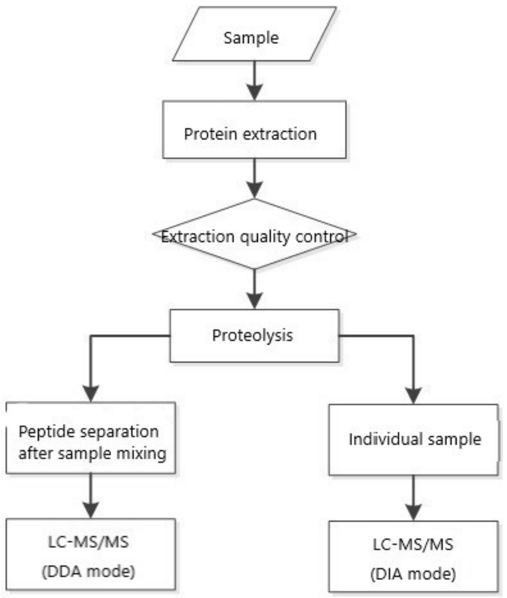

Figure 2 Experimental workflow.

2.1 Protein extraction

(1) Weigh appropriate amount of sample into a 1.5mL centrifuge tube;

(2) Add a 5mm steel bead and appropriate amount of Lysis Buffer 3, add PMSF with a final concentration of 1mM, EDTA with a final concentration of 2mM, vortex and let stand for 5 minutes, add DTT with a final concentration of 10mM;

(3) Oscillate with a tissue grinder for 2 minutes (frequency is 50HZ);

(4) 25,000g*4°C centrifugation for 20 minutes, take the supernatant;

(5) Add a final concentration of 10mM DTT, water bath at 56°C for 1 hour;

BGI Co., Ltd. 400-706-6615 ©2021 BGI All Rights Reserved.

(6) After returning to room temperature, a final concentration of 55mM IAM was added and incubated in a dark room for 45 minutes;

(7) Add cold acetone by 4 times volume of the sample, and stand at -20°C for 2 hours;

(8) Repeat step (7) until the supernatant is colorless, if necessary;

(9) 25,000g*4°C centrifugation for 20 minutes, discard the supernatant;

(10) Add an appropriate amount of Lysis Buffer 3 to precipitate, followed by ultrasonication to dissolve the precipitated proteins;

(11) After centrifugation at 25,000g*4°C for 20 minutes, the supernatant was taken for quantification.

2.2 Protein extraction quality control

(1)Bradford quantification

Standard proteins (0.2μg/μL BSA) 0, 2, 4, 6, 8, 10, 12, 14, 16, 18μL were sequentially added to the 96-well microtiter plates A1 to A10, followed by the addition of pure water 20, 18, 16, 14, 12, 10, 8, 6, 4, 2μL, and then 180μL of Coomassie Brilliant Blue G-250 Quantitative Working Solution was added to each well. The OD595 was measured with a microplate reader, and a linear standard curve was drawn based on the OD595 and protein concentration. Diluted the protein solution to be tested several times, added 180μL of the quantitative working solution to 20μL of the protein solution, and read at OD595. The sample protein concentration was calculated from the standard curve and sample OD595.

(2) SDS-PAGE

Each 10μg of protein solution was mixed with an appropriate amount of loading buffer, heated at 95°C for 5 minutes, centrifuged at 25,000g for 5 minutes, and the supernatant was loaded into a well of a 12% SDS polyacrylamide gel. 120V constant pressure electrophoresis for 120 minutes;

After electrophoresis, Coomassie blue staining was carried out for 2 hours, after which an appropriate amount of decolorizing solution (40% ethanol 10% acetic acid) was added to the shaker to decolorize for 3 to 5 times for 30 minutes each time.

2.3 Protein enzymatic hydrolysis

(1) Take 100μg of protein solution per sample and dilute with 50mM NH4HCO3 by 4 times volumes;

(2) Add 2.5μg of Trypsin enzyme in the ratio of protein: enzyme = 40:1, and digest for 4 hours at 37°C;

(3) Enzymatic peptides were desalted using a Strata X column and vacuumed to dryness.

2.4 High pH RP separation

Equal amount of peptides were extracted from all samples to mix, and the mixture was diluted with mobile phase A (5% ACN pH 9.8) and injected. The Shimadzu LC-20AB HPLC system coupled with a Gemini high pH C18 column (5μm, 4.6 x 250mm) was used. The sample was subjected to the column

 ©2021 BGI All Rights Reserved.

and then eluted at a flow rate of 1mL/min by gradient: 5% mobile phase B (95% ACN, pH 9.8) for 10 minutes, 5% to 35% mobile phase B for 40 minutes, 35% to 95% mobile phase B for 1 minute, flow Phase B lasted 3 minutes and 5% mobile phase B equilibrated for 10 minutes. The elution peak was monitored at a wavelength of 214nm and component was collected every minute. Components were combined into a total of 10 fractions, which were then freeze-dried.

2.5 DDA and DIA analysis by nano-LC-MS/MS

The dried peptide samples were reconstituted with mobile phase A (2% ACN, 0.1% FA), centrifuge at 20,000g for 10 minutes,and the supernatant was taken for injection. Separation was carried out by a Thermo UltiMate 3000 UHPLC liquid chromatograph. The sample was first enriched in the trap column and desalted, and then entered a tandem self-packed C18 column (150μm internal diameter, 1.8μm column size, 35cm column length), and separated at a flow rate of 500nL/min by the following effective gradient: 0~5 minutes, 5% mobile phase B (98% ACN, 0.1% FA); 5~130 minutes, mobile phase B linearly increased from 5% to 25%; 130~150 minutes, mobile phase B rose from 25% to 35%; 150~160 minutes, mobile phase B rose from 35% to 80%; 160~175 minutes, 80% mobile phase B; 175~175.5 minutes, mobile phase B decreased from 80% to 5%; 175.5~180 minutes, 5% mobile phase B. The nanoliter liquid phase separation end was directly connected to the mass spectrometer as the following settings.

For DDA analysis, LC separated peptides were ionized by nanoESI and injected to tandem mass spectrometer Fusion Lumos (Thermo Fisher Scientific, San Jose, CA) with DDA (data-dependent acquisiton) detection mode. The main settings were: ion source voltage 2kV; MS scan range 350~1,500m/z; MS resolution 60,000, maximal injection time (MIT) 50ms; MS/MS collision type HCD, collision energy NCE 30; MS/MS resolution 15,000, MIT 50ms, dynamic exclusion duration 30 seconds. The start m/z for MS/MS was fixed to 100. Precursor for MS/MS scan satisfied: charge range 2+ to 6+, top 30 precursors with intensity over 2E4. AGC was: MS 3E6, MS/MS 1E5.

For DIA analysis, LC separated peptides were ionized by nanoESI and injected to tandem mass spectrometer Fusion Lumos (Thermo Fisher Scientific, San Jose, CA) with DIA (data-independent acquisiton) detection mode. The main settings were: ion source voltage 2kV; MS scan range 400~1,500m/z; MS resolution 60,000, MIT 50ms; 400~1,500m/z was eqaully divided to 44 continuous windows MS/MS scan. MS/MS collision type HCD, MIT 54ms. Fragment ions were scanned in Orbitrap, MS/MS resolution 30,000, collision energy 30; AGC was 5E4.

3 Bioinformatic Analysis Pipeline

This process is based on the sample data generated from a high-resolution mass spectrometer. DDA data was identified by Andromeda search engine within MaxQuant, and identification results were used for spectral library construction. For large-scale DIA data, mProphet algorithm was used to complete analytical quality control, thus obtaining a large number of reliable quantitative results. This pipeline also performed GO, COG, Pathway functional annotation analysis and time series analysis. Based on the quantitative results, the differential proteins between comparison groups were found, and finally function enrichment analysis, protein-protein interaction (PPI) and subcellular localization analysis of the differential proteins were performed.

BGI Co., Ltd. 400-706-6615 ©2021 BGI All Rights Reserved.

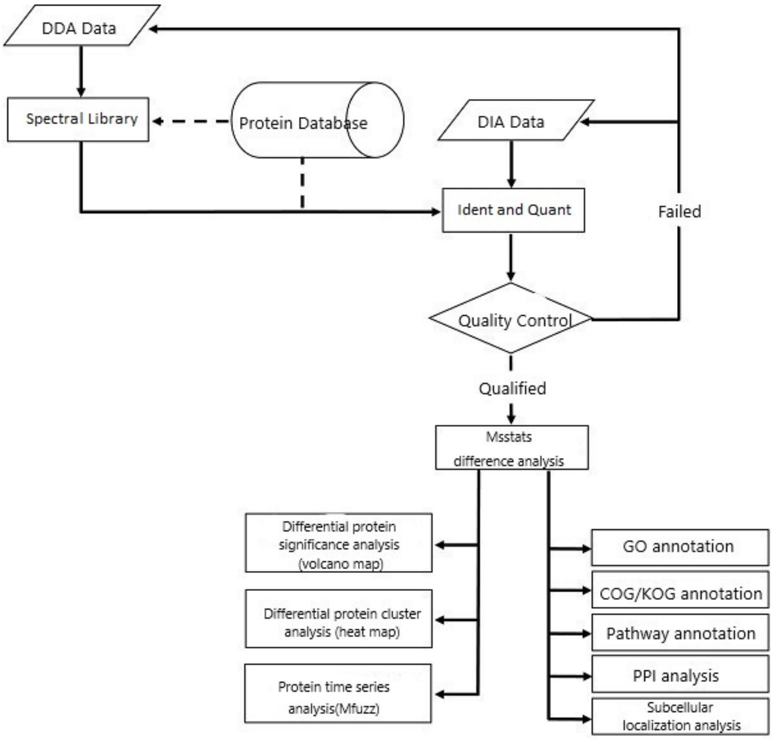

Figure 3 Bioinformatics Pipeline.

3.1 Database selection

The selection of database is an important step in MS based protein identification, and the final identified protein sequences are from the selected database.

Currently databases in use can be divided into three main categories:

1) UniProt protein database

UniProt is the most informative and resourceful protein database. It consists of data from three major databases, i.e. Swiss-Prot, TrEMBL and PIR-PSD. It is a data set verified by experts and consists of two parts: UniProtKB/Swiss-Prot (with reviewed, manually annotated entries) and UniProtKB/TrEMBL (with unreviewed, automatically annotated entries). In general, it is recommended to give priority using the subset of UniProtKB/Swiss-Prot for protein identification. When it aims to find novel sequences (such as alternative splicing, new transcripts) or to identify allied species, UniProtKB/TrEMBL datatbase can be considered .

2) The protein databases based on genome annotation

The databaseses mainly include a series of databases derived from NCBI and Ensembl gene annotation databases.

Among them, we choose protein database from reference sequence (RefSeq) of NCBI, which is a non-redundant proteome database. It is widely used in the analysis of multi-omics studies due to the importance of the NCBI annotation system. NCBI's RefSeq provides reference sequence for molecules that are naturally involved in central dogma, from chromosomes to mRNA and proteins. The RefSeq standard provides a basis for functional annotation of the human genome. It provides a stable reference for mutation analysis, gene expression studies, and polymorphic discovery.

©2021 BGI All Rights Reserved.

In addition, NCBI provides completed non-redundant protein sequence database (NCBI_nr), including animal, plant, microbial, bacterial and other taxonomy. Since the database is derived from various sources (including GeneBank, RefSeq, SwissProt, PDB, etc.), unless the species is without complete genome annotation, or it is necessary to search for homologous, it is not recommended to use this database for protein identification.

Ensembl aims to develop a software package with automatic annotation and maintenance for the eukaryotic genome. Ensembl has relatively complete and consistent genomic, transcriptome, and proteomic annotation information, which is ideal for multi omics analysis.

3) Databases from other sources

They usually refer to the protein target databases provided by the client, or new gene sequence generated from genome or transcriptome sequencing de novo assembly. They may also contain sequences of new features such as alternative splicing, mutation site, fusion genes, etc.

3.2 DDA data analysis

MaxQuant[2](http://www.maxquant.org) is a free protein identification and quantification software developed by Max Planck Institutes for high-resolution MS data. This project was executed using this software for identification of DDA data, served as a spectrum library for subsequent DIA analysis. The analysis used raw data as input files, and set corresponding parameters and databases, then performed identification and quantitative analysis. The identified peptides satisfies FDR <=1% will be used to construct the final spectral library.

During the identification of this project, the parameters were configurated as follows:

Table 1 MaxQuant Parameters Configuration (Download)

Item	Value
MaxQuant	1.5.3.30
Enzyme	Trypsin
Minimal peptide length	7
PSM-level FDR	0.01
Protein FDR	0.01
Fixed modifications	Carbamidomethyl (C)
Variable modifications	Oxidation (M); Acetyl (Protein N-term); Gln->pyro-Glu (N-term Q); Deamidated (NQ)
Database	Uniprot_sau-ab_irt.fasta (262117 sequences)

3.3 DIA data analysis

The DIA data was analyzed using the iRT peptides for retention time calibration. Then, based on the target-decoy model applicable to SWATH-MS, false positive control was performed with FDR 1%, therefore obtaining significant quantitative results.

3.4 MSstats differential analysis

MSstats[3] is an R package from the Bioconductor repository. It can be used for statistical evaluation of significant differences in proteins or peptides from different samples, and is widely used in targeted proteomics MRM, label free quantitation, and SWATH quantitative experiments. The core algorithm is linear mixed effect model. The process preprocessed the data according to the predefined comparison group, and then performed the significance test based on the model. Thereafter,

©2021 BGI All Rights Reserved.

differential protein screening was performed based on the fold change >1.5 and Pvalue<0.05 as the criterion for the significant difference. At the same time, the enrichment analysis is performed on the differential proteins.

● Help

1 Protein Sequence: FASTA Format

Text-based FASTA format files are used to store DNA or protein sequence. The first line of the sequence file begins with the symbol ">", followed by the sequence ID, which can then be followed by sequence annotation information. The second line is the DNA base sequence or protein amino acid sequence corresponding to the first row. FASTA files can be opened directly with WordPad.

An example is as follows:

```
>tr|F2N3W2|F2N3W2_PSEU6 Quinoprotein alcohol dehydrogenase OS=Pseudomonas stutzeri (strain DSM 4166 / CMT.9.A) GN=PSTAA_2293 PE=4 SV=1
MKHTGLRKPFAVTALCAAVAMSSLHAWAVTDQEILNDAKSTDQIVTNGLGLQGQRYSTLD
ALNTNNINQLRPVWGFSLGGEKQRGQEAQPLIKDGVMYITGSYSRVYALDARTGKELWQ
YDARLPDGIMPCCDVINRGVALYDDLVIFGTLDAKLVALNKDTGKVVWKKTVADYKAGY
SLTAAPLVVN
```

2 File Format Description of all_peptideSummary.xls

This file contains peptide information identified from the DDA data using MaxQuant. Each column is Tab-delimitated.

Table 1 Format description of peptide list in library (See all)

Field	Description
Sequence	Peptide sequence
Modifications	Modification
Mass	Peptide mass
Mass Fractional Part	Decimal part exact value of peptide mass
Protein Groups	Protein group number
Proteins	All protein IDs corresponding to the peptides
Unique (Groups)	When labeled as "+" or "yes", it indicates that the peptide is only unique to a specific protein group
Unique (Proteins)	When labeled as "+" or "yes", it indicates that the peptide is only unique to a specific protein
Acetyl (Protein N-term)	The number of Acetyl (Protein N-term) site in this peptide
Oxidation (M)	The number of Oxidation (M) site in this peptide
Missed cleavages	Number of missing cleavages
Retention time	Retention time
Calibrated retention time	Calibrated retention time
Charges	Carried charge of a peptide/fragment
PEP	Posterior Error Probability, the smaller the value, the better
MS/MS scan number	Spectral scan number corresponding to the highest scored identified peptide
Raw file	The raw file name corresponding to the scan
Score	Score of highest scored peptide
Delta score	Score difference between the highest match and the second highest match
Intensity	Peptide intensity (XIC)

 ©2021 BGI All Rights Reserved.

3 File Format Description of proteinGroups_recalibration.txt

This file contains protein information identified from the DDA data using MaxQuant. Each column is Tab-delimitated.

Table 2 Format description of protein list in library (See all)

Field	Description
Primary_protein_ID	The primary protein ID in the group
Protein_group_IDs	All protein IDs in the group
Peptide counts (all)	Number of peptides to the related protein (all peptides)
Peptide counts (razor+unique)	Number of peptides to the related protein (razor+unique peptides)
Peptide counts (unique)	Number of peptides to the related protein (unique peptide)
Fasta headers	Fasta sequence title
Number of proteins	Number of protein IDs in the group
Peptides	Number of peptides in the group (all peptides)
Razor + unique peptides	Number of peptides in the group (razor+unique peptides)
Unique peptides	Number of peptides in the group (unique peptide)
Sequence coverage [%]	Protein sequence coverage (all peptides)
Unique + razor sequence coverage [%]	Protein sequence coverage (razor+unique peptides))
Unique sequence coverage [%]	Protein sequence coverage (unique peptide)
Mol. weight [kDa]	Protein molecular weight
Sequence length	Sequence length of primary protein
Sequence lengths	Sequence lengths of all proteins in the group
Q-value	Q-value (FDR statistic)
Score	Protein score
Intensity	Protein intensity
MS/MS Count	The total number of MS2 spectra in each sample

4 File Format Description of annotation_allprotein.xls

This file contains protein information identified in this DIA experiment. Each column is Tab-delimitated.

Table 3 Format description of DIA identified protein annotation list (See all)

BGI Co., Ltd. 400-706-6615 ©2021 BGI All Rights Reserved.

Field	Description
Protein	Protein ID
ProteinGroup	Protein group ID
PG_Cscore	Protein group identification score, the higher the more confident
PG_Qvalue	Protein groups Qvalue for FDR control, the threshold is set to 0.01
Description	Protein description
Nr_Identity	Nr library alignment identity score
Nr_E-value	Nr library alignment probability score, the lower the more confident, the threshold is set to 1e-05
Nr_Accession	Nr library alignment best match protein ID
Nr_Description	Nr library alignment best match protein description
Swissprot Identity	Swissprot library alignment identity score
Swissprot E-value	Swissprot library alignment probability score, the lower the more confident, the threshold is set to 1e-05
Swissprot Accession	Swissprot library alignment best match protein ID
Swissprot Description	Swissprot library alignment best match protein description
Protein_or_Domain	Proteins or domains matched to COG or KOG annotation
COG/KOG Score	COG or KOG database alignment scores, the higher the more confident
COG/KOG E-value	COG or KOG database alignment probability, the smaller the more credible, the threshold is set to 1e-05
COG/KOG ID	COG or KOG annotation ID, only the best match is listed here, please refer to the annotation result files for all annotations
COG/KOG Function-Description	COG or KOG annotation ID description
COG/KOG Code	Abbreviation for the functional categories of COG or KOG ID
COG/KOG Function-Categories	Full name for the functional category of COG or KOG ID

5 File Format Description of XX2-VS-XX1.All.xls

This document contains all differential proteins information after statistical analysis of XX2 sample and XX1 sample. Each column is Tab-delimitated.

Table 4 Format description of differential proteins from different comparison groups (See all)

BGI Co., Ltd. 400-706-6615 ©2021 BGI All Rights Reserved.

Field	Description
Protein	Protein ID
Label	Compare group name
log2FC	Log2 value of fold change
SE	Standard error
Tvalue	T test T value
DF	T test degree of freedom
pvalue	P value
adj_pvalue	Adjusted P value (FDR)
Class	Classification. 'None' means no difference, 'Up' means significant up regulation, and 'Down' means significant down regulation
ProteinGroup	Proteome ID
Description	Protein description
Nr_Identity	Nr library alignment identity score
Nr_E-value	Nr library alignment probability score, the lower the more confidant, the threshold is set to 1e-05
Nr_Accession	Nr library alignment best match protein ID
Nr_Description	Nr library alignment best match protein description
Swissprot Identity	Swissprot library alignment identity score
Swissprot E-value	Swissprot library alignment probability score, the lower the more confidant, the threshold is set to 1e-05
Swissprot Accession	Swissprot library alignment best match protein ID
Swissprot Description	Swissprot library alignment best match protein description
Protein_or_Domain	Proteins or domains matched to COG or KOG annotation

6 File Format Description of dia-proteinSummary.xls

This file contains relative quantitation values for proteins of all samples. Each column is Tab-delimitated.

Table 5 Format description of relative quantitative values for proteins of all samples (Download)

Field	Description
Protein	Protein ID
Case_1	The relative quantitative value of the sample Case_1 protein, the value is obtained by sample normalization and log2 transformation
Case_2	The relative quantitative value of the sample Case_2 protein, the value is obtained by sample normalization and log2 transformation
Case_3	The relative quantitative value of the sample Case_3 protein, the value is obtained by sample normalization and log2 transformation
Control_1	The relative quantitative value of the sample Control_1 protein, the value is obtained by sample normalization and log2 conversion
Control_2	The relative quantitative value of the sample Control_2 protein, the value is obtained by sample normalization and log2 conversion
Control_3	The relative quantitative value of the sample Control_3 protein, the value is obtained by sample normalization and log2 conversion
ProteinGroup	Protein group ID

7 File Format Description of dia-peptideSummary.xls

This file contains relative quantitation values for peptides of all samples. Each column is Tab-delimitated.

Table 6 Format description of relative quantitative values for peptides of all samples (Download)

BGI Co., Ltd. 400-706-6615 ©2021 BGI All Rights Reserved.

Field	Description
PeptideSequence	Peptide sequence
PrecursorCharge	Precursor charge state
ProteinGroup	Peptide associated protein group ID
Case_1	The relative quantitative value of the sample Case_1 peptide, the value is obtained by sample normalization and log2 transformation
Case_2	The relative quantitative value of the sample Case_2 peptide, the value is obtained by sample normalization and log2 transformation
Case_3	The relative quantitative value of the sample Case_3 peptide, the value is obtained by sample normalization and log2 transformation
Control_1	The relative quantitative value of the sample Control_1 peptide, the value is obtained by sample normalization and log2 conversion
Control_2	The relative quantitative value of the sample Control_2 peptide, the value is obtained by sample normalization and log2 conversion
Control_3	The relative quantitative value of the sample Control_3 peptide, the value is obtained by sample normalization and log2 conversion

8 How to Read Report of Clustering Analysis

Each cluster plan which is consisted of more than two pairwises, has two types clustering results: intersection and union.

BGI Co.， Ltd. 400-706-6615 ©2021 BGI All Rights Reserved.

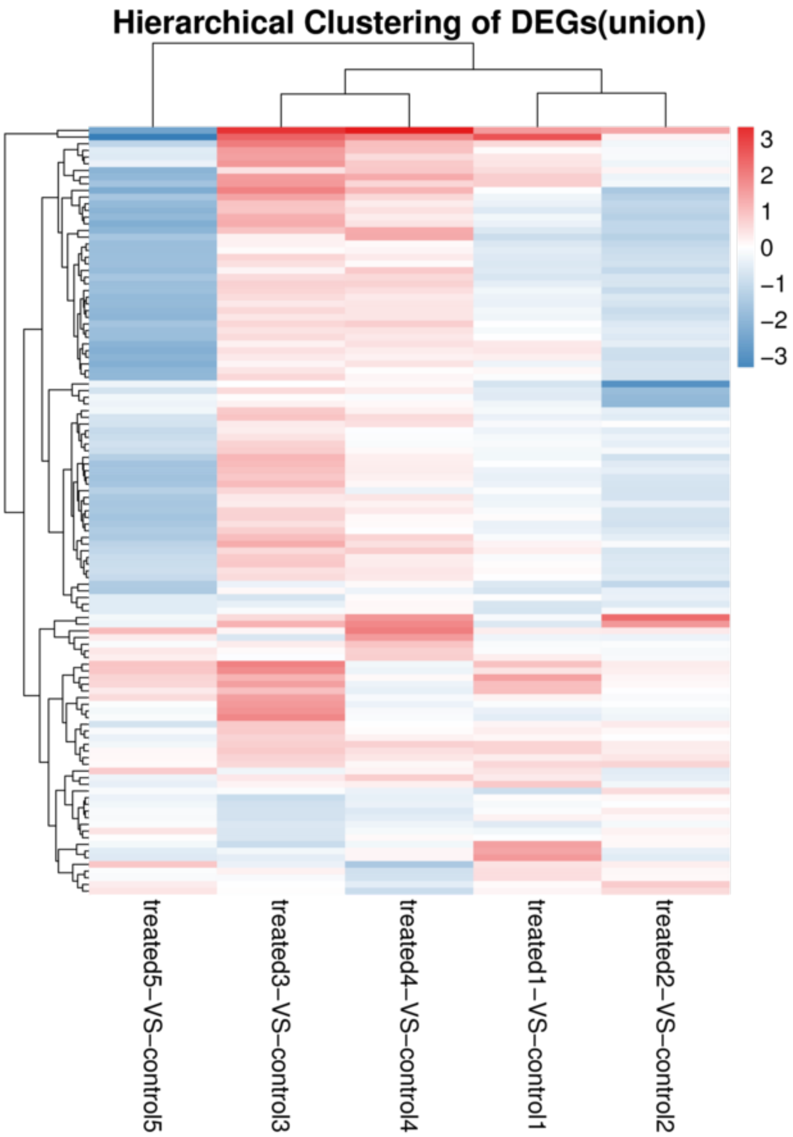

Figure 1 Heat map of DEPs expression clustering.

Each column in the figure represents a comparison group (e.g., exp1-VS-exp2), and each row represents a protein. The Log2(Foldchange) value of the protein is shown in a heat map in different colors, with red representing up regulation and blue representing down regulation. In addition to proteins clustering, the comparison group is clustered; the proteins or comparison groups with close expression pattern are grouped.

9 File Format Description of Time Series Analysis Results

Table 7 File format description of *.average.xls (Download)

Field	Description
Protein	Protein ID
control	The quantitative value of proteins in control samples, if there are multiple replicates, use the average value
case1	The quantitative value of proteins in case 1 samples, if there are multiple replicates, use the average value
case2	The quantitative value of proteins in case 2 samples, if there are multiple replicates, use the average value

Table 8 File format description of *.cluster.xls (Download)

BGI Co., Ltd. 400-706-6615 ©2021 BGI All Rights Reserved.

Field	Description
Protein	Protein ID
cluster	Cluster ID which the protein is in
membership	The value range is 0~1, it is used to evaluate the similarity between the protein and a cluster. The closer the value is to 1, the more consistent trend between the protein and the cluster

10 How to Read Report of GO Functional Annotation and Enrichment Results

The GO functional annotation results are packaged in sample_GO.zip under Function_analysis directory, and sample.fa.GO.png is the GO classification graph, sample.fa.GO2protein.xls is the GO classification file (stores relations between GO entries and proteins), the file can be opened with Excel (Figure2).

	A	B	C	D	E	F
1	Ontology	Class	number_of_Nb	proteins_of_Nb;		
2	biological_process	biological regulation	101	Niben101Scf03422g01002.1;		

Figure 2 GO2protein demo.

Table 9 Format description of GO2protein (Download)

Field	Description
Ontology	Gene Ontology aspect (biological_process or cellular_component or molecular_function)
Class	GO entries
number_of_*	Number of proteins in each GO entry
Proteins_of_*	Protein IDs in each GO entry

Sample.fa.protein2GO.xls is the GO annotation (relations between proteins and GO entries) file, which can be opened with Excel.

	A	B	C	D
1	Niben101Ctg05133g00002.1	GO:0008614	GO:0009108	

Figure 3 protein2GO demo.

Table 10 Format description of protein2GO (Download)

Field	Description
First column	Proetein ID
Second column and after	GO ID

The results of GO enrichment to DEPs are packaged in GO_Enrichment.zip under Differential_enrichment directory. The results can be viewed in IE browser by opening the web page result GOView.html. The left navigation bar includes three aspects for GO entries (C: cellular component, P: biological process, F: molecular function). Click one of them and the results of GO enrichment are shown in figure{5}.

BGI Co., Ltd. 400-706-6615 ©2021 BGI All Rights Reserved.

Result Table

Terms from the Component Ontology with p-value as good or better than 1

Gene Ontology term	Cluster frequency	Protein frequency of
extracellular region	120 out of 264 genes, 45.5%	894 out of 7481 genes, 1
extracellular region part	96 out of 264 genes, 36.4%	564 out of 7481 genes, 7
extracellular space	72 out of 264 genes, 27.3%	423 out of 7481 genes, 5
extracellular matrix	59 out of 264 genes, 22.3%	280 out of 7481 genes, 3
proteinaceous extracellular matrix	47 out of 264 genes, 17.8%	223 out of 7481 genes, 3

Figure 4 GO entries of DEPs' enrichment.

The first column is the GO entry, the second column is the number and proportion of DEPs annotations to the GO entry, the third column is the number and proportion of all identified proteins annotations to the GO entries, and the fourth column is the P-value of hypergeometric test, the lower the value, the more significant the GO entry enrichment. When P-value<0.05, the GO entry is significantly enriched.

Click the GO entry 'BLOC complex' inFigure5, it will automatically jump to http://amigo.geneontology.org/amigo for more detailed annotation information when connected to internet. Click on 'view genes' to query the protein IDs of annotation to the GO entry, as shown in Figure5.

BLOC complex	63915, 100526837
cytosol	63915, 100526837

Figure 5 Protein IDs annotated to a GO entry.

Two differential proteins are annotated to the GO entry 'BLOC complex'.

11 How to Read Report of COG/KOG Functional Annotation Results

The COG/KOG annotation results are packaged in sample_COG/KOG.zip under Function_analysis directory, sample.cog/kog2protein.xls is the COG or KOG classification file (relations between COG/KOG entries and proteins). This file can be opened with Excel.

	A	B	C	D	E
1	Code	Functional-Categories	Protein-Number	Proteins	
2	A	RNA processing and modi:	1	Niben101Scf07457g0	
3	B	Chromatin structure and	3	Niben101S	Niben101S
4	C	Energy production and c	124	Niben101S	Niben101S

Figure 6 COG/KOG2protein demo.

Table 11 Format description of COG/KOG2protein (Download)

Field	Description
Code	COG or KOG Code
Functional-Categories	COG or KOG function categories
Protein-Number	Number of proteins in each COG or KOG function category
Proteins	Protein IDs in each COG or KOG function category

The sample.protein2COG/KOG.xls is a COG or KOG annotation (relations between proteins and COG or KOG entries) file that can be opened with Excel.

	A	B	C	D	E	F	G	H	I
1	Protein	Protein-or-Domain	Score	E-Value	COG-ID	Function-Descript	Code	Functional-Categories	
2	Niben101Scf1	CT539	95.1	3.00E-20	COG0526	Thiol-disulfide i	OC	Posttranslational moc	
3	Niben101Scf1	CPn0659	93.2	1.00E-19	COG0526	Thiol-disulfide i	OC	Posttranslational moc	

BGI Co., Ltd. 400-706-6615 ©2021 BGI All Rights Reserved.

Figure 7 protein2COG/KOG demo.

Table 12 Format description of protein2COG/KOG （Download)

Field	Description
Protein	Protein IDs
Protein-or-Domain	COG or KOG annotated protein or domain
Score	COG or KOG blast score
E-Value	COG or KOG blast E-value
COG/KOG-ID	COG or KOG ID
Function-Description	COG or KOG function description
Code	COG or KOG function category code
Functional-Categories	COG or KOG function categories

12 How to Read Report of Pathway Functional Annotation and Enrichment Results

The pathway functional annotation results are packaged in sample_Pathway.zip under Function_Analyse directory. sample.ko is a list of protein IDs and related KO numbers (this file can be opened with Excel, or it can be viewed with less command in terminal).

	A	B
1	# Method: BLAST	Condition: expect <= 1e-05; rank <= 5
2	# Summary:	1651 succeed, 53 fail
3		
4	# query	ko_id:rank:evalue:score:subject_id:ko_definition
5	sp|A1A535|MELT_MOUSE	K09566|1|1e-51|202|pxy:105383147|peptidyl-prolyl isomerase G (cyclophilin G) [EC:5.2.1.8]
6	sp|A2A432|CUL4B_MOUSE	K10609|1|0.0|2013|mmu:72584|cullin 4
7	sp|A2A884|ZEP3_MOUSE	K09239|1|0.0|4673|mmu:16656|human immunodeficiency virus type I enhancer-binding protein
8	sp|A2A8L5|PTPRF_MOUSE	K05695|1|0.0|3937|mmu:19268|receptor-type tyrosine-protein phosphatase F [EC:3.1.3.48]
9	sp|A2A8Z1|OSBL9_MOUSE	K20465|1|0.0|1477|pon:100172780|oxysterol-binding protein-related protein 9/10/11
10	sp|A2AJ88|PLPL7_MOUSE	K14676|1|0.0|2802|mmu:241274|lysophospholipid hydrolase [EC:3.1.1.5]

Figure 8 *.ko demo file.

Table 13 Format description of *.ko （Download)

Field	Description
First to third row	Description of analysis process
First column	Protein
Second column	KEGG Orthology

sample.path is a pathway list file for proteins (this file can be opened with Excel, or it can be viewed with less command in terminal).

	A	B	C	D	E
1	#Pathway	Count (14	Pathway ID	Proteins	KOs
2	Metabolic pathways	83	ko01100	Niben101C	K13811+K08
3	Biosynthesis of seco	52	ko01110	Niben101S	K00600+K01
4	Microbial metabolism	47	ko01120	Niben101C	K13811+K0(
5	Biosynthesis of anti	47	ko01130	Niben101C	K13811+K0(
6	Carbon metabolism	38	ko01200	Niben101S	K00600+K0(
7	Biosynthesis of amin	25	ko01230	Niben101S	K00600+K01
8	Glyoxylate and dicar	18	ko00630	Niben101S	K00600+K0(
9	Glycolysis / Glucone	17	ko00010	Niben101S	K00128+K0(
10	Pyruvate metabolism	13	ko00620	Niben101S	K00026+K0(

Figure 9 *.path demo file.

Table 14 Format description of *.path （Download)

 ©2021 BGI All Rights Reserved.

Field	Description
Pathway	Pathway name
Count	Number of proteins in each pathway entry
Pathway ID	Pathway ID in KEGG database
Proteins	Proteins annotated to the pathway entry
KOs	KEGG Orthology of the pathway entry

Open web report for pathway enrichment result and the enriched KEGG pathways are listed as Figure10:

1. F-VS-E

#	Pathway	Diff Proteins with pathway annotation (273)	All Proteins with pathway annotation (687
1	Primary immunodeficiency	26 (9.52%)	126 (1.83%)
2	Staphylococcus aureus infection	18 (6.59%)	139 (2.02%)
3	Cytokine-cytokine receptor interaction	10 (3.66%)	46 (0.67%)
4	Dilated cardiomyopathy	19 (6.96%)	155 (2.25%)
5	TGF-beta signaling pathway	11 (4.03%)	59 (0.86%)
6	Complement and coagulation cascades	14 (5.13%)	97 (1.41%)
7	African trypanosomiasis	13 (4.76%)	87 (1.27%)
8	ECM-receptor interaction	16 (5.86%)	130 (1.89%)
9	Intestinal immune network for IgA production	13 (4.76%)	92 (1.34%)
10	Amoebiasis	17 (6.23%)	167 (2.43%)
11	Hematopoietic cell lineage	14 (5.13%)	132 (1.92%)
12	p53 signaling pathway	8 (2.93%)	57 (0.83%)
13	Rheumatoid arthritis	12 (4.4%)	114 (1.66%)
14	Systemic lupus erythematosus	14 (5.13%)	147 (2.14%)
15	Autoimmune thyroid disease	11 (4.03%)	104 (1.51%)
16	PPAR signaling pathway	8 (2.93%)	68 (0.99%)
17	NF-kappa B signaling pathway	12 (4.4%)	133 (1.93%)
18	Asthma	9 (3.3%)	87 (1.27%)
19	Butirosin and neomycin biosynthesis	2 (0.73%)	4 (0.06%)
20	Hypertrophic cardiomyopathy (HCM)	10 (3.66%)	106 (1.54%)
21	Protein digestion and absorption	9 (3.3%)	93 (1.35%)
22	Bladder cancer	5 (1.83%)	35 (0.51%)
23	B cell receptor signaling pathway	12 (4.4%)	144 (2.09%)
24	Fc epsilon RI signaling pathway	11 (4.03%)	127 (1.85%)
25	Transcriptional misregulation in cancer	14 (5.13%)	184 (2.68%)
26	Natural killer cell mediated cytotoxicity	12 (4.4%)	149 (2.17%)
27	Focal adhesion	19 (6.96%)	280 (4.07%)
28	Tuberculosis	15 (5.49%)	209 (3.04%)
29	Allograft rejection	9 (3.3%)	102 (1.48%)
30	Malaria	5 (1.83%)	40 (0.58%)

Figure 10 Pathway enrichment analysis of DEPs.

The first column is ordinal number. The second column is pathway name. The third column is the number (proportion) of DEPs enriched to this pathway. The fourth column is the number (proportion) of all identified proteins enriched to this pathway. The fifth column is the P-value of hypergeometric test, P-value < 0.05 indicates the significant pathway enrichment. The smaller the P-value is, the more significant the enrichment is. The sixth column is the Pathway ID.

Click pathway name "Vascular smooth muscle contraction" in Figure10, you can get protein IDs that enriched to it as Figure11:

#	Pathway	Proteins
1	Vascular smooth muscle contraction	comp31440_c0, comp40793_c0, comp143365_c0, comp157414_c0, comp57197_c0, comp17147_c0, comp26953_c0
2	Proximal tubule bicarbonate reclamation	comp10038_c0, comp40731_c0, comp48142_c0
3	Pancreatic secretion	comp31440_c0, comp40793_c0, comp10038_c0, comp40731_c0, comp44480_c0
4	Amino sugar and nucleotide sugar metabolism	comp47641_c0, comp52792_c0, comp51078_c0
5	Regulation of actin cytoskeleton	comp31440_c0, comp40793_c0, comp143365_c0, comp157414_c0, comp57197_c0, comp17147_c0
6	Pertussis	comp31440_c0, comp26953_c0
7	Bile secretion	comp10038_c0, comp40731_c0, comp47520_c0

Figure 11 Protein ID list enriched to pathway.

There are 7 DEPs enriched to the pathway "Vascular smooth muscle contraction".

BGI Co., Ltd. 400-706-6615 ©2021 BGI All Rights Reserved.

After detecting the most significant enriched pathway of DEPs, we can view detailed pathway map via pathway ID. For example, click the hyperlink on "Vascular smooth muscle contraction" in Figure11, it will automatically jump to pathway map as shown in Figure12.

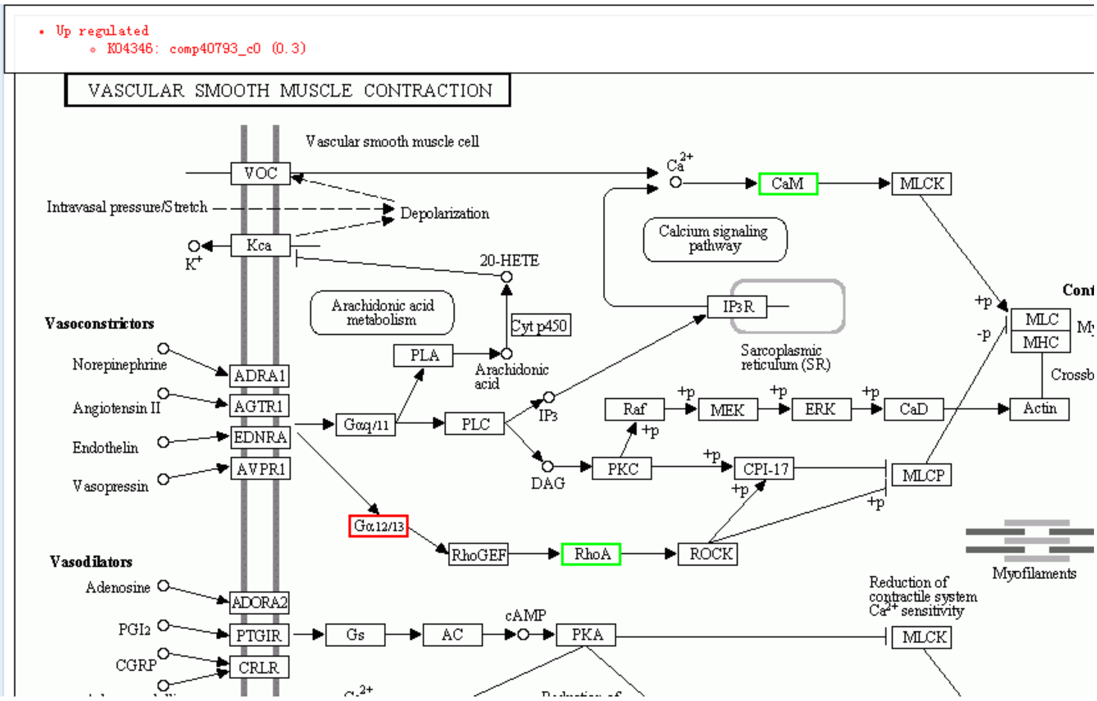

Figure 12 KEGG Pathway Map "Vascular smooth muscle contraction".

Significant up regulation proteins are marked with red rectangles and significant down regulation proteins are marked with green rectangles. When mouse hover on red or green rectangles, the related DEPs and log2 fold change will appear on the top left. Click protein name in the figure, the page will redirect to KEGG website when it is online.

13 How to Read Report of Protein-protein Interaction Results

The protein-protein interaction results are packaged in PPI.zip under Differential_enrichment directory. network.relation.xls is the result file of the interaction of the proteins, which can be opened with Excel.

	A	B	C	D	E
1	protein1	protein2	protein_cluster1	protein_cluster2	score
2	sp|Q29865|1C18_HUMAN	sp|P13761|2B17_HUMAN	9598.ENSPTRP00000054564	9598.ENSPTRP00000030734	
3	sp|Q29865|1C18_HUMAN	sp|Q9TQE0|2B19_HUMAN	9598.ENSPTRP00000054564	9598.ENSPTRP00000030734	
4	sp|P08567|PLEK_HUMAN	sp|P10144|GRAB_HUMAN	9606.ENSP00000234313	9606.ENSP00000216341	
5	sp|P50591|TNF10_HUMAN	sp|P10144|GRAB_HUMAN	9606.ENSP00000241261	9606.ENSP00000216341	
6	sp|P50591|TNF10_HUMAN	sp|P08567|PLEK_HUMAN	9606.ENSP00000241261	9606.ENSP00000234313	

Figure 13 PPI demo result.

Table 15 Format description of PPI (Download)

BGI Co., Ltd. 400-706-6615 ©2021 BGI All Rights Reserved.

Field	Description
protein1	Differentially expressed protein 1
protein2	Differentially expressed protein 2
protein_cluster1	Protein cluster 1 in the STRING database aligned for differentially expressed protein 1
protein_cluster2	Protein cluster 2 in the STRING database aligned for differentially expressed protein 2
score	Interaction scores of differentially expressed protein 1 and 2

14 How to Read Report of Subcellular Localization

The subcellular localization results are packaged in Subcellular.zip under Differential_enrichment directory. subcellular2protein.xls is a differential protein subcellular localization classification file (relations between subcellular localization and proteins), which can be opened with Excel.

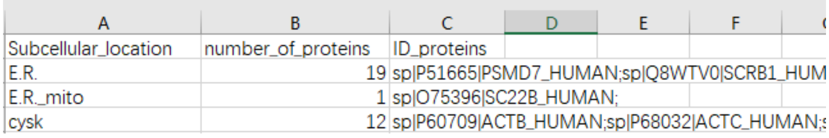

A	B	C	D	E	F
Subcellular_location	number_of_proteins	ID_proteins			
E.R.	19	sp|P51665|PSMD7_HUMAN;sp|Q8WTV0|SCRB1_HUM			
E.R._mito	1	sp|O75396|SC22B_HUMAN;			
cysk	12	sp|P60709|ACTB_HUMAN;sp|P68032|ACTC_HUMAN;			

Figure 14 Subcellular2protein demo file .

Table 16 Format description of subcellular2protein (Download)

Field	Description
Subcellular_location	Subcellular location
number_of_proteins	Number of proteins
ID_proteins	ID of the proteins

protein2subcellular.xls is a differential protein subcellular localization file (the relations between proteins and subcellular localizations), which can be opened with Excel.

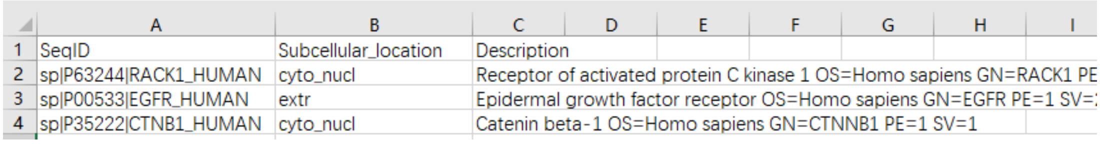

	A	B	C
1	SeqID	Subcellular_location	Description
2	sp|P63244|RACK1_HUMAN	cyto_nucl	Receptor of activated protein C kinase 1 OS=Homo sapiens GN=RACK1 PE
3	sp|P00533|EGFR_HUMAN	extr	Epidermal growth factor receptor OS=Homo sapiens GN=EGFR PE=1 SV=
4	sp|P35222|CTNB1_HUMAN	cyto_nucl	Catenin beta-1 OS=Homo sapiens GN=CTNNB1 PE=1 SV=1

Figure 15 protein2subcellular demo.

Table 17 Format description of protein2subcellular (Download)

Field	Description
SeqID	Protein ID
Subcellular_location	Subcellular location
Description	Protein description

WoLF PSORT subcellular localization results are represented by abbreviations. Detailed explanations of abbreviations are given in the following table:

Table 18 Abbreviation Index (Download)

BGI Co., Ltd.   400-706-6615   ©2021 BGI All Rights Reserved.

WoLF PSORT site	Description
chlo	chloroplast
cysk	cytoskeleton
cyto	cytosol
extr	extracellular
E.R.	endoplasmic reticulum
golg	Golgi apparatus
lyso	lysozyme
mito	mitochondria
nucl	nucleus
plas	plasma membrane
pero	peroxisome
vacu	vacuolar membrane

Localization classes including underscores indicate the possibility of double localization, for example "E.R._golg" indicates proteins are located in both endoplasmic reticulum and Golgi apparatus.

15 NR Database

The NR database is called the non-redundant database and is maintained by the National Center for Biotechnology Information (NCBI). It integrates multiple protein databases such as the GenBank CDS region translation sequence, the Refseq protein library, and the SwissProt protein database. The NR database is very comprehensive, but most proteins have not been validated.

16 Swiss-Prot Database

SWISS-PROT is a protein sequence database with detailed annotations. It is maintained by the European Bioinformatics Center (EBI) and has been incorporated into the UniProt database. It aims to help genomics and proteomics and related molecular biology researchers provide the latest information on protein sequences. The SWISS-PROT database contains a carefully checked and accurately annotated protein sequence in the EMBL nucleic acid sequence database and therefore has a high degree of confidence.

17 COG/KOG Database

The COG is called the Cluster of Orthologous Groups of proteins. Each COG entry contains a series of orthologs or paralogs. Orthologous proteins refer to proteins that have evolved from vertical families from different species and typically retain the same function as the original protein. Paralogous proteins are those proteins that are derived from gene replication in certain species and may evolve new functions related to the original (for prokaryotic organisms).

The KOG is called the Eukaryotic orthologous groups. Each KOG entry contains a series of orthologs or paralogs. An orthologous protein is a protein that has evolved from a vertical family from different species and specifically retains the same function as the original protein. Paralogous proteins are those proteins that are derived from gene replication in certain species and may evolve new functions related to the original (for eukaryotes).

18 GO Database

The full name of GO is Gene Ontology. The original intention of creating a gene ontology is to provide a working platform for a representative description of terminology or semantic interpretation of

 ©2021 BGI All Rights Reserved.

the characteristics of gene and gene products, enabling bioinformatics researchers to generalize, process, explain, share gene and gene product data.

The gene and gene product vocabularies involved in gene ontology are divided into three categories, covering three aspects of biology: 1) cellular component: each part of the cell and the extracellular environment. 2) Molecular function: can be described as a molecular level activity, such as catalytic or binding activity. 3) Biological process: Biological process refers to a series of events that result from the functional combination of one or more molecules.

19 KEGG Database

The whole name of KEGG is Kyoto Encyclopedia of Genes and Genomes. KEGG version 0.1 is published by Kanehisa Laboratories in 1995, and it is developed into an integrity database now. Its core database is KEGG PATHWAY database. KEGG database has advantage on the figures of metabolic pathways. For example, if we want to know the genes involved in alanine metabolic pathway, we could search "Alanine" in the annotation result (x.kegg.list.anno).

FAQs

In the comparison group difference file, why do some proteins have a difference ratio but the P-value is NA?

Calculating P-values generally requires a certain amount of data points, so that it is sufficient to construct a corresponding distribution for hypothesis testing. The described condition is likely to occur when certain proteins have quantitative values in only a few samples. Usually this protein is considered as a non-differentiating protein.

References

[1] Von Mering, Christian, et al. STRING: known and predicted protein鈥揺rotein associations, integrated and transferred across organisms. Nucleic acids research 33.suppl_1 (2005): D433-D437.

[2] Cox, Jürgen, and Matthias Mann. MaxQuant enables high peptide identification rates, individualized ppb-range mass accuracies and proteome-wide protein quantification. Nature biotechnology 26.12 (2008): 1367.

[3] Choi, Meena, et al. MSstats: an R package for statistical analysis of quantitative mass spectrometry-based proteomic experiments. Bioinformatics 30.17 (2014): 2524-2526.

Technical Support E-mail:info@bgitechsolutions.com
Website: www.bgitechsolutions.com

©2021 BGI All Rights Reserved.
